# Phenotypic and Genomic Local Adaptation across Latitude and Altitude in *Populus trichocarpa*

**DOI:** 10.1093/gbe/evz151

**Published:** 2019-07-22

**Authors:** Man Zhang, Haktan Suren, Jason A Holliday

**Affiliations:** 1 Department of Forest Resources and Environmental Conservation, Virginia Tech, Blacksburg, Virginia; 2 National Engineering Research Center for Floriculture, School of Landscape Architecture, Beijing Forestry University, China

**Keywords:** local adaptation, parallel evolution, population genomics, forest genetics

## Abstract

Local adaptation to climate allows plants to cope with temporally and spatially heterogeneous environments, and parallel phenotypic clines provide a natural experiment to uncover the genomic architecture of adaptation. Though extensive effort has been made to investigate the genomic basis of local adaptation to climate across the latitudinal range of tree species, less is known for altitudinal clines. We used exome capture to genotype 451 *Populus trichocarpa* genotypes across altitudinal and latitudinal gradients spanning the natural species range, and phenotyped these trees for a variety of adaptive traits in two common gardens. We observed clinal variation in phenotypic traits across the two transects, which indicates climate-driven selection, and coupled gene-based genotype–phenotype and genotype–environment association scans to identify imprints of climatic adaptation on the genome. Although many of the phenotype- and climate-associated genes were unique to one transect, we found evidence of parallelism between latitude and altitude, as well as significant convergence when we compared our outlier genes with those putatively involved in climatic adaptation in two gymnosperm species. These results suggest that not only genomic constraint during adaptation to similar environmental gradients in poplar but also different environmental contexts, spatial scale, and perhaps redundant function among potentially adaptive genes and polymorphisms lead to divergent adaptive architectures.

## Introduction

Predicting the evolutionary and ecological outcomes of environmental change requires an understanding of the genomic basis of quantitative, locally adaptive traits in order to assess the magnitude of allele frequency shifts necessary to track novel climatic regimes. If the underlying genotype–environment relationships are generalizable among related species that inhabit shared environments, such predictions could be advanced without exhaustive characterization of the genomic architecture of these traits at the species level. Convergent adaptation occurs when distantly related species independently develop similar phenotypes, whereas parallel adaptation refers to populations or closely related lineages evolving repeated patterns of phenotypic divergence in distinct geographical locations (Ralph and Coop 2010; Westram et al. 2014). The formation of phenotypic parallelism relies on standing variation, gene flow, and shared selective regimes (Faria et al. 2014; Westram et al. 2014). When shared standing genetic variation and adaptive loci are transmitted between populations or species, selection may target the same genetic loci to generate similar phenotypes across “replicate” environments (Westram et al. 2014). Factors such as demographic history, effective population size, extent of geographic and genetic separation between populations, and mutation rate could also influence the level of parallelism (Ord and Summers 2015; Kess et al. 2018), and recent theory suggests that nonrandom repeatability of evolutionary trajectories at the genomic level is likely due in part to low redundancy constraining possible paths to adaptation (Yeaman et al. 2018).

Although there are numerous examples of genetic parallelism or convergence for relatively simple traits (e.g., armor plating in sticklebacks [Jones et al. 2012], coat color in mice [Manceau et al. 2010], toxin resistance in snakes [McGlothlin et al. 2016]), less is known about the extent to which shared environmental pressures lead to similar architectures of adaptation for quantitative traits, which encompass the majority of ecologically relevant traits. Range shifts for plant species associated with past climate change have resulted in steep latitudinal and altitudinal gradients that provide natural experiments to test the degree to which parallel environmental clines lead to parallel phenotypic and genomic changes for quantitative, locally adaptive traits (Oubida et al. 2015; Holliday et al. 2016). Functional studies suggest that some of the same genes govern seasonal dormancy, a key adaptive trait, in both angiosperm and gymnosperm trees. For example, downregulation of *FLOWRERING LOCUS T* leads to bud set in both aspen and spruce trees, the timing of which varies with a tree’s latitudinal origins (Böhlenius et al. 2006; Gyllenstrand et al. 2007). Moreover, population genomic studies have documented parallel or convergent signatures of selection on additional genes involved in local climatic adaptation (Holliday et al. 2016; Yeaman et al. 2016). Despite efforts to understand repeated evolution within and between plant species, questions remain. Is the genetic basis underlying phenotypic adaptation conserved across lineages along parallel ecological clines? Do shared life history characteristics, ecological context, and selection regimes increase the likelihood of parallel genomic architecture of adaptation? Are the same environmental factors driving phenotypic and genetic adaptation?

*Populus trichocarpa* is a model tree species native to riparian areas of temperate western North America (Gornall and Guy 2007) and is widely distribution from southern Alaska to California. Patterns of variation across the species range for traits, such as bud phenology, biomass and growth, wood biochemistry, and lignin content, suggest that these traits are subject to spatially varying selection (Gornall and Guy 2007; Gailing et al. 2009; McKown, Guy, et al. 2014; Oubida et al. 2015; Wang et al. 2018). Although several studies of phenotypic and genomic adaptation across latitudinal gradients have been reported for *Populus* species, less attention has been paid to variation along altitudinal clines (Klepsatel et al. 2014; Halbritter et al. 2015). Altitude resembles latitude in forming a gradient of temperature and precipitation but may differ in other climate drivers such as frost, snowfall, evaporative demand, and solar radiation (Thomas 2011). A recent study in *P. trichocarpa* found a high level of shared loci under divergent selection across latitudinal and altitudinal clines, which is likely a result of parallel environmental selection (Oubida et al. 2015; Holliday et al. 2016). In this study, we aim to further understand parallel adaptation by investigating phenotypic adaptation and climatic selection among *P. trichocarpa* populations distributed across altitude and latitude. We combine genome-wide association analysis (GWAS) and genotype–environment association analysis to identify key loci underlying adaptive traits or targeted by climate selection in both transects and assess the extent of genetic parallelism across two parallel clines.

## Materials and Methods

### Plant Material and Exome Sequencing

**Fig. 1.**
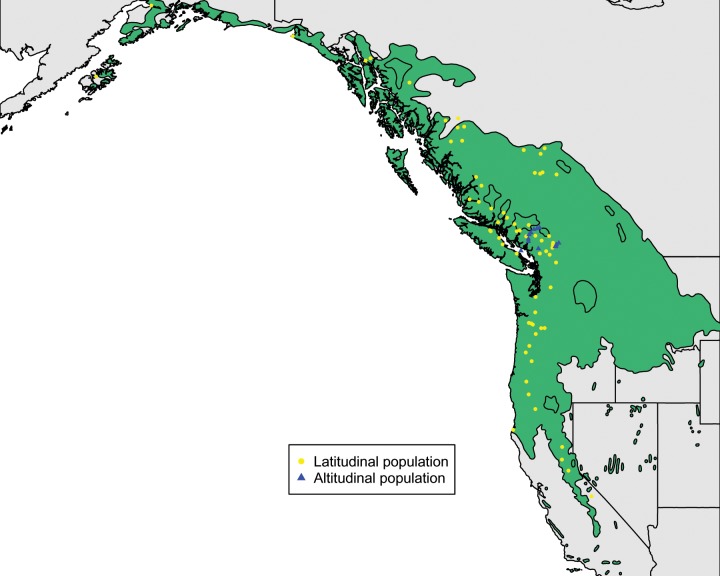
—Sample origin of 451 clones collected across the *Populus trichocarpa* species range (green shading). Yellow points indicate sampling locations along the latitudinal transect, and blue triangles indicate sampling locations of the altitudinal transect.

Branch cuttings representing 182 provenances spanning 20° of latitude (fig. 1) were rooted in a greenhouse and four replicates of each genotype were subsequently randomized to one of four blocks and planted in 2012 at two common gardens located at Critz, VA (36.63°N, 80.15°W) and Campbell River, British Columbia, Canada (50.06°N, 125.32°W). Genomic DNA was extracted from young leaves with the Qiagen DNeasy Plant Mini Kit (Qiagen, Inc, Valencia, CA). Oligonucleotide baits targeting the exome were designed using Agilent SureSelect eArray software (Agilent Technologies, Santa Clara, CA) based on the v2.0 reference *P. trichocarpa* genome (http://phytozome.jgi.doe.gov; last accessed July 25, 2019). Library preparation and target enrichment procedures were described previously (Zhou and Holliday 2012; Zhou et al. 2014; Holliday et al. 2016; Zhang et al. 2016). The captured libraries were sequenced on Illumina HiSeq 2000 System in paired end format (2 × 100) at the Virginia Bioinformatics Institute.

### Genotyping and Single-Nucleotide Polymorphism Calling

Following demultiplexing, trimming of adapter sequence, and removal of low quality reads (Zhou et al. 2014), reads were aligned to the *P. trichocarpa* genome with the BWA *sampe* function (Li and Durbin 2009). Indels were realigned using the GATK *IndelRealigner* function, and duplicate reads marked and removed with Picard *MarkDuplicates* and GATK *DuplicateReadFilter*, respectively (DePristo et al. 2011). Single nucleotide polymorphisms (SNPs) were called with the GATK HaplotypeCaller (https://www.broadinstitute.org/gatk/; last accessed July 25, 2019) (Poplin et al. 2018). Variants were flagged and removed as low quality if they had the following characteristics: low map quality (MQ < 40); high strand bias (FS > 40); differential map quality between reads supporting the reference and alternative alleles (MQRankSum < −12.5); bias between the reference and alternate alleles in the position of alleles within the reads (ReadPosRankSum < −8.0); and low depth of coverage (DP < 5). Missing genotypes on chromosomes 1–19 were imputed using BEAGLE software (Browning BL and Browning SR 2016).

### Population Structure and Spatial Grouping

We divided the poplar clones into two spatial transects: A latitudinal transect consisting of 68 provenances spanning the latitudinal species range (285 genotypes), and an altitudinal transect composed of 13 provenances distributed across the Coquihalla Highway (49.75°N, 121.00°W; 63 genotypes) and Highway 99 (50.08°N, 123.06°W; 103 genotypes) in southwest British Columbia (fig. 1). We divided the 285 latitudinal samples into six broad geographic areas (Alaska, California, Oregon, Washington, North British Columbia, Interior British Columbia, and South British Columbia) and the 166 altitudinal samples into three elevational bands (elevation < 300 m = low, 300–800 m = middle, and >800 m = high). To visualize overall population structure across all samples, we randomly selected 10,000 SNPs with linkage disequilibrium ($r^{2}$) < 0.2 to perform principal component analysis using the *prcomp* function in R (R Core Team 2015) for latitudinal and altitudinal samples (supplementary fig. S1, Supplementary Material online).

### Phenotyping of Climate-Related Traits

Height and diameter were measured after all trees set bud in the fall of 2013. The stages of bud set and bud flush were scored weekly until most trees formed terminal bud or until all trees had a fully expanded leaf, respectively (Frewen et al. 2000; Rohde 2002). Bud set timing was calculated as the days elapsed for trees to reach a fully developed bud from January 1, whereas timing of bud flush was expressed as the number of days to the first fully unfolded leaf. Cold hardiness was measured in 2012 by sectioning lateral shoots into ∼0.5-cm discs and treated samples with temperature of −8, −14, and −20 °C (starting temperature: 4 °C; decreasing rate 4 °C/h) for 1 h before transferring them back to 4 °C to thaw. Control samples were maintained at 4 °C throughout the experiment. The electrolytic conductivity of the solution was measured and cold injury index calculated after Hannerz et al. (1999), averaged across temperatures for each clone. Finally, after coppicing trees in the Virgina garden in May 2014, we recorded the average height of the regenerated main branches and the number of regenerated branches in March 2015.

### Phenotypic Best Linear Unbiased Predictors

We estimated clonal best linear unbiased predictors (BLUPs) for each trait with a linear mixed model using the *lmer* function in *lmerTest* package in R (Kuznetsova et al. 2017) as follows:

$$y_{\mathrm{ijk}}=\mu +b_{i}+c_{j}+{ɛ}_{\mathrm{ijk}},$$

where $y_{\mathrm{ijk}}$ is the phenotype of the *k*th replicate of *j*th clone in *i*th block, μ is the grand mean, $b_{i}\;$is the random effect of *i*th block, $c_{j}$ is the random effect of *j*th clone, and ${ɛ}_{\mathrm{ijk}}$ is the random error following $N(0,\;\sigma_{ɛ}^{2}I)$, where $\sigma_{ɛ}^{2}$ is variance and I is the identity matrix. The resulting BLUPs were used as phenotypes in the following GWAS.

### Association Analysis of Climate-Related Traits

To control for population structure in our association analyses, we evaluated the fit of several alternative models, namely a simple model (no population structure controlled), models with principal components as covariates (2, 5, and 10 of the leading principal components as covariates), models with familial kinship controlled (*K*; estimated in TASSEL [Bradbury et al. 2007], and the combined model (2PC + *K*), and selected the best structured models using the genomic inflation factor $\lambda_{\mathrm{GC}}\;$and QQ plots (supplementary table S1 and supplementary fig. S2, Supplementary Material online). With dense SNP coverage and linkage among SNPs, as well as polygenic genetic inheritance underlying the phenotypic traits, the false positive rate is expected to increase (Yang et al. 2011; McKown, Klápště, et al. 2014). As a compromise between false positives and false negatives, we selected the most parsimonious model with a genomic inflation factor ($\lambda_{\mathrm{GC}})\;$of at least 0.98 but not more than 1.22.

The GLM (generalized linear model) models were as follows:

y=Sα+Qν+ɛ,

where y is a vector of phenotypic observations, α is a vector of SNP effects, ν is a covariate of population structure (PC) effects, and ε is residuals following $N(0,\;\sigma_{\varepsilon }^{2}I)$. *S* and *Q* are incidence matrices of 1s and 0s relating y to α, ν, respectively. The MLMs (mixed effects linear models) were fitted as follows:

y=Xβ+Zu+ɛ,

where y is a vector of observed phenotype, β is a vector containing coefficients of the fixed effects (SNP effects and population structure effects), u is a vector of random additive effect for individuals with $\mathrm{Var}\left(u\right)=\sigma_{g}^{2}K$, where K is the kinship matrix. X and Z are incidence matrices mapping y to β and u. ɛ is the random residual effect following $N(0,\;\sigma_{ɛ}^{2}I)$ (Bradbury et al. 2007).

### Identification of Top Candidate Genes

Because natural selection is likely to lead to elevated LD across relevant genes or regulatory regions, we performed a gene-based analysis of SNP–phenotype associations to identify candidate genes or genomic regions underlying adaptive traits for both transects. We first annotated all SNPs to genes that contained them or to genes within 2 kb based on the *P. trichocarpa* v3.0 genome annotation. We then collapsed intergenic regions into 5-kb clusters to capture SNPs that fall outside the neighborhood of any gene. This resulted in binning of the 1.3 million SNPs into 42,970 genes or intergenic regions. For each trait, we identified SNPs in the first percentile of *P* values from the selected model as outliers. The number of SNP outliers and total number of SNPs were counted for each gene or intergenic region, and classified as top candidate genes when their proportions of significant SNPs exceeded the 0.999 quantile of the binomial expectation (Yeaman et al. 2016). The expected proportion of outliers per gene was defined as the mean proportion of SNP outliers across genes with at least five SNPs in total and with at least one SNP outlier. Finally, we assigned gene-wise *P* values for each gene or intergenic region based on a binomial distribution (Yeaman et al. 2016). This gene-based method has more power to detect genomic clusters of loci possessing pronounced association signals that exceed the genomic background and reduces false positives caused by isolated outliers (Liu et al. 2010; Kang et al. 2013).

### Test of Parallel Evolution across Two Transects

The null-*W* test of convergence, first proposed by Yeaman et al. (2016), is a more powerful test for uncovering signatures of convergence, which may not be detected by comparing direct overlap in gene outliers. The null-*W* test adjusts for factors such as gene size and linkage that may influence the test’s significance by comparing candidate and noncandidate genes to a SNP control panel (Yeaman et al. 2016). For each candidate gene in one transect, this test evaluates the association strength of the same gene in the other transect by comparing it to a null distribution. To construct the null distribution, we extracted the $R^{2}$ of a random sample of 10,000 SNPs from noncandidate genes (i.e., those not identified as “top candidates”) as the control panel, and the $R^{2}$ of all SNPs annotated to noncandidate genes. We then calculated the test statistic *W* with a Wilcoxon-signed rank test by comparing SNP $R^{2}$’s of each noncandidate genes versus that of the SNP control panel, using the R function “wilcox.test.” These 10,000 null-*W* test statistics were converted into a null distribution of Z scores using $Z=\left(2W-n_{1}n_{2}\right)/\sqrt{n_{1}n_{2}(n_{1}+n_{2}+1)/3}$, where $n_{1}$ and $n_{2}$ are the number of SNPs in the SNP control panel and noncandidate genes (Whitlock and Schluter 2009; Yeaman et al. 2016). Finally, the $R^{2}$ of SNPs annotated to candidate genes were compared with the 10k control panel their *W* test statistic calculated and converted into *Z* scores. The *P* value for the null-*W* test was calculated as the number of genes with *Z* score exceeding the observed *Z* score in the null distribution divided by the total number of genes in the null distribution. The empirical *P* values were further adjusted using the false discovery rate (Benjamini and Hochberg 1995) and candidate genes with adjusted *P* value ≤ 0.05 were considered as parallel outliers.

### Genotype–Environment Association Analysis

Climate variables were estimated for each sampling site with ClimateWNA software (Wang, Hamann, et al. 2012) based on averages for the period 1961–1990. Annual climate variables included MAT, MWMT, MCMT, MWMT, MAP, MSP, AHM, and SHM. Derived variables included DD_0, DD5, DD_18, DD18, NFFD, FFP, bFFP, eFFP, PAS, EMT, EXT, Eref, and CMD (supplementary table S2, Supplementary Material online). We used Bayenv2 (Gunther and Coop 2013) to test for univariate associations between allele frequency and the 21 environment variables, as well as latitude, altitude, and elevation. We first estimated the population covariance matrix based on a set of 10,000 intergenic SNPs, and then tested for correlations between allele frequencies and the climatic variables using 100,000 iterations, averaged across three runs. For each climate variable, we classified SNPs with average log_10_(BF) > 2 as selection outliers. We also performed a gene-based analysis as above to identify candidate genes with excessive number of climate-associated SNPs, and null-*W* tests for parallel adaptation were performed on top candidate genes identified in each transect. The squared spearman correlation coefficients were used in constructing the null distribution and the null-*W* test.

We also used redundancy analysis (RDA), a multivariate constrained ordination method, to test for gene–environment relationships (Forester et al. 2016, 2018). RDA fits multivariate linear regressions between genotype and environment to obtain a matrix of fitted values, and then uses principal component analysis on the fitted values to produce ordination axes. To reduce the multicollinearity among climate variables, we estimated correlations among them and retained seven representative and relatively uncorrelated variables (MAT, MWMT, TD, MAP, AHM, SHM, FFP, PAS, EMT, EXT, and Eref). For each of the first three axes, SNPs with a “locus score” exceeding the mean ± 3 SD were considered outliers associated with their most highly correlated climate variable. All RDA calculations were performed with the R package “vegan” (Forester et al. 2018).

### Annotation of Top Candidate Genes

To identify overrepresented biological processes or molecular functions among top candidate genes identified for each trait, we first performed gene ontology (GO) enrichment analysis with R package topGO. Genes with at least five SNPs and at least one SNP outliers were used as background sets for each trait and each transect. The parent–child method is employed in the GO enrichment analysis to account for the dependency structure among GO terms. Only GO terms associated with at least five genes were tested for GO enrichment and those with *P* value < 0.05 were considered as interesting biological processes. The same GO enrichment analysis procedure was performed on parallel adaptation outliers with all candidate genes as the background gene set.

## Results

### Clinal Variation of Phenotypic Traits and Climatic Variables

**Fig. 2.**
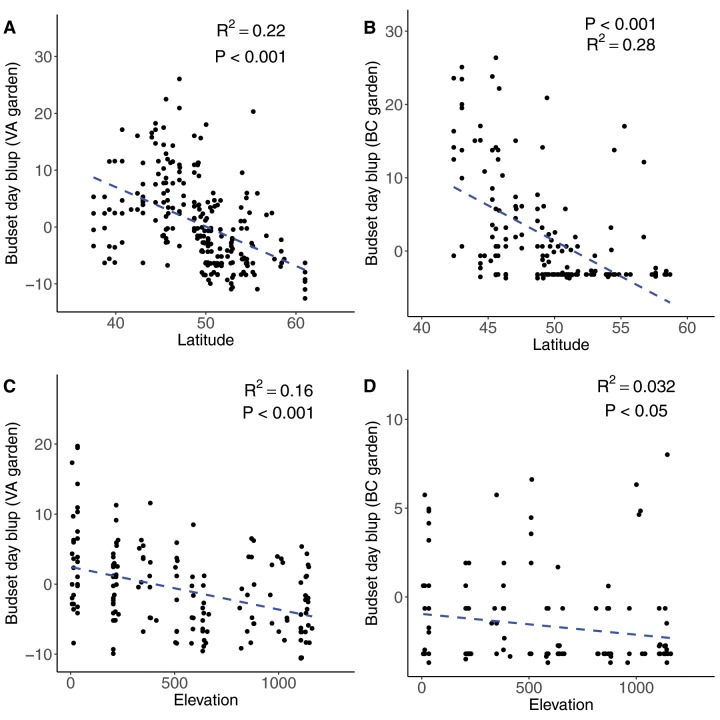
—Phenotype BLUPs in relation to latitude and elevation among latitudinal and altitudinal samples. Phenotypic traits include timing of bud set in VA garden (*A*, *C*) and timing of bud set in BC garden (*B*, *D*).

We observed moderate to strong clinal variation among traits along the latitudinal and altitudinal transects (fig. 2; supplementary figs. S3 and S4, Supplementary Material online). Latitude and elevation showed similar patterns for timing of bud phenology, cold hardiness, and coppice regeneration. As latitude or altitude increased, the date of bud set and cold injury significantly decreased, whereas the date of bud flush increased (fig. 2; supplementary figs. S3 and S4, Supplementary Material online). Plant height, diameter, regeneration height, and regenerated branch number significantly decreased as altitude or clone distance to range center increased (supplementary figs. S3 and S4, Supplementary Material online). The correlation pattern between traits and latitude/elevation was consistent in direction for both Virginia and British Columbia common gardens despite some differences in strength (fig. 2; supplementary figs. S3 and S4, Supplementary Material online).

Most phenotypic traits showed significant covariation with climate and geographical variables across both transects (supplementary fig. S5 and supplementary table S3, Supplementary Material online). Among latitudinal samples, there was a positive correlation between the timing of bud set and temperature-related variables (MAT, MWMT, MCMT, DD5, and FFP), whereas bud flush showed a negative relationship with the same set of variables (supplementary table S3, Supplementary Material online). Height and diameter both showed positive relationships with MAT, MWMT, MCMT, DD5, FFP, and Eref, and negative relationships with TD, MAP, and DD_0 (supplementary table S3, Supplementary Material online). Cold injury, regeneration height, and regeneration branch number had the same correlation direction with all climatic variables as height and diameter but different strengths (supplementary table S3, Supplementary Material online). Most phenotypic traits along the altitudinal transect displayed the same correlation direction as for latitude, but these correlations were weaker with climatic variables compared with latitudinal samples (supplementary table S3, Supplementary Material online). The relationship between latitude and climate was driven by temperature (MAT, MWT, MCMT, and TD), moisture (MSP, AHM, SHM, and Eref), and degree-days (DD_0, DD5, and FFP) (supplementary table S3, Supplementary Material online), whereas temperature (MAT, MWT, MCMT, and TD) and degree-days (DD_0, DD5, and FFP) were most strongly correlated with altitudinal variation.

### Phenotype–Genotype Associations across Two Transects

**Fig. 3.**
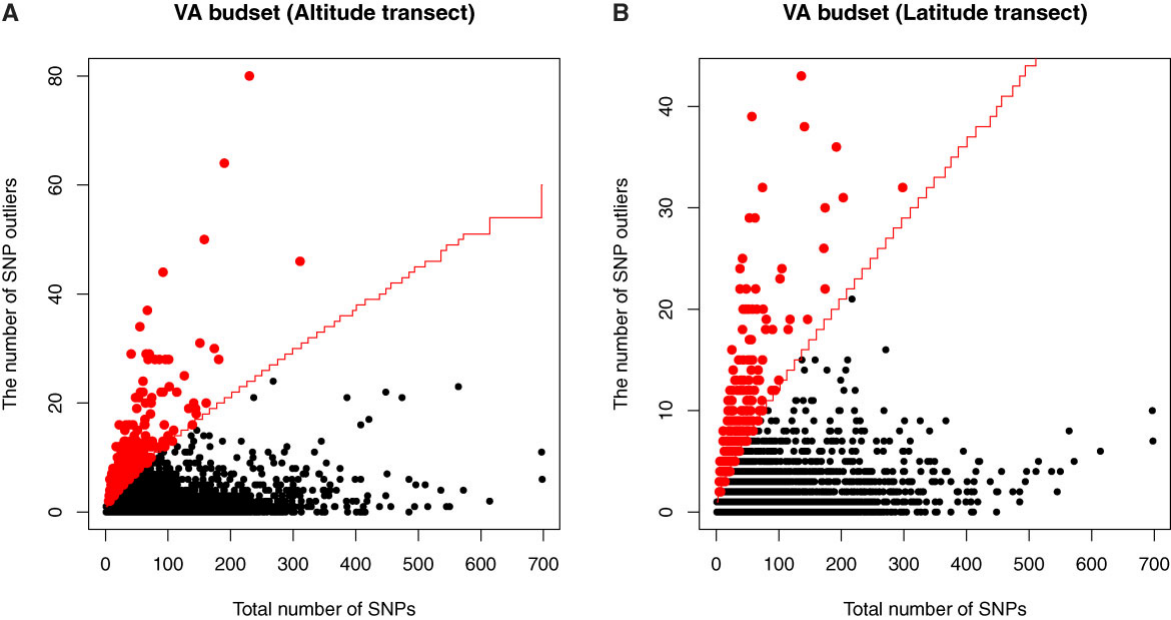
—Gene-based analysis to identify top candidate genes associated with timing of bud set in the Virginia garden among altitudinal samples (*A*) and latitudinal samples (*B*). Red line indicates the expected number of SNP outliers based on 99.9% binomial quantiles given the total number of SNPs within each gene or genomic region. Red points are candidate genes or intergenic regions with an enriched number of SNP outliers.

**Fig. 4.**
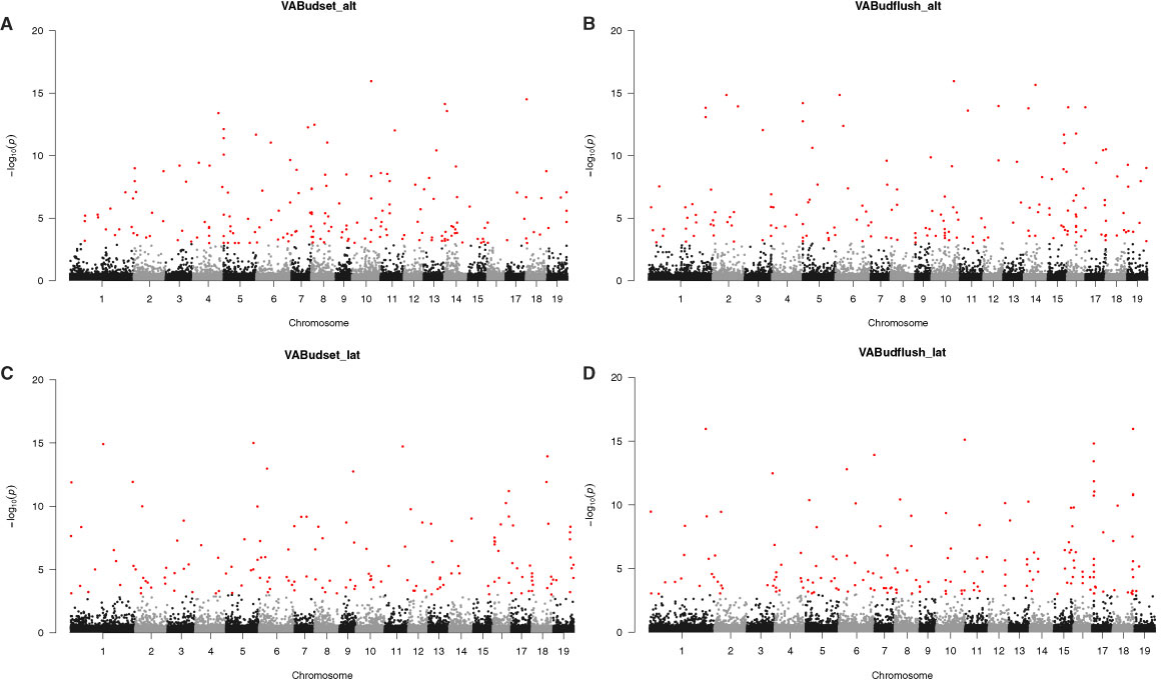
—Manhattan plot of gene-wise *P* values from association analysis of timing of bud set (*A*, *C*) and bud flush (*B*, *D*) at the Virginia (VA) common garden in altitudinal (alt) and latitudinal (lat) samples.

After filtering, we obtained 1,311,373 SNPs with <25% missing data per SNP and minor allele frequency (MAF) > 0.05. Of these, 1,215,483 SNPs were mapped to chromosomes 1–19 and 95,890 SNPs to other scaffolds. We identified candidate genes for each phenotypic trait as those genes or intergenic regions harboring excessive number of SNP outliers above the 0.999 binomial quantile (fig. 3; supplementary figs. S6 and S7, Supplementary Material online). To visualize these candidates, we plotted their gene-wise *P* values in relation to their physical location in the poplar genome (fig. 4; supplementary figs. S8 and S9, Supplementary Material online). Among altitudinal samples, we detected 333 unique candidate genes for plant height, 353 for timing of bud set, 318 for timing of bud flush, 193 for diameter, 194 for regeneration height, 197 for regeneration branch number, and 172 for cold hardiness. A comparable number of candidate genes were identified for the latitudinal transect (table 1). Annotations of the top candidate genes along the altitude transect suggest a variety of biological processes involved. For bud phenology, these included glycosyl compound metabolic process (GO:1901657), transmembrane transport (GO:0055085), and cellular response to stress (GO:0033554) (supplementary table S4, Supplementary Material online). Across latitude, bud phenology candidates were enriched for small molecule metabolic process (GO:0044281), response to cold (GO:0009409), cellular response to stress (GO:0033554), and lipid transport (GO:0006869) (supplementary table S5, Supplementary Material online). Height-associated candidate genes were overrepresented in developmental process including cellular localization process (GO:0051641), catabolic process (GO:0009056), and cell wall organization or biogenesis (GO:0071554) across the altitude transect (supplementary table S4, Supplementary Material online), whereas for latitude, carbohydrate metabolic process (GO:0044723), carbohydrate transport (GO:0008643), and regulation of cellular component size (GO:0032535) were overrepresented (supplementary table S5, Supplementary Material online).

**Table 1:** Number of Candidate Genes and Parallel Outliers for the Altitudinal and Latitudinal Groups

Traits and Climate Variables	Altitude transect		Latitude transect		Direct Overlap	*P Value*
	Candidate Genes	Parallel Genes (Null-*W*)	Candidate Genes	Parallel Genes (Null-*W*)		
Height_VA	170	0	186	0	2	0.168
Bud set_VA	197	0	175	0	4	0.0088
Bud flush_VA	185	0	187	0	6	0.0002
Diameter_VA	193	0	167	0	3	0.0398
Regen_height_VA	194	0	143	0	2	0.1367
Regen_branch_VA	197	0	161	0	2	0.1688
Cold_VA	172	0	191	0	0	—
Height_BC	168	0	187	0	0	—
Bud set_BC	156	0	156	0	1	0.4336
Bud flush_BC	134	0	145	0	0	—
Latitude	233	0	16	2	2	0.003
Longitude	96	0	5	0	0	—
Elevation	201	0	32	0	2	0.01
MAT	192	0	35	0	2	0.011
MWMT	189	0	19	0	0	—
MCMT	215	0	45	0	5	3.11E-06
TD	216	0	50	12	9	3.63E-12
MAP	77	0	110	0	4	4.76E-05
MSP	26	0	36	0	0	—
AHM	86	0	98	3	2	0.017
SHM	76	0	57	7	1	0.096
DD_0	190	0	62	0	5	8.46E-06
DD5	192	0	25	0	0	—
DD_18	197	0	41	2	2	0.015
DD18	178	0	20	1	2	0.003
NFFD	204	0	49	0	2	0.023
bFFP	210	0	36	0	0	—
eFFP	218	0	63	3	5	1.78E-05
FFP	212	0	45	6	0	—
PAS	93	6	17	0	0	—
EMT	199	0	50	0	2	0.023
EXT	275	0	28	0	3	0.001
Eref	245	0	20	0	1	0.108
CMD	72	3	53	0	1	0.085

Note.—The first four columns show the number of gene-based outliers from the binomial distribution and respective number of these identified as parallel outliers by the null-*W* test. The final two columns show number of candidate genes exceeding the 99.9th percentile of the binomial distribution in both transects, and *P* values estimated using a hypergeometric test.

**Fig. 5.**
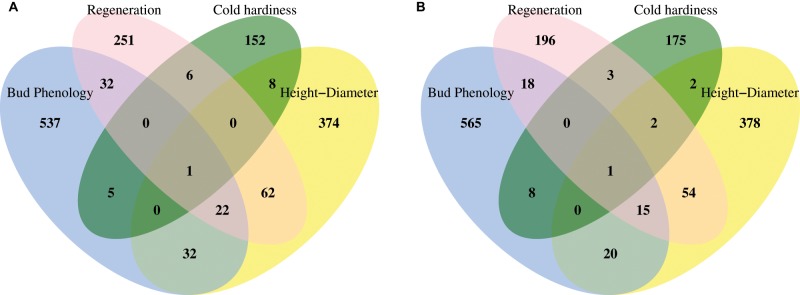
—Venn diagram of candidate genes associated with phenotypic traits across the altitudinal (*A*) and latitudinal transects (*B*). Bud phenology refers to candidate genes associated with timing of bud set and/or bud flush. Regeneration refers to candidate genes associated with regeneration height and/or regenerated branch number. Height–diameter refers to candidate genes associated with height and/or diameter.

A few genomic regions uncovered by the gene-based analysis showed association with multiple traits in both transects (fig. 5). For example, genes associated with both timing of bud flush and bud set for the altitude transect included Potri.001G349900 (leucine-rich repeat protein kinase family protein), Potri.002G056300 (PSB27; photosystem II family protein), and Potri.014G134900 (NHX2; sodium hydrogen exchanger 2). Potri.014G132800 (LD; LUMINIDEPENDENS) and Potri.008G166600 (CRY2; Cryptochrome 2) were associated with both bud phenology and diameter. Across the latitude transect, Potri.017G033100 (GPX3; glutathione peroxidase 3) was associated with timing of bud flush, cold hardiness, height, and regeneration height. Candidate genes, such as Potri.003G131300 (BEL1; BELL 1), Potri.006G277500 (CRY3; Cryptochrome 3), Potri.017G016200 (Homeodomain-like transcriptional regulator), were associated with both timing of bud flush and bud set.

### Parallel Phenotype–Genotype Associations between Spatial Transects

Using the null-*W* test, between 3% and 10% of candidate genes showed signatures of parallel association for both transects (table 1). Genes associated with timing of bud set and bud flush had the most significant overlap across two transect (*P* < 1.0e^−4^ in all hypergeometric tests). Among shared gene outliers, Potri.016G088300 (NAM; No apical meristem) and Potri.016G088800 (transducin family protein) were associated with timing of bud break in both transects, whereas Potri.001G464700 (FAD-binding Berberine family protein) was associated with plant height and diameter in both transects (supplementary table S6, Supplementary Material online).

### Genotype–Environment Associations across Two Transects

**Fig. 6.**
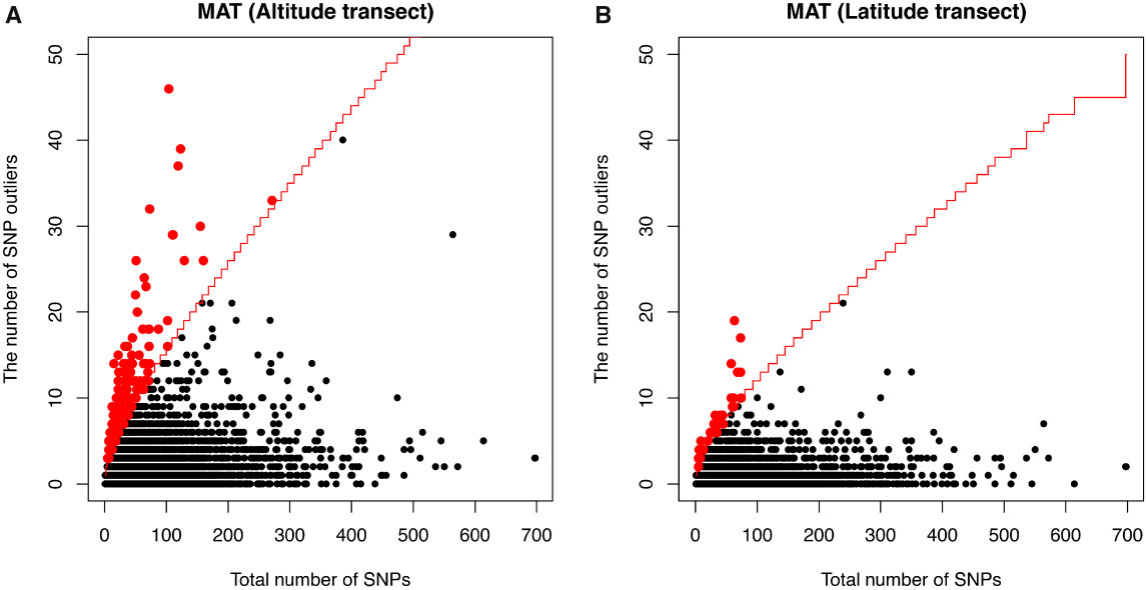
—Gene-based analysis to identify top candidate genes associated with MAT among altitudinal (*A*) and latitudinal samples (*B*). Red line is the expected number of SNP outliers based on 99.9% binomial quantile given total number of SNPs within each gene or genomic region, and red points are candidate genes or intergenic regions enriched for SNP outliers.

Bayenv2 analysis suggested a number of candidate genes or genomic regions strongly correlated with one or more of the 21 climate variables and 3 geographic variables (fig. 6; supplementary figs. S10 and S11, Supplementary Material online; table 1) for either the latitude or the altitude transect. Among genes associated with mean annual temperature (MAT), GO terms including cellular response to stress and carbohydrate metabolic process were enriched across altitude, and steroid biosynthesis and response to cold across latitude (supplementary tables S7 and S8, Supplementary Material online). More than 40% of candidates associated with MWMT and MCMT were also associated with MAT across both transects. Candidate genes associated with these temperature variables across altitude included Potri.001G261300 (LRR and NB-ARC domains-containing disease resistance protein), Potri.001G444700 (NB-ARC domain-containing disease resistance protein), and Potri.015G110100 (FRI; FRIGIDA-like protein).

Candidate genes associated with mean annual precipitation (MAP) were mostly involved in cellular component organization, histone modification, and establishment of protein localization (table 1; supplementary tables S7 and S8, Supplementary Material online). The MAP-associated genes had <4% overlap with temperature-related climate variables across both transects. Heat-moisture related genes were enriched in response to abiotic stimulus and polysaccharide biosynthetic process across altitude, and cellular aldehyde metabolic process and response to endogenous stimulus across latitude (supplementary tables S7 and S8, Supplementary Material online). For altitude, one SHM-associated candidate gene—Potri.014G170600 (COL9; CONSTANS-like 9)—may downregulate expression of CONSTANS and FT and control bud dormancy in poplar (Böhlenius et al. 2006). In general, pathways including cellular response to stress (GO:0033554), lipid binding (GO:0008289), response to cold (GO:0009409) were overrepresented among candidate genes associated with most climate variables (supplementary tables S7 and S8, Supplementary Material online).

### Parallelism in Climate-Selected Loci between Transects

We detected a significant number of candidates that directly overlapped based on the binomial test in each transect. These were associated with MCMT, TD, MAP, DD_0, and eFFP (table 1; *P* < 1.0e^−4^ based on a hypergeometric test). Additionally, the Null-*W* test revealed additional candidate genes with signatures of climatic selection in both transects (supplementary tables S9 and S10, Supplementary Material online). For example, nine outliers in the altitude transect were significant based on the null-*W* test in the latitude transect, including Potri.011G136100 (DAWDLE; DDL), which was associated with AHM, MSP, PAS, and SHM. This gene encodes a protein similar to DAWDLE, and its ortholog in *Arabidopsis* is involved in biogenesis of miRNAs (Zhang et al. 2018). Among climate-associated genes identified in the latitude transect, 27 displayed parallel association with the same climate variable across altitude. One gene Potri.001G282300 (UGT87A2; UDP-GLUCOSYL TRANSFERASE 87A2) associated with mean monthly temperature difference (TD), encodes a putative glycosyltransferase, which regulates abiotic stress response and flowering time via FLC (FLOWERING LOCUS C) in *Arabidopsis* (Wang, Jin, et al. 2012; Li et al. 2017).

### Multilocus Genotype–Climate Association

**Fig. 7.**
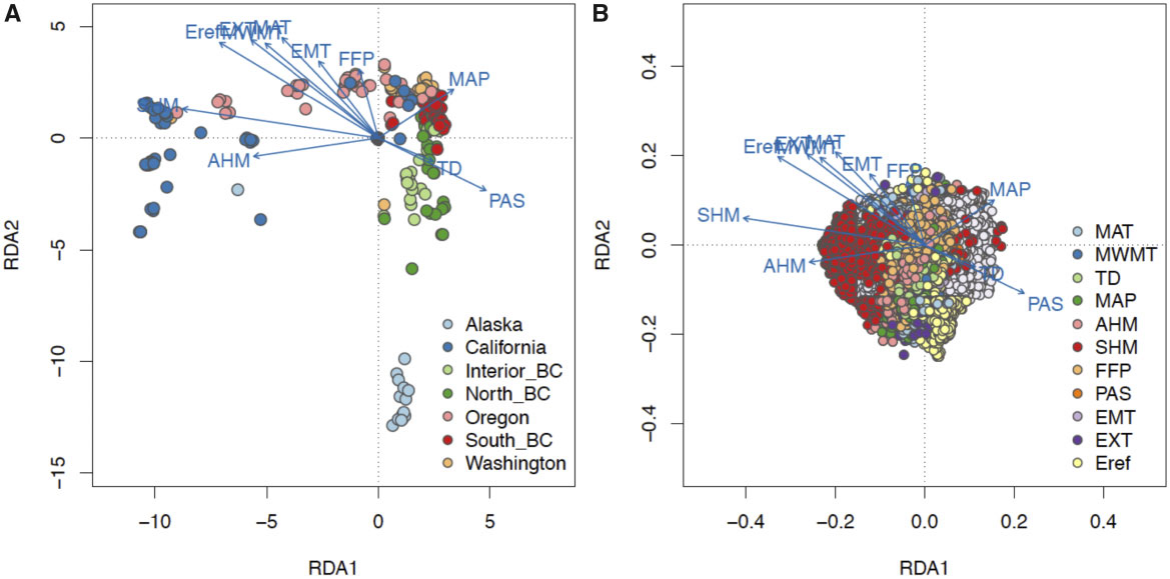
—Triplots of six latitudinal groups (*A*) and SNP loadings (*B*) on RDA axis 1 and axis 2 along the latitudinal transect. Points in (*A*) represent 285 poplar clones colored by geography, and points in (*B*) represent candidate SNPs colored by their most highly correlated environmental predictor. Blue vectors represent environmental predictors.

**Fig. 8.**
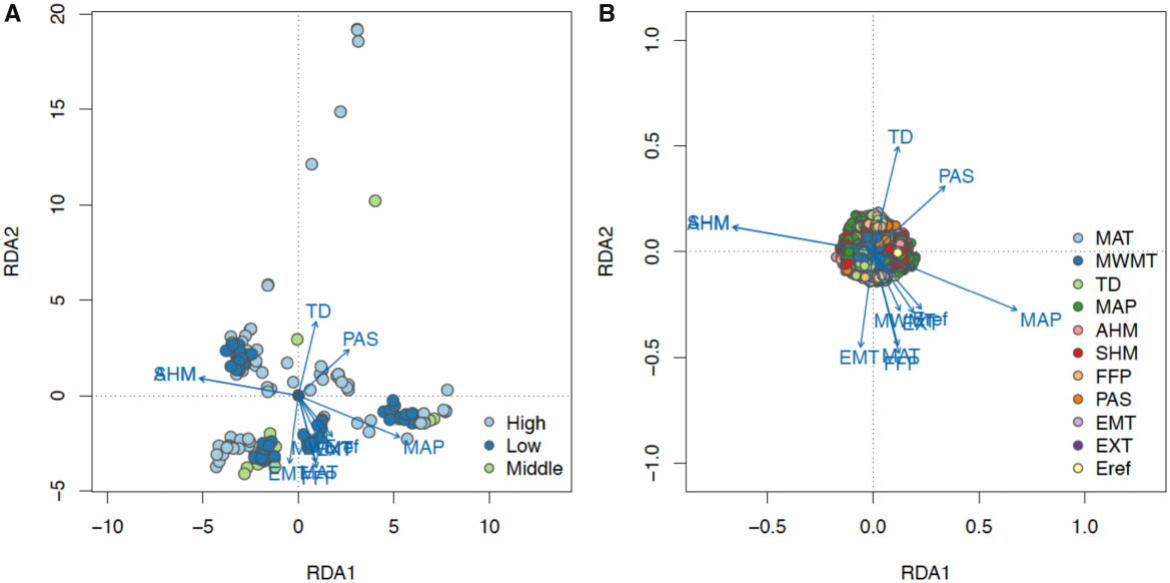
—Triplots of three elevation groups (*A*) and SNP loadings (*B*) on RDA axis 1 and axis 2 along the altitudinal transect. Points in (*A*) represent 166 poplar clones colored by geography, and points in (*B*) represent candidate SNPs colored by their most highly correlated environmental predictor. Blue vectors represent environmental predictors.

Using RDA, we identified the major climate agents driving the formation of ecotypes among latitudinal and altitudinal samples. Across the latitude transect, California and Oregon sampling locations were positively related to high MAT, high heat-moisture, and growth period (positive MAT, MWMT, AHM, SHM, and FFP); Alasak sites were characterized by low annual temperature and frost free period; northern and interior British Columbia were correlated with high precipitation as snow and continentality (positive TD, PAS, and negative SHM, AHM); and Washington and southern British Columbia were positively correlated with high MAP (fig. 7*A*). For the altitude transect, high elevation sites were positively related to precipitation as snow and continentality, similar to Alaska (fig. 8*A*). In contrast, middle and low elevations had higher MAT, MAP, and heat-moisture.

On the first three RDA axes, we identified 16,621 candidate SNPs across altitude and 22,693 across latitude (figs. 7*B* and 8*B*). In general, SNP outliers on altitude axis one reflected associations with heat-moisture and precipitation; outliers on axis two reflected continentality, extreme temperature and frost free period; and outliers on axis three reflected temperature (MWMT, MAT) and precipitation as snow (fig. 8*B* and supplementary fig. S12, Supplementary Material online). Across latitude, candidate SNPs on axis one reflected associations with heat-moisture, MWMT, and Eref; SNPs on axis two were associated with temperature and precipitation; and SNPs on axis three were associated with TD and FFP (fig. 7*B* and supplementary fig. S12, Supplementary Material online).

Among SNP outliers detected across altitude, MAT, MWMT, MAP, and TD had the highest number of candidate loci. For latitude, MAT, SHM, FFP, and Eref had the most correlated candidate SNPs. There were 2,710 overlapping SNP outliers found in both transects, among which 24 were identified based on the same environmental predictor in both transects. One SNP associated with SHM in both transect was located in an intron of Potri.002G180800 (LATE ELONGATED HYPOCOTYL; LHY), which encodes a MYB-related protein regulating circadian clock by interacting with CCA1 in *Arabidopsis* (Kim et al. 2003). In another case, four outlier SNPs strongly associated SHM across latitude were also correlated with MAT, FFP, EMT, and PAS across altitude. These four SNPs are located on chromosome 10 within the introns of gene Potri.010G179700, which encodes flowering regulator FT2 (FLOWERING LOCUS T). This gene was previously found regulating growth and dormancy cycling in poplar (Hsu et al. 2011; Wang et al. 2018).

### Overlapping Signals between Genotype–Phenotype and Genotype–Climate Associations

There was a significant overlap between gene outliers from GWAS and Bayenv2 for both altitude (5.67% of GWAS and 8.85% of Bayenv were in common; *P* = 4.91e^−15^ based on a hypergeometric test) and latitude (2.78% of GWAS and 9.73% of Bayenv outliers were in common; *P* = 2.16e^−9^ based on hypergeometric test) (supplementary fig. S13, Supplementary Material online). We also observed substantial overlap between SNP outliers detected with the two genotype–environment association analysis approaches. Among candidate SNPs identified by RDA, 47.3% were in common with Bayenv2 results for altitude and 55.0% for latitude. Among the eight selected climate predictors, MAT and MAP had the most significant overlap in SNP outliers detected in both Bayenv2 and RDA across both transects (*P* < 2.53e^−5^ in all hypergeometric tests; supplementary table S11, Supplementary Material online).

## Discussion

### Phenotypic Variation Reflects Adaptation to Climate Regimes

Our results support previous findings of adaptive phenotypic gradients among natural populations along the latitudinal species range of *P. trichocarpa* (Evans et al. 2014; McKown, Guy, et al. 2014), and we further demonstrated that clines related to altitudinal adaptation also drive phenotypic differentiation. The strong phenotypic differentiation among natural accessions of *P. trichocarpa* along altitudinal and latitudinal gradients suggests that divergent selection related to local climate shapes phenotypic variation in the seasonal growth, cold hardiness, and regeneration ability of local populations across both coarse and fine spatial scales. Among the traits we measured, timing of bud phenology is a key adaptive response to seasonal variation in temperature. Provenances from high latitudes usually require a longer critical day length to induce bud formation and thus have earlier growth cessation and timing of bud set (Hurme et al. 1997; Rohde et al. 2011). In spring, bud break requires autumn chilling between 0 and 5 °C, followed by sufficient heat-sum (Campbell and Sugano 1975; Hunter and Lechowicz 1992; Li et al. 2010). The late bud flush of northern and high elevational genotypes in our study may in part be due to an unsatisfied chilling or heat-sum requirements (Li et al. 2010; Basler and Korner 2014). With shorter growing seasons after transfer to nonlocal environment, most northern and high elevation genotypes displayed less growth than those originating from the center of the species range or low elevation. Although development and release of cold hardiness depends in part on daylength, it is also controlled by temperature. The spatially varying freezing tolerance we observed also reveals adaptation to different temperature regimes. Finally, regeneration ability is an important fitness measure for species that reproduce partially through clonality (Martínková and Klimešová 2016), but less is known about the molecular determinants of these traits. The clinal pattern of regeneration ability may reflect adaptive differentiation in regeneration potential (Ikeuchi et al. 2016).

### Phenotype-Associated Genes Conserved across Geographic Transects

We uncovered numerous loci with annotations suggestive of their involvement in timing of bud phenology, height, diameter, regeneration height, and branch number. The gene-based approach we employed captured genomic regions harboring concentrations of significant SNP associations in excess of the genomic background. Compared with traditional GWAS, gene-based analysis aims to identify clusters of SNP outliers and thus is less prone to false positives. Annotations of some candidate loci suggested their possible roles in controlling the corresponding phenotypes. For example, Potri.015G107000 (Regulator of chromosome condensation [RCC1] family protein), which was associated with timing of bud set, may be involved in cold acclimation (Ji et al. 2015). Another gene associated with timing of bud flush, Potri.016G088300 (No apical meristem protein; NAM) encodes a key regulator in shoot apical meristem determination (Souer et al. 1996).

By assessing the direct overlap between candidate genes, we observed common phenotype-associated genes shared by the two transects. Among them, genes associated with timing of bud set and bud flush in Virgina garden showed the most overlap in the hypergeometric test. The null-*W* test results provided limited evidence of shared genetic architecture underlying phenotypic adaptation to our two geographic clines. This lack of parallelism may due to divergent selection forces targeting different genes or genomic regions between latitude and altitude. However, we cannot rule out the possibility that limited sample size and control for population structure may misclassify some true positives and reduce the power of the null-*W* test.

### Parallel and Discordant Patterns of Climate-Related Selection

The latitude and altitude transects we studied represent similar environmental clines that vary in the type and amount of precipitation, mean and seasonal temperatures, and yearly timing of growing seasons. One major shared selection agent across these two transect is local differences in MAT. Northern genotypes and those from high altitude are adapted to lower MAT and colder winters. Consequently, we expect the genomes of local populations that experience similar temperature regimes to respond in parallel, to the extent that genetic constraints exist for the loci of adaptation. To detect these signatures, we performed Bayenv2 and RDA scans and discovered a significant level of shared outlier SNPs and genes between the two transects, which is unlikely to have occurred by chance. This suggests that local selection may target similar standing genetic variations and drive adaptive allele frequency shifts across spatial groups. The GO enrichment analysis also pointed out three key pathways—carbohydrate metabolic process, cellular component organization, and lipid binding—enriched across both transects. The climate variables that showed the greatest degree of overlap between transects were related to temperature and precipitation, whereas genotype–environment relationships for variables related to evaporative demand (i.e., the interaction between temperature and precipitation in summer) did not significantly overlap. This dichotomy likely reflects the strong gradient in evaporative demand across latitude, and the lack of such a gradient across the altitudinal transect.

Despite the parallel selection signals, we also observed a large number of discordant selection outliers between the latitude and altitude transects. This is likely in part due to selective constraints unique to each transect favoring different genetic variants. For example, atmospheric pressure and daily temperature difference tend to influence plant physiology and metabolism across altitudinal gradients, whereas day length and solar angle of incidence vary across latitudinal gradients (Altshuler and Dudley 2006). The RDA revealed that temperature-related variables and yearly temperature difference are key selective forces driving population differentiation across our altitude transect, whereas temperature, heat-moisture, and seasonal growth period are the prominent selection factors for latitude. With climate gradients of varied scale across the two transects, the association strength of causal genes and pathways differ correspondingly. At the same time, historical distributions and complex demographic processes specific to local populations likely also contribute to the lack of concordance between two transects (Renaut et al. 2014; Holliday et al. 2016). Future analysis should explore how genomic features and population history may limit parallelism.

### Convergence and Discordance between Selection Scans

**Fig. 9.**
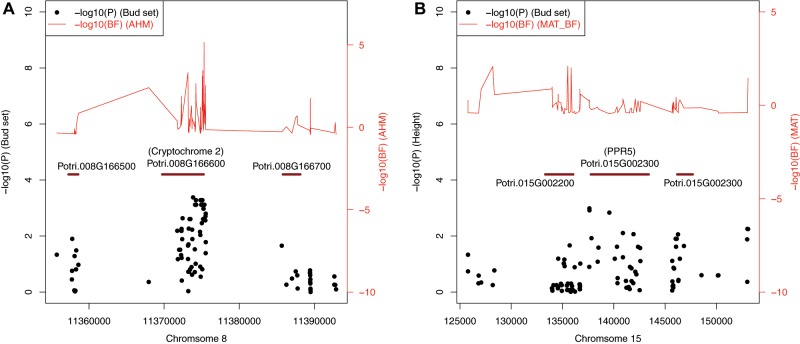
—Scatterplots of phenotypic associations and climate selection signals around candidate genes Potri.008G166600 (*A*) and Potri.015G002300 (*B*).

The gene-based analysis of structured GWAS and Bayenv2 results identified genes underlying phenotypic adaptation and climatic selection, respectively. Many climate selection signals overlapped with genotype–phenotype associations, which suggests that phenotypic adaptation during colonization of the two transects was driven by selection agents captured by climate variables. For example, Potri.008G166600 (CRY2; Cryptochrome 2) was associated with timing of bud set and annual heat-moisture (AHM) in the latitude transect (fig. 9*A*), and a height-associated gene (Potri.015G002300; PSEUDO RESPONSE REGULATOR 5; PPR5) also contained SNPs strongly associated with MAT and TD (fig. 9*B*). This latter gene was also associated with plant height in an independent GWAS study (McKown, Guy, et al. 2014). Although we identified many cases of convergence between GWAS and climate outliers, there was also extensive discordance between these two methods, which may be due to the two methods scanning the genome for different type of association signatures. That is, the traits we measured for GWAS likely did not capture the full breadth of phenotypes responsive to climatic variation across our sampling sites.

Although there was substantial concordance, many outliers were unique to Bayenv2 or RDA, which may reflect the different logic behind the two methods. Bayenv looks for single SNP allele frequency shift across spatial groups with sample size difference and population structure accounted (Gunther and Coop 2013), while RDA considers multilocus genetic variation across multivariate environment (Forester et al. 2016). However, the common selection outliers detected with different scanning methods provide reliable candidate targets for climate-related selection.

### Comparison to Previous Local Adaptation Studies in Poplar and Other Tree Species

**Fig. 10.**
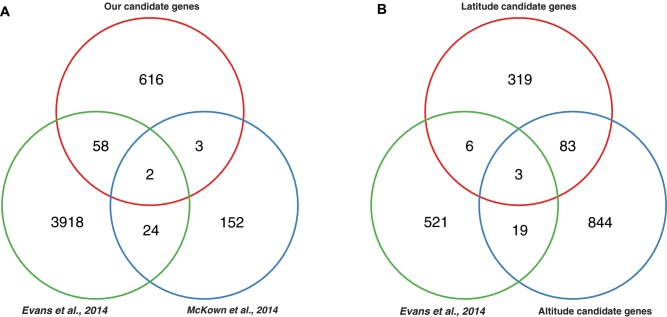
—Venn diagram showing overlap between our bud set candidate genes and those identified in two other studies (Evans et al. 2014; McKown, Guy, et al. 2014; McKown, Klápště, et al. 2014) (*A*). Candidate genes detected with gene-based Bayenv analysis across latitude and altitude transect were compared with Bayenv outliers from Evans et al. (2014) (*B*).

A moderate proportion of our phenotype- and climate-related genes were concordant with findings in two recent studies in *P. trichocarapa* (fig. 10 and supplementary fig. S14, Supplementary Material online), although we did not find a significant enrichment based on a hypergeometric test. Five bud set associated genes overlapping with McKown, Guy, et al. (2014) and McKown, Klápště, et al. (2014), and 60 overlapped with Evans et al. (2014) (fig. 10*A*). The two genes shared by all three studies were Potri.002G165900, an ABA-insensitive like protein that suggests a response to ABA during fall bud set, and Potri.003G214200, a glucan synthase-like protein that may be essential for 1,3-beta glucan synthesis and formation of callose deposition. The latter is particularly interesting as experimental evidence suggests that short-day induced callose deposition seals cell wall plasmodesmata in the shoot apical meristem and disconnects growth regulator transport to the dormant bud, thus maintaining dormancy (van der Schoot and Rinne 2011; Paul et al. 2014). We also found overlap between our Bayenv2 outliers and those identified with Bayenv in Evans et al. (2014) (fig. 10*B*). The conserved environment-associated genes between these studies include Potri.009G058900 (similar to DNA topoisomerase), Potri.012G003300, and Potri.001G359100 (Glycogen branching enzyme). The samples studied in McKown, Guy, et al. (2014) and McKown, Klápště, et al. (2014) mainly covered British Columbia and northern Oregon, whereas those from Evans et al. (2014) span California, Oregon, Washington, and British Columbia. Each of these partially overlapped with our sampling locations. However, neither McKown nor Evans included samples along altitudinal transects. Thus, each of these analyses addressed a different portion of the climate space occupied by *P. trichocarpa*, which may partly explain the outliers that were unique to each study.

Finally, we compared our results with those of three additional temperate/boreal tree species that exhibit similar patterns of local adaptation to climate. Among candidate genes identified in European aspen (*Populus tremula*) by Wang et al. (2018), 19 out of 91 overlapped with candidates in our RDA in both altitude and latitude transects. Among these overlapping genes was Potri.010G179700.1, which encodes FT2, a PEBP family protein that was previously found regulate growth cessation in poplar (Hsu et al. 2011). The FT2 ortholog Potra001246g10694 in *Populustremula* governs clinal variation in bud set timing through variation in its transcript abundance and timing of expression (Wang et al. 2018). In addition, we found six overlapping genes between the local adaptation candidates identified by Wang et al. and our GWAS results, but no overlap with our Bayenv2 outliers. We further evaluated level of overlap between our candidate genes and adaptive loci identified in the much more distantly related interior spruce (*Picea glauca* × *P. engelmanii*) and lodgepole pine (*Pinus contorta*) (Yeaman et al. 2016) and found a relatively large number of overlapping candidates (supplementary table S12, Supplementary Material online). There was significant overlap between both pine and spruce gene outliers and our Bayenv2 candidate genes across altitude (*P* < 0.01 in both hypergeometric tests), and our RDA candidate genes also showed significant overlap across both transects with convergent outliers in both pine and spruce (*P* < 0.001 in hypergeometric tests). The common outliers included several genes with roles in response to abiotic stress or seasonal light signaling: Potri.003G044200 and Potri.006G224600, which are homologs of HAB1 (HYPERSENSITIVE TO ABA1); Potri.004G102100 and Potri.017G112700, which are homologs of the chloroplast protein APE1 (ACCLIMATION OF PHOTOSYNTHESIS TO ENVIRONMENT); Potri.002G089000, which encodes PHYA (PHYTOCHROME A); and Potri.011G119500, which encodes SPA-related 3 (SUPPRESSOR OF PHYA1) (supplementary table S12, Supplementary Material online). The large number of common genes between these studies may reflect that each used the same gene-based strategy to discern candidate genes or contigs, which emphasizes the power of this approach to define constraints in the genotype–phenotype–environment map, even over deep evolutionary time.

## Conclusions

Climate is a primary abiotic constraint directing adaptive change in morphology, phenology, and physiology of plants. We demonstrated that adaptive traits among *P. trichocarpa* populations vary as a function of the local climate at their geographic origin. By employing a gene-based analysis approach to GWAS, we discovered a number of candidate loci underlying timing of bud phenology, biomass, and regeneration ability and found significant genetic parallelism underlying phenotypic adaptation between latitudinal and altitudinal transects. We also used two complementary genotype–environment scans and detected regions of the genome that have been targeted by climate-related selection, particularly temperature and its interaction with heat, and some of these regions overlapped between our altitudinal and latitudinal transects. Taken together, these results suggest some constraint in the genetics of climatic adaptation across latitude and altitude in *P. trichocparpa*. At the same time, adaptation along these two clines involve a large number of unique loci, which may reflect differences in their respective environmental constraints, and possibly also the demographic details of postglacial recolonization.

## Supplementary Material

Supplementary data are available at *Genome Biology and Evolution* online.

## Supplementary Material

evz151_Supplementary_Data

## Notes

**Data deposition:** Sequence data discussed in this study have been deposited at NCBI’s Sequence Read Archive (SRA) under the accession SRP055524.
