# Supplementary material for: Phenotypic and Genomic Local Adaptation across Latitude and Altitude in Populus trichocarpa
Source: Genome Biol Evol. 2019 Jul 22;11(8):2256–72. doi: 10.1093/gbe/evz151 (PMC6735766; doi:10.1093/gbe/evz151)
Supplement: evz151_Supplementary_Data [file evz151_supplementary_data.zip › supplemental_tables_R1.docx]

Table S1. Structured GWAS models selected for phenotypic traits across two altitude and latitude transects.

| Transect | Common garden | Traits | Simple model | 2PC | 5PC | 10PC | Kinship | 2PC+  Kinship |
| --- | --- | --- | --- | --- | --- | --- | --- | --- |
| Altitude | VA | Height |  |  | √ |  |  |  |
|  |  | Bud set |  | √ |  |  |  |  |
|  |  | Bud flush |  | √ |  |  |  |  |
|  |  | Diameter |  | √ |  |  |  |  |
|  |  | Regeneration height |  |  | √ |  |  |  |
|  |  | Regeneration branch number |  | √ |  |  |  |  |
|  |  | Cold |  | √ |  |  |  |  |
|  | BC | Height |  | √ |  |  |  |  |
|  |  | Bud set |  | √ |  |  |  |  |
|  |  | Bud flush | √ |  |  |  |  |  |
| Latitude | VA | Height |  |  | √ |  |  |  |
|  |  | Bud set |  |  | √ |  |  |  |
|  |  | Bud flush |  |  | √ |  |  |  |
|  |  | Diameter |  |  | √ |  |  |  |
|  |  | Regeneration height |  |  | √ |  |  |  |
|  |  | Regeneration branch number |  | √ |  |  |  |  |
|  |  | Cold |  | √ |  |  |  |  |
|  | BC | Height |  |  | √ |  |  |  |
|  |  | Bud set |  | √ |  |  |  |  |
|  |  | Bud flush |  | √ |  |  |  |  |

Table S2. The explanation for climate variable abbreviations.

|  | Abbreviation | Explanation |
| --- | --- | --- |
| Annual climate variable | MAT | Mean annual temperature (°C) |
|  | MWMT | Mean warmest monthly temperature (°C) |
|  | MCMT | Mean coldest monthly temperature ( °C) |
|  | TD | Temperature difference between MWMT and MCMT |
|  | MAP | Mean annual precipitation (mm) |
|  | MSP | Mean annual summer precipitation (mm) |
|  | AHM | Annual heat moisture index |
|  | SHM | Summer heat moisture index |
| Derived climate variable | DD_0 | Chilling days below 0 °C |
|  | DD5 | Growing degree days above 5°C |
|  | DD_18 | Heating degree-days |
|  | DD18 | Cooling degree-days |
|  | NFFD | Number of frost-free days |
|  | FFP | Frost-free period |
|  | bFFP | The day when FFP begins |
|  | eFFP | The day when FFP ends |
|  | PAS | Snow precipitation (mm) |
|  | EMT | Extreme minimum temperature over 30 years |
|  | EXT | Extreme maximum temperature over 30 years |
|  | Eref | Hargreaves reference evaporation |
|  | CMD | Hargreaves climatic moisture deficit (mm) |

Table S3. Correlations among phenotypic traits, geographic variables, and climate variables across altitudinal and latitudinal samples (The red colored cells indicate significant association between variable 1 and variable 2; blue colored cells indicate non-significant association between two variables).

| Var1 | Var2 | Altitude transect | | | Latitude transect | | |
| --- | --- | --- | --- | --- | --- | --- | --- |
|  |  | Pearson Correlation | P-value | Adjusted p-value | Pearson Correlation | P-value | Adjusted p-value |
| AHM | DD_0 | 0.15 | 3.51E-02 | 1.00E+00 | -0.11 | 1.66E-01 | 1.00E+00 |
| AHM | DD5 | 0.26 | 1.67E-04 | 3.87E-02 | 0.13 | 9.93E-02 | 1.00E+00 |
| AHM | Eref | 0.41 | 1.09E-09 | 2.51E-07 | 0.13 | 1.07E-01 | 1.00E+00 |
| AHM | FFP | -0.01 | 9.20E-01 | 1.00E+00 | 0.03 | 7.15E-01 | 1.00E+00 |
| AHM | SHM | 0.74 | 0.00E+00 | 0.00E+00 | 0.93 | 0.00E+00 | 0.00E+00 |
| Bud flush | AHM | -0.24 | 3.91E-04 | 9.02E-02 | 0.17 | 3.08E-02 | 1.00E+00 |
| Bud flush | Cold injury | -0.44 | 6.00E-11 | 1.38E-08 | -0.24 | 2.83E-03 | 6.54E-01 |
| Bud flush | DD_0 | 0.45 | 1.47E-11 | 3.39E-09 | 0.39 | 4.44E-07 | 1.03E-04 |
| Bud flush | DD5 | -0.57 | 0.00E+00 | 0.00E+00 | -0.39 | 2.99E-07 | 6.90E-05 |
| Bud flush | Diameter | -0.46 | 5.23E-12 | 1.21E-09 | -0.37 | 2.32E-06 | 5.36E-04 |
| Bud flush | Elevation | 0.05 | 4.92E-01 | 1.00E+00 | 0.38 | 8.23E-07 | 1.90E-04 |
| Bud flush | Eref | -0.53 | 4.44E-16 | 1.03E-13 | -0.22 | 4.85E-03 | 1.00E+00 |
| Bud flush | FFP | -0.47 | 6.60E-13 | 1.52E-10 | -0.42 | 4.06E-08 | 9.37E-06 |
| Bud flush | Latitude | 0.56 | 0.00E+00 | 0.00E+00 | 0.43 | 2.15E-08 | 4.96E-06 |
| Bud flush | Longitude | -0.34 | 4.68E-07 | 1.08E-04 | 0.17 | 3.53E-02 | 1.00E+00 |
| Bud flush | MAP | 0.08 | 2.67E-01 | 1.00E+00 | -0.31 | 8.51E-05 | 1.97E-02 |
| Bud flush | MAT | -0.56 | 0.00E+00 | 0.00E+00 | -0.41 | 8.58E-08 | 1.98E-05 |
| Bud flush | MCMT | -0.53 | 2.22E-16 | 5.13E-14 | -0.41 | 9.11E-08 | 2.11E-05 |
| Bud flush | MSP | 0.31 | 4.94E-06 | 1.14E-03 | -0.23 | 3.14E-03 | 7.25E-01 |
| Bud flush | MWMT | -0.47 | 1.67E-12 | 3.86E-10 | -0.33 | 2.91E-05 | 6.73E-03 |
| Bud flush | Regenerated branch number | -0.23 | 1.14E-03 | 2.63E-01 | -0.26 | 8.35E-04 | 1.93E-01 |
| Bud flush | Regeneration height | -0.57 | 0.00E+00 | 0.00E+00 | -0.49 | 9.41E-11 | 2.17E-08 |
| Bud flush | SHM | -0.41 | 1.55E-09 | 3.57E-07 | 0.18 | 2.63E-02 | 1.00E+00 |
| Bud flush | TD | 0.40 | 2.32E-09 | 5.36E-07 | 0.30 | 1.38E-04 | 3.19E-02 |
| Bud set | AHM | 0.20 | 3.20E-03 | 7.39E-01 | 0.07 | 3.55E-01 | 1.00E+00 |
| Bud set | Bud flush | -0.66 | 0.00E+00 | 0.00E+00 | -0.50 | 3.00E-11 | 6.92E-09 |
| Bud set | Cold injury | 0.40 | 2.33E-09 | 5.39E-07 | 0.21 | 8.07E-03 | 1.00E+00 |
| Bud set | DD_0 | -0.38 | 2.33E-08 | 5.39E-06 | -0.41 | 8.69E-08 | 2.01E-05 |
| Bud set | DD5 | 0.50 | 1.78E-14 | 4.10E-12 | 0.43 | 1.42E-08 | 3.27E-06 |
| Bud set | Diameter | 0.53 | 2.22E-16 | 5.13E-14 | 0.63 | 0.00E+00 | 0.00E+00 |
| Bud set | Elevation | -0.10 | 1.74E-01 | 1.00E+00 | -0.42 | 5.07E-08 | 1.17E-05 |
| Bud set | Eref | 0.46 | 4.51E-12 | 1.04E-09 | 0.27 | 5.92E-04 | 1.37E-01 |
| Bud set | FFP | 0.41 | 1.56E-09 | 3.60E-07 | 0.43 | 2.39E-08 | 5.52E-06 |
| Bud set | Latitude | -0.46 | 2.65E-12 | 6.12E-10 | -0.27 | 4.75E-04 | 1.10E-01 |
| Bud set | Longitude | 0.33 | 1.20E-06 | 2.77E-04 | -0.36 | 4.14E-06 | 9.57E-04 |
| Bud set | MAP | -0.05 | 4.59E-01 | 1.00E+00 | 0.06 | 4.52E-01 | 1.00E+00 |
| Bud set | MAT | 0.49 | 5.88E-14 | 1.36E-11 | 0.44 | 1.02E-08 | 2.36E-06 |
| Bud set | MCMT | 0.45 | 1.97E-11 | 4.54E-09 | 0.41 | 7.62E-08 | 1.76E-05 |
| Bud set | MSP | -0.24 | 6.12E-04 | 1.41E-01 | -0.01 | 9.13E-01 | 1.00E+00 |
| Bud set | MWMT | 0.42 | 2.11E-10 | 4.87E-08 | 0.39 | 5.43E-07 | 1.26E-04 |
| Bud set | Regenerated branch number | 0.28 | 3.57E-05 | 8.26E-03 | 0.44 | 9.69E-09 | 2.24E-06 |
| Bud set | Regeneration height | 0.62 | 0.00E+00 | 0.00E+00 | 0.63 | 0.00E+00 | 0.00E+00 |
| Bud set | SHM | 0.32 | 2.94E-06 | 6.79E-04 | 0.04 | 5.87E-01 | 1.00E+00 |
| Bud set | TD | -0.32 | 3.09E-06 | 7.13E-04 | -0.25 | 1.62E-03 | 3.74E-01 |
| Cold injury | AHM | 0.07 | 3.43E-01 | 1.00E+00 | -0.08 | 2.90E-01 | 1.00E+00 |
| Cold injury | DD_0 | -0.34 | 6.12E-07 | 1.41E-04 | -0.22 | 4.60E-03 | 1.00E+00 |
| Cold injury | DD5 | 0.36 | 1.51E-07 | 3.49E-05 | 0.20 | 1.29E-02 | 1.00E+00 |
| Cold injury | Elevation | -0.08 | 2.26E-01 | 1.00E+00 | -0.20 | 1.06E-02 | 1.00E+00 |
| Cold injury | Eref | 0.28 | 5.51E-05 | 1.27E-02 | 0.16 | 4.06E-02 | 1.00E+00 |
| Cold injury | FFP | 0.43 | 1.46E-10 | 3.37E-08 | 0.23 | 3.83E-03 | 8.84E-01 |
| Cold injury | Latitude | -0.40 | 4.14E-09 | 9.57E-07 | -0.23 | 2.98E-03 | 6.89E-01 |
| Cold injury | Longitude | 0.16 | 1.94E-02 | 1.00E+00 | -0.08 | 3.47E-01 | 1.00E+00 |
| Cold injury | MAP | 0.05 | 5.01E-01 | 1.00E+00 | 0.15 | 6.01E-02 | 1.00E+00 |
| Cold injury | MAT | 0.38 | 1.34E-08 | 3.10E-06 | 0.22 | 6.27E-03 | 1.00E+00 |
| Cold injury | MCMT | 0.42 | 5.20E-10 | 1.20E-07 | 0.21 | 7.33E-03 | 1.00E+00 |
| Cold injury | MSP | -0.13 | 6.50E-02 | 1.00E+00 | 0.11 | 1.74E-01 | 1.00E+00 |
| Cold injury | MWMT | 0.20 | 3.93E-03 | 9.07E-01 | 0.16 | 4.06E-02 | 1.00E+00 |
| Cold injury | Regenerated branch number | 0.09 | 2.03E-01 | 1.00E+00 | 0.19 | 1.71E-02 | 1.00E+00 |
| Cold injury | Regeneration height | 0.26 | 1.52E-04 | 3.51E-02 | 0.21 | 7.37E-03 | 1.00E+00 |
| Cold injury | SHM | 0.27 | 1.18E-04 | 2.73E-02 | -0.07 | 3.74E-01 | 1.00E+00 |
| Cold injury | TD | -0.41 | 9.99E-10 | 2.31E-07 | -0.16 | 4.59E-02 | 1.00E+00 |
| DD_0 | DD5 | -0.77 | 0.00E+00 | 0.00E+00 | -0.92 | 0.00E+00 | 0.00E+00 |
| DD_0 | Eref | -0.64 | 0.00E+00 | 0.00E+00 | -0.74 | 0.00E+00 | 0.00E+00 |
| DD_0 | FFP | -0.77 | 0.00E+00 | 0.00E+00 | -0.98 | 0.00E+00 | 0.00E+00 |
| DD5 | Eref | 0.88 | 0.00E+00 | 0.00E+00 | 0.88 | 0.00E+00 | 0.00E+00 |
| DD5 | FFP | 0.81 | 0.00E+00 | 0.00E+00 | 0.94 | 0.00E+00 | 0.00E+00 |
| Diameter | AHM | 0.05 | 5.14E-01 | 1.00E+00 | 0.06 | 4.92E-01 | 1.00E+00 |
| Diameter | Cold injury | 0.19 | 6.89E-03 | 1.00E+00 | 0.25 | 1.68E-03 | 3.88E-01 |
| Diameter | DD_0 | -0.20 | 4.32E-03 | 9.98E-01 | -0.37 | 2.37E-06 | 5.48E-04 |
| Diameter | DD5 | 0.27 | 1.12E-04 | 2.59E-02 | 0.38 | 9.44E-07 | 2.18E-04 |
| Diameter | Elevation | -0.19 | 7.45E-03 | 1.00E+00 | -0.37 | 1.79E-06 | 4.13E-04 |
| Diameter | Eref | 0.19 | 6.72E-03 | 1.00E+00 | 0.26 | 1.05E-03 | 2.42E-01 |
| Diameter | FFP | 0.25 | 3.26E-04 | 7.53E-02 | 0.37 | 2.02E-06 | 4.66E-04 |
| Diameter | Latitude | -0.17 | 1.25E-02 | 1.00E+00 | -0.16 | 5.08E-02 | 1.00E+00 |
| Diameter | Longitude | 0.12 | 7.60E-02 | 1.00E+00 | -0.27 | 5.39E-04 | 1.25E-01 |
| Diameter | MAP | 0.03 | 7.16E-01 | 1.00E+00 | 0.08 | 3.14E-01 | 1.00E+00 |
| Diameter | MAT | 0.27 | 1.17E-04 | 2.70E-02 | 0.38 | 6.78E-07 | 1.57E-04 |
| Diameter | MCMT | 0.24 | 4.20E-04 | 9.71E-02 | 0.37 | 2.26E-06 | 5.22E-04 |
| Diameter | MSP | -0.08 | 2.81E-01 | 1.00E+00 | -0.01 | 8.97E-01 | 1.00E+00 |
| Diameter | MWMT | 0.21 | 2.04E-03 | 4.71E-01 | 0.35 | 6.22E-06 | 1.44E-03 |
| Diameter | Regenerated branch number | 0.58 | 0.00E+00 | 0.00E+00 | 0.65 | 0.00E+00 | 0.00E+00 |
| Diameter | Regeneration height | 0.77 | 0.00E+00 | 0.00E+00 | 0.75 | 0.00E+00 | 0.00E+00 |
| Diameter | SHM | 0.04 | 5.82E-01 | 1.00E+00 | 0.08 | 3.12E-01 | 1.00E+00 |
| Diameter | TD | -0.18 | 7.77E-03 | 1.00E+00 | -0.22 | 6.47E-03 | 1.00E+00 |
| Elevation | AHM | 0.21 | 2.78E-03 | 6.42E-01 | -0.19 | 1.98E-02 | 1.00E+00 |
| Elevation | DD_0 | 0.34 | 5.94E-07 | 1.37E-04 | 0.96 | 0.00E+00 | 0.00E+00 |
| Elevation | DD5 | -0.13 | 6.06E-02 | 1.00E+00 | -0.97 | 0.00E+00 | 0.00E+00 |
| Elevation | Eref | 0.20 | 4.05E-03 | 9.36E-01 | -0.84 | 0.00E+00 | 0.00E+00 |
| Elevation | FFP | -0.43 | 1.00E-10 | 2.32E-08 | -0.95 | 0.00E+00 | 0.00E+00 |
| Elevation | MAP | -0.23 | 9.48E-04 | 2.19E-01 | -0.18 | 2.14E-02 | 1.00E+00 |
| Elevation | MAT | -0.23 | 9.74E-04 | 2.25E-01 | -0.98 | 0.00E+00 | 0.00E+00 |
| Elevation | MCMT | -0.28 | 3.54E-05 | 8.18E-03 | -0.90 | 0.00E+00 | 0.00E+00 |
| Elevation | MSP | -0.23 | 6.92E-04 | 1.60E-01 | 0.07 | 4.15E-01 | 1.00E+00 |
| Elevation | MWMT | 0.04 | 5.46E-01 | 1.00E+00 | -0.91 | 0.00E+00 | 0.00E+00 |
| Elevation | SHM | 0.26 | 1.76E-04 | 4.07E-02 | -0.31 | 8.61E-05 | 1.99E-02 |
| Elevation | TD | 0.38 | 2.19E-08 | 5.06E-06 | 0.49 | 4.61E-11 | 1.06E-08 |
| FFP | Eref | 0.49 | 1.49E-13 | 3.44E-11 | 0.72 | 0.00E+00 | 0.00E+00 |
| Height | AHM | 0.10 | 1.39E-01 | 1.00E+00 | 0.06 | 4.83E-01 | 1.00E+00 |
| Height | Bud flush | -0.56 | 0.00E+00 | 0.00E+00 | -0.41 | 8.97E-08 | 2.07E-05 |
| Height | Bud set | 0.59 | 0.00E+00 | 0.00E+00 | 0.59 | 2.22E-16 | 5.13E-14 |
| Height | Cold injury | 0.22 | 1.41E-03 | 3.26E-01 | 0.26 | 8.12E-04 | 1.88E-01 |
| Height | DD_0 | -0.34 | 4.44E-07 | 1.03E-04 | -0.36 | 4.34E-06 | 1.00E-03 |
| Height | DD5 | 0.40 | 3.78E-09 | 8.74E-07 | 0.37 | 1.78E-06 | 4.12E-04 |
| Height | Diameter | 0.87 | 0.00E+00 | 0.00E+00 | 0.85 | 0.00E+00 | 0.00E+00 |
| Height | Elevation | -0.23 | 1.06E-03 | 2.45E-01 | -0.36 | 4.07E-06 | 9.41E-04 |
| Height | Eref | 0.31 | 4.99E-06 | 1.15E-03 | 0.25 | 1.52E-03 | 3.51E-01 |
| Height | FFP | 0.37 | 5.89E-08 | 1.36E-05 | 0.36 | 3.58E-06 | 8.27E-04 |
| Height | Latitude | -0.32 | 3.40E-06 | 7.85E-04 | -0.19 | 1.81E-02 | 1.00E+00 |
| Height | Longitude | 0.20 | 4.75E-03 | 1.00E+00 | -0.27 | 7.64E-04 | 1.77E-01 |
| Height | MAP | 0.03 | 6.42E-01 | 1.00E+00 | 0.07 | 3.67E-01 | 1.00E+00 |
| Height | MAT | 0.41 | 1.01E-09 | 2.33E-07 | 0.37 | 1.28E-06 | 2.96E-04 |
| Height | MCMT | 0.39 | 4.66E-09 | 1.08E-06 | 0.36 | 3.80E-06 | 8.77E-04 |
| Height | MSP | -0.12 | 9.07E-02 | 1.00E+00 | 0.00 | 9.56E-01 | 1.00E+00 |
| Height | MWMT | 0.31 | 6.02E-06 | 1.39E-03 | 0.34 | 1.62E-05 | 3.75E-03 |
| Height | Regenerated branch number | 0.56 | 0.00E+00 | 0.00E+00 | 0.62 | 0.00E+00 | 0.00E+00 |
| Height | Regeneration height | 0.81 | 0.00E+00 | 0.00E+00 | 0.79 | 0.00E+00 | 0.00E+00 |
| Height | SHM | 0.15 | 3.62E-02 | 1.00E+00 | 0.06 | 4.43E-01 | 1.00E+00 |
| Height | TD | -0.32 | 2.95E-06 | 6.80E-04 | -0.22 | 6.09E-03 | 1.00E+00 |
| Latitude | AHM | -0.27 | 7.87E-05 | 1.82E-02 | 0.58 | 8.88E-16 | 2.05E-13 |
| Latitude | DD_0 | 0.73 | 0.00E+00 | 0.00E+00 | 0.54 | 2.07E-13 | 4.78E-11 |
| Latitude | DD5 | -0.85 | 0.00E+00 | 0.00E+00 | -0.55 | 7.57E-14 | 1.75E-11 |
| Latitude | Elevation | -0.19 | 5.21E-03 | 1.00E+00 | 0.51 | 1.03E-11 | 2.39E-09 |
| Latitude | Eref | -0.90 | 0.00E+00 | 0.00E+00 | -0.43 | 1.48E-08 | 3.42E-06 |
| Latitude | FFP | -0.65 | 0.00E+00 | 0.00E+00 | -0.61 | 0.00E+00 | 0.00E+00 |
| Latitude | Longitude | -0.63 | 0.00E+00 | 0.00E+00 | -0.10 | 2.03E-01 | 1.00E+00 |
| Latitude | MAP | -0.01 | 8.95E-01 | 1.00E+00 | -0.73 | 0.00E+00 | 0.00E+00 |
| Latitude | MAT | -0.86 | 0.00E+00 | 0.00E+00 | -0.57 | 7.99E-15 | 1.85E-12 |
| Latitude | MCMT | -0.82 | 0.00E+00 | 0.00E+00 | -0.51 | 5.21E-12 | 1.20E-09 |
| Latitude | MSP | 0.46 | 2.35E-12 | 5.44E-10 | -0.68 | 0.00E+00 | 0.00E+00 |
| Latitude | MWMT | -0.74 | 0.00E+00 | 0.00E+00 | -0.43 | 1.72E-08 | 3.98E-06 |
| Latitude | SHM | -0.73 | 0.00E+00 | 0.00E+00 | 0.59 | 2.22E-16 | 5.13E-14 |
| Latitude | TD | 0.61 | 0.00E+00 | 0.00E+00 | 0.36 | 4.26E-06 | 9.85E-04 |
| Longitude | AHM | 0.09 | 2.02E-01 | 1.00E+00 | -0.66 | 0.00E+00 | 0.00E+00 |
| Longitude | DD_0 | -0.33 | 1.53E-06 | 3.53E-04 | 0.58 | 1.78E-15 | 4.10E-13 |
| Longitude | DD5 | 0.52 | 8.88E-16 | 2.05E-13 | -0.46 | 8.69E-10 | 2.01E-07 |
| Longitude | Elevation | 0.24 | 5.78E-04 | 1.34E-01 | 0.52 | 2.85E-12 | 6.59E-10 |
| Longitude | Eref | 0.59 | 0.00E+00 | 0.00E+00 | -0.25 | 1.63E-03 | 3.76E-01 |
| Longitude | FFP | 0.25 | 3.54E-04 | 8.18E-02 | -0.52 | 1.45E-12 | 3.34E-10 |
| Longitude | MAP | 0.06 | 3.93E-01 | 1.00E+00 | 0.50 | 1.66E-11 | 3.84E-09 |
| Longitude | MAT | 0.47 | 8.01E-13 | 1.85E-10 | -0.53 | 1.13E-12 | 2.61E-10 |
| Longitude | MCMT | 0.31 | 4.99E-06 | 1.15E-03 | -0.60 | 0.00E+00 | 0.00E+00 |
| Longitude | MSP | -0.23 | 7.83E-04 | 1.81E-01 | 0.66 | 0.00E+00 | 0.00E+00 |
| Longitude | MWMT | 0.59 | 0.00E+00 | 0.00E+00 | -0.41 | 1.16E-07 | 2.68E-05 |
| Longitude | SHM | 0.28 | 5.65E-05 | 1.31E-02 | -0.61 | 0.00E+00 | 0.00E+00 |
| Longitude | TD | -0.06 | 4.21E-01 | 1.00E+00 | 0.51 | 1.31E-11 | 3.02E-09 |
| MAP | AHM | -0.83 | 0.00E+00 | 0.00E+00 | -0.91 | 0.00E+00 | 0.00E+00 |
| MAP | DD_0 | -0.39 | 5.94E-09 | 1.37E-06 | -0.21 | 7.11E-03 | 1.00E+00 |
| MAP | DD5 | 0.10 | 1.74E-01 | 1.00E+00 | 0.23 | 4.14E-03 | 9.56E-01 |
| MAP | Eref | -0.11 | 1.22E-01 | 1.00E+00 | 0.22 | 6.66E-03 | 1.00E+00 |
| MAP | FFP | 0.30 | 1.30E-05 | 3.00E-03 | 0.28 | 2.86E-04 | 6.60E-02 |
| MAP | MSP | 0.82 | 0.00E+00 | 0.00E+00 | 0.94 | 0.00E+00 | 0.00E+00 |
| MAP | SHM | -0.51 | 4.00E-15 | 9.23E-13 | -0.78 | 0.00E+00 | 0.00E+00 |
| MAT | AHM | 0.12 | 8.43E-02 | 1.00E+00 | 0.11 | 1.63E-01 | 1.00E+00 |
| MAT | DD_0 | -0.91 | 0.00E+00 | 0.00E+00 | -0.97 | 0.00E+00 | 0.00E+00 |
| MAT | DD5 | 0.97 | 0.00E+00 | 0.00E+00 | 0.98 | 0.00E+00 | 0.00E+00 |
| MAT | Eref | 0.83 | 0.00E+00 | 0.00E+00 | 0.81 | 0.00E+00 | 0.00E+00 |
| MAT | FFP | 0.86 | 0.00E+00 | 0.00E+00 | 0.98 | 0.00E+00 | 0.00E+00 |
| MAT | MAP | 0.21 | 2.28E-03 | 5.27E-01 | 0.23 | 2.97E-03 | 6.85E-01 |
| MAT | MCMT | 0.95 | 0.00E+00 | 0.00E+00 | 0.93 | 0.00E+00 | 0.00E+00 |
| MAT | MSP | -0.24 | 4.42E-04 | 1.02E-01 | -0.02 | 8.18E-01 | 1.00E+00 |
| MAT | MWMT | 0.83 | 0.00E+00 | 0.00E+00 | 0.91 | 0.00E+00 | 0.00E+00 |
| MAT | SHM | 0.54 | 0.00E+00 | 0.00E+00 | 0.23 | 4.28E-03 | 9.90E-01 |
| MAT | TD | -0.72 | 0.00E+00 | 0.00E+00 | -0.54 | 2.07E-13 | 4.77E-11 |
| MCMT | AHM | 0.02 | 7.65E-01 | 1.00E+00 | 0.07 | 3.49E-01 | 1.00E+00 |
| MCMT | DD_0 | -0.95 | 0.00E+00 | 0.00E+00 | -0.97 | 0.00E+00 | 0.00E+00 |
| MCMT | DD5 | 0.85 | 0.00E+00 | 0.00E+00 | 0.85 | 0.00E+00 | 0.00E+00 |
| MCMT | Eref | 0.70 | 0.00E+00 | 0.00E+00 | 0.59 | 4.44E-16 | 1.03E-13 |
| MCMT | FFP | 0.88 | 0.00E+00 | 0.00E+00 | 0.97 | 0.00E+00 | 0.00E+00 |
| MCMT | MAP | 0.27 | 9.65E-05 | 2.23E-02 | 0.22 | 6.66E-03 | 1.00E+00 |
| MCMT | MSP | -0.18 | 1.03E-02 | 1.00E+00 | -0.07 | 3.66E-01 | 1.00E+00 |
| MCMT | SHM | 0.47 | 6.96E-13 | 1.61E-10 | 0.17 | 3.63E-02 | 1.00E+00 |
| MCMT | TD | -0.90 | 0.00E+00 | 0.00E+00 | -0.80 | 0.00E+00 | 0.00E+00 |
| MSP | AHM | -0.74 | 0.00E+00 | 0.00E+00 | -0.93 | 0.00E+00 | 0.00E+00 |
| MSP | DD_0 | 0.06 | 3.85E-01 | 1.00E+00 | 0.06 | 4.90E-01 | 1.00E+00 |
| MSP | DD5 | -0.32 | 2.11E-06 | 4.88E-04 | 0.00 | 9.99E-01 | 1.00E+00 |
| MSP | Eref | -0.51 | 4.88E-15 | 1.13E-12 | 0.05 | 5.31E-01 | 1.00E+00 |
| MSP | FFP | -0.06 | 4.00E-01 | 1.00E+00 | 0.02 | 8.24E-01 | 1.00E+00 |
| MSP | SHM | -0.77 | 0.00E+00 | 0.00E+00 | -0.89 | 0.00E+00 | 0.00E+00 |
| MWMT | AHM | 0.26 | 1.65E-04 | 3.82E-02 | 0.21 | 6.79E-03 | 1.00E+00 |
| MWMT | DD_0 | -0.63 | 0.00E+00 | 0.00E+00 | -0.82 | 0.00E+00 | 0.00E+00 |
| MWMT | DD5 | 0.90 | 0.00E+00 | 0.00E+00 | 0.97 | 0.00E+00 | 0.00E+00 |
| MWMT | Eref | 0.90 | 0.00E+00 | 0.00E+00 | 0.92 | 0.00E+00 | 0.00E+00 |
| MWMT | FFP | 0.52 | 1.55E-15 | 3.59E-13 | 0.83 | 0.00E+00 | 0.00E+00 |
| MWMT | MAP | 0.06 | 3.92E-01 | 1.00E+00 | 0.15 | 5.75E-02 | 1.00E+00 |
| MWMT | MCMT | 0.63 | 0.00E+00 | 0.00E+00 | 0.71 | 0.00E+00 | 0.00E+00 |
| MWMT | MSP | -0.32 | 3.48E-06 | 8.03E-04 | -0.04 | 5.80E-01 | 1.00E+00 |
| MWMT | SHM | 0.56 | 0.00E+00 | 0.00E+00 | 0.36 | 3.35E-06 | 7.73E-04 |
| MWMT | TD | -0.22 | 1.21E-03 | 2.80E-01 | -0.15 | 5.43E-02 | 1.00E+00 |
| Regenerated branch number | AHM | -0.04 | 5.42E-01 | 1.00E+00 | 0.20 | 1.17E-02 | 1.00E+00 |
| Regenerated branch number | DD_0 | -0.20 | 3.55E-03 | 8.21E-01 | -0.40 | 2.19E-07 | 5.06E-05 |
| Regenerated branch number | DD5 | 0.19 | 7.13E-03 | 1.00E+00 | 0.40 | 2.15E-07 | 4.96E-05 |
| Regenerated branch number | Elevation | -0.23 | 9.19E-04 | 2.12E-01 | -0.38 | 9.99E-07 | 2.31E-04 |
| Regenerated branch number | Eref | 0.10 | 1.50E-01 | 1.00E+00 | 0.23 | 4.13E-03 | 9.55E-01 |
| Regenerated branch number | FFP | 0.22 | 1.90E-03 | 4.39E-01 | 0.41 | 1.07E-07 | 2.47E-05 |
| Regenerated branch number | Latitude | -0.10 | 1.42E-01 | 1.00E+00 | -0.06 | 4.83E-01 | 1.00E+00 |
| Regenerated branch number | Longitude | 0.13 | 7.13E-02 | 1.00E+00 | -0.43 | 1.90E-08 | 4.40E-06 |
| Regenerated branch number | MAP | 0.05 | 5.07E-01 | 1.00E+00 | -0.06 | 4.42E-01 | 1.00E+00 |
| Regenerated branch number | MAT | 0.21 | 2.52E-03 | 5.81E-01 | 0.41 | 8.13E-08 | 1.88E-05 |
| Regenerated branch number | MCMT | 0.20 | 4.66E-03 | 1.00E+00 | 0.43 | 2.02E-08 | 4.67E-06 |
| Regenerated branch number | MSP | -0.08 | 2.79E-01 | 1.00E+00 | -0.19 | 1.85E-02 | 1.00E+00 |
| Regenerated branch number | MWMT | 0.15 | 3.21E-02 | 1.00E+00 | 0.37 | 1.32E-06 | 3.05E-04 |
| Regenerated branch number | SHM | -0.01 | 9.33E-01 | 1.00E+00 | 0.24 | 2.90E-03 | 6.70E-01 |
| Regenerated branch number | TD | -0.16 | 2.03E-02 | 1.00E+00 | -0.29 | 2.66E-04 | 6.15E-02 |
| Regeneration height | AHM | 0.01 | 8.40E-01 | 1.00E+00 | 0.06 | 4.50E-01 | 1.00E+00 |
| Regeneration height | DD_0 | -0.29 | 1.77E-05 | 4.10E-03 | -0.42 | 2.71E-08 | 6.26E-06 |
| Regeneration height | DD5 | 0.34 | 5.47E-07 | 1.26E-04 | 0.46 | 8.70E-10 | 2.01E-07 |
| Regeneration height | Elevation | -0.18 | 1.04E-02 | 1.00E+00 | -0.43 | 1.41E-08 | 3.25E-06 |
| Regeneration height | Eref | 0.26 | 1.77E-04 | 4.08E-02 | 0.26 | 1.10E-03 | 2.53E-01 |
| Regeneration height | FFP | 0.31 | 7.86E-06 | 1.81E-03 | 0.45 | 2.02E-09 | 4.67E-07 |
| Regeneration height | Latitude | -0.25 | 2.98E-04 | 6.89E-02 | -0.26 | 9.61E-04 | 2.22E-01 |
| Regeneration height | Longitude | 0.23 | 9.67E-04 | 2.23E-01 | -0.40 | 2.73E-07 | 6.31E-05 |
| Regeneration height | MAP | 0.05 | 4.47E-01 | 1.00E+00 | 0.09 | 2.80E-01 | 1.00E+00 |
| Regeneration height | MAT | 0.35 | 2.14E-07 | 4.95E-05 | 0.46 | 8.30E-10 | 1.92E-07 |
| Regeneration height | MCMT | 0.32 | 3.19E-06 | 7.38E-04 | 0.44 | 5.76E-09 | 1.33E-06 |
| Regeneration height | MSP | -0.08 | 2.54E-01 | 1.00E+00 | 0.00 | 9.99E-01 | 1.00E+00 |
| Regeneration height | MWMT | 0.29 | 1.88E-05 | 4.34E-03 | 0.42 | 3.73E-08 | 8.62E-06 |
| Regeneration height | Regenerated branch number | 0.56 | 0.00E+00 | 0.00E+00 | 0.64 | 0.00E+00 | 0.00E+00 |
| Regeneration height | SHM | 0.04 | 5.64E-01 | 1.00E+00 | 0.05 | 5.63E-01 | 1.00E+00 |
| Regeneration height | TD | -0.23 | 7.69E-04 | 1.78E-01 | -0.27 | 7.29E-04 | 1.69E-01 |
| SHM | DD_0 | -0.35 | 3.56E-07 | 8.23E-05 | -0.22 | 6.48E-03 | 1.00E+00 |
| SHM | DD5 | 0.61 | 0.00E+00 | 0.00E+00 | 0.25 | 1.24E-03 | 2.87E-01 |
| SHM | Eref | 0.77 | 0.00E+00 | 0.00E+00 | 0.29 | 2.66E-04 | 6.15E-02 |
| SHM | FFP | 0.32 | 3.85E-06 | 8.88E-04 | 0.14 | 8.57E-02 | 1.00E+00 |
| TD | AHM | 0.12 | 8.32E-02 | 1.00E+00 | 0.08 | 3.39E-01 | 1.00E+00 |
| TD | DD_0 | 0.84 | 0.00E+00 | 0.00E+00 | 0.67 | 0.00E+00 | 0.00E+00 |
| TD | DD5 | -0.55 | 0.00E+00 | 0.00E+00 | -0.38 | 1.09E-06 | 2.52E-04 |
| TD | Eref | -0.36 | 8.76E-08 | 2.02E-05 | -0.04 | 6.40E-01 | 1.00E+00 |
| TD | FFP | -0.81 | 0.00E+00 | 0.00E+00 | -0.66 | 0.00E+00 | 0.00E+00 |
| TD | MAP | -0.30 | 1.01E-05 | 2.34E-03 | -0.17 | 2.96E-02 | 1.00E+00 |
| TD | MSP | 0.05 | 5.13E-01 | 1.00E+00 | 0.06 | 4.18E-01 | 1.00E+00 |
| TD | SHM | -0.28 | 5.47E-05 | 1.26E-02 | 0.07 | 3.68E-01 | 1.00E+00 |

Table S4. Significant GO terms in biological process enriched for phenotype-associated candidate genes across altitude transect.

| GO.ID | Trait | Term | P-value |
| --- | --- | --- | --- |
| GO:0006270 | BCBudset | DNA replication initiation | 0.045 |
| GO:0055085 | BCBudset | transmembrane transport | 0.031 |
| GO:0003006 | BCHeight | developmental process involved in reproduction | 0.04 |
| GO:0007186 | BCHeight | G-protein coupled receptor signaling pathway | 0.045 |
| GO:0016070 | BCHeight | RNA metabolic process | 0.029 |
| GO:0019748 | BCHeight | secondary metabolic process | 0.025 |
| GO:0044702 | BCHeight | single organism reproductive process | 0.021 |
| GO:0051641 | BCHeight | cellular localization | 0.032 |
| GO:0051649 | BCHeight | establishment of localization in cell | 0.026 |
| GO:0090304 | BCHeight | nucleic acid metabolic process | 0.024 |
| GO:1901360 | BCHeight | organic cyclic compound metabolic process | 0.043 |
| GO:1901617 | BCHeight | organic hydroxy compound biosynthetic process | 0.041 |
| GO:1902582 | BCHeight | single-organism intracellular transport | 0.029 |
| GO:0008299 | VABudflush | isoprenoid biosynthetic process | 0.048 |
| GO:0019637 | VABudflush | organophosphate metabolic process | 0.031 |
| GO:0019748 | VABudflush | secondary metabolic process | 0.037 |
| GO:0072521 | VABudflush | purine-containing compound metabolic process | 0.015 |
| GO:1901135 | VABudflush | carbohydrate derivative metabolic process | 0.046 |
| GO:1901657 | VABudflush | glycosyl compound metabolic process | 0.015 |
| GO:0006415 | VABudset | translational termination | 0.033 |
| GO:0033554 | VABudset | cellular response to stress | 0.048 |
| GO:0005975 | VACold | carbohydrate metabolic process | 0.0055 |
| GO:0009163 | VACold | nucleoside biosynthetic process | 0.0475 |
| GO:1901137 | VACold | carbohydrate derivative biosynthetic process | 0.0209 |
| GO:0006520 | VADiameter | cellular amino acid metabolic process | 0.048 |
| GO:0006793 | VADiameter | phosphorus metabolic process | 0.05 |
| GO:0019637 | VADiameter | organophosphate metabolic process | 0.011 |
| GO:0044699 | VADiameter | single-organism process | 0.022 |
| GO:0055085 | VADiameter | transmembrane transport | 0.04 |
| GO:0090407 | VADiameter | organophosphate biosynthetic process | 0.004 |
| GO:1901293 | VADiameter | nucleoside phosphate biosynthetic process | 0.031 |
| GO:0000105 | VAHeight | histidine biosynthetic process | 0.0122 |
| GO:0006022 | VAHeight | aminoglycan metabolic process | 0.0417 |
| GO:0006040 | VAHeight | amino sugar metabolic process | 0.0465 |
| GO:0006081 | VAHeight | cellular aldehyde metabolic process | 0.0324 |
| GO:0006928 | VAHeight | movement of cell or subcellular component process | 0.0267 |
| GO:0009056 | VAHeight | catabolic process | 0.0171 |
| GO:0042180 | VAHeight | cellular ketone metabolic process | 0.0292 |
| GO:0042440 | VAHeight | pigment metabolic process | 0.0497 |
| GO:0044036 | VAHeight | cell wall macromolecule metabolic process | 0.0379 |
| GO:0044248 | VAHeight | cellular catabolic process | 0.0063 |
| GO:0052803 | VAHeight | imidazole-containing compound metabolic process | 0.0197 |
| GO:0071554 | VAHeight | cell wall organization or biogenesis | 0.0314 |
| GO:1901565 | VAHeight | organonitrogen compound catabolic process | 0.0103 |
| GO:1901575 | VAHeight | organic substance catabolic process | 0.0164 |
| GO:0006415 | VAregbranchnum | translational termination | 0.0328 |
| GO:0006520 | VAregbranchnum | cellular amino acid metabolic process | 0.0258 |
| GO:0008152 | VAregbranchnum | metabolic process | 0.0066 |
| GO:0008652 | VAregbranchnum | cellular amino acid biosynthetic process | 0.0489 |
| GO:0044711 | VAregbranchnum | single-organism biosynthetic process | 0.049 |
| GO:0036211 | VAregheight | protein modification process | 0.016 |

Table S5. Significant GO terms in biological process enriched for phenotype-associated candidate genes across latitude transect.

| GO.ID | Trait | Term | P-value |
| --- | --- | --- | --- |
| GO:0006771 | BCBudset | riboflavin metabolic process | 0.013 |
| GO:0009110 | BCBudset | vitamin biosynthetic process | 0.039 |
| GO:0009409 | BCBudset | response to cold | 0.02 |
| GO:0009628 | BCBudset | response to abiotic stimulus | 0.028 |
| GO:0016458 | BCBudset | gene silencing | 0.026 |
| GO:0018130 | BCBudset | heterocycle biosynthetic process | 0.025 |
| GO:0042558 | BCBudset | pteridine-containing compound metabolic process | 0.039 |
| GO:0042726 | BCBudset | flavin-containing compound metabolic process | 0.023 |
| GO:0042727 | BCBudset | flavin-containing compound biosynthetic process | 0.031 |
| GO:1901362 | BCBudset | organic cyclic compound biosynthetic process | 0.029 |
| GO:0044723 | BCHeight | single-organism carbohydrate metabolic process | 0.02 |
| GO:1901617 | BCHeight | organic hydroxy compound biosynthetic process | 0.032 |
| GO:0006081 | VABudflush | cellular aldehyde metabolic process | 0.0356 |
| GO:0006082 | VABudflush | organic acid metabolic process | 0.0038 |
| GO:0006099 | VABudflush | tricarboxylic acid cycle | 0.0261 |
| GO:0006265 | VABudflush | DNA topological change | 0.0473 |
| GO:0015980 | VABudflush | energy derivation by oxidation of organic compounds | 0.0426 |
| GO:0042180 | VABudflush | cellular ketone metabolic process | 0.0324 |
| GO:0044281 | VABudflush | small molecule metabolic process | 0.0048 |
| GO:0044282 | VABudflush | small molecule catabolic process | 0.0332 |
| GO:1901615 | VABudflush | organic hydroxy compound metabolic process | 0.0027 |
| GO:0006520 | VABudset | cellular amino acid metabolic process | 0.04 |
| GO:0006869 | VABudset | lipid transport | 0.038 |
| GO:0015748 | VABudset | organophosphate ester transport | 0.024 |
| GO:0033554 | VABudset | cellular response to stress | 0.048 |
| GO:0006396 | VACold | RNA processing | 0.0087 |
| GO:0016071 | VACold | mRNA metabolic process | 0.0103 |
| GO:0031324 | VACold | negative regulation of cellular metabolic process | 0.0499 |
| GO:0031329 | VACold | regulation of cellular catabolic process | 0.0353 |
| GO:0032269 | VACold | negative regulation of cellular protein process | 0.0257 |
| GO:0051248 | VACold | negative regulation of protein metabolic process | 0.0214 |
| GO:0051302 | VACold | regulation of cell division | 0.0388 |
| GO:0044238 | VADiameter | primary metabolic process | 0.036 |
| GO:0044260 | VADiameter | cellular macromolecule metabolic process | 0.015 |
| GO:0055085 | VADiameter | transmembrane transport | 0.031 |
| GO:0006265 | VAHeight | DNA topological change | 0.047 |
| GO:0006644 | VAHeight | phospholipid metabolic process | 0.011 |
| GO:0008643 | VAHeight | carbohydrate transport | 0.035 |
| GO:0032535 | VAHeight | regulation of cellular component size | 0.042 |
| GO:0032970 | VAHeight | regulation of actin filament-based process | 0.036 |
| GO:0044087 | VAHeight | regulation of cellular component biogenesis | 0.043 |
| GO:0046467 | VAHeight | membrane lipid biosynthetic process | 0.023 |
| GO:0070646 | VAHeight | protein modification by small protein removal | 0.049 |
| GO:0006259 | VAregbranchnum | DNA metabolic process | 0.00036 |
| GO:0006281 | VAregbranchnum | DNA repair | 0.00191 |
| GO:0006302 | VAregbranchnum | double-strand break repair | 0.0285 |
| GO:0016043 | VAregbranchnum | cellular component organization | 0.00397 |
| GO:0017038 | VAregbranchnum | protein import | 0.00845 |
| GO:0033036 | VAregbranchnum | macromolecule localization | 0.00022 |
| GO:0033554 | VAregbranchnum | cellular response to stress | 0.0092 |
| GO:0045184 | VAregbranchnum | establishment of protein localization | 0.000086 |
| GO:0046907 | VAregbranchnum | intracellular transport | 0.00823 |
| GO:0051641 | VAregbranchnum | cellular localization | 0.00596 |
| GO:0051649 | VAregbranchnum | establishment of localization in cell | 0.03198 |
| GO:0051716 | VAregbranchnum | cellular response to stimulus | 0.01058 |
| GO:0071702 | VAregbranchnum | organic substance transport | 0.00324 |
| GO:0071840 | VAregbranchnum | cellular component organization or biogenesis | 0.0143 |
| GO:1902580 | VAregbranchnum | single-organism cellular localization | 0.03775 |
| GO:1990542 | VAregbranchnum | mitochondrial transmembrane transport | 0.03814 |
| GO:0006629 | VAregheight | lipid metabolic process | 0.014 |
| GO:0055085 | VAregheight | transmembrane transport | 0.013 |
| GO:0071804 | VAregheight | cellular potassium ion transport | 0.035 |

Table S6. The overlapping phenotype associated candidate genes identified in gene-based analysis across both altitude and latitude transects.

| Gene_ID | Trait | Transcript_ID | Description | Arabidopsis hit |
| --- | --- | --- | --- | --- |
| Potri.012G106600 | BCBudset | Potri.012G106600.1 | magnesium-protoporphyrin O-methyltransferase |  |
| Potri.015G107000 | VABudset | Potri.015G107000.1 | Regulator of chromosome condensation (RCC1) family protein | AT1G27060.1 |
| Potri.018G003100 | VABudset | Potri.018G003100.1 |  |  |
| Potri.018G003300 | VABudset | Potri.018G003300.1 | Tetratricopeptide repeat (TPR)-like superfamily protein | AT1G10330.1 |
| Potri.018G093900 | VABudset | Potri.018G093900.1 | similar to VDAC3.1 |  |
| Potri.006G082000 | VABudflush | Potri.006G082000.1 | Unknown protein |  |
| Potri.016G088300 | VABudflush | Potri.016G088300.1 | No apical meristem protein (NAM) |  |
| Potri.016G088800 | VABudflush | Potri.016G088800.1 | transducin family protein; WD-40 repeat family protein |  |
| Potri.016G088900 | VABudflush | Potri.016G088900.1 | Conserved peptide upstream open reading frame 47 |  |
| Potri.001G464700 | VADiameter | Potri.001G464700.1 | FAD-binding Berberine family protein | AT5G44440.1 |
| Potri.008G176400 | VADiameter | Potri.008G176400.1 | Unknown protein |  |
| Potri.018G011800 | VADiameter | Potri.018G011800.1 | glutamate receptor, ionotropic, plant (GRIP) |  |
| Potri.001G464700 | VAHeight | Potri.001G464700.1 | FAD-binding Berberine family protein | AT5G44440.1 |
| Potri.002G038300 | VAHeight | Potri.002G038300.1 | translocase inner membrane subunit 44-2 | AT2G36070.1 |

Table S7. Significant GO terms in biological process enriched for climate-associated candidate genes along altitude transect.

| GO.ID | ClimateVar | Term | P-value |
| --- | --- | --- | --- |
| GO:0000271 | AHM | polysaccharide biosynthetic process | 0.041 |
| GO:0009605 | AHM | response to external stimulus | 0.035 |
| GO:0009628 | AHM | response to abiotic stimulus | 0.028 |
| GO:0033692 | AHM | cellular polysaccharide biosynthetic process | 0.042 |
| GO:0008283 | CMD | cell proliferation | 0.016 |
| GO:0043412 | CMD | macromolecule modification | 0.036 |
| GO:0005996 | DD18 | monosaccharide metabolic process | 0.035 |
| GO:0006412 | DD18 | translation | 0.05 |
| GO:0033554 | DD18 | cellular response to stress | 0.048 |
| GO:0043604 | DD18 | amide biosynthetic process | 0.05 |
| GO:0044260 | DD18 | cellular macromolecule metabolic process | 0.015 |
| GO:0055085 | DD18 | transmembrane transport | 0.031 |
| GO:0000723 | DD5 | telomere maintenance | 0.046 |
| GO:0006412 | DD5 | translation | 0.017 |
| GO:0033554 | DD5 | cellular response to stress | 0.048 |
| GO:0043604 | DD5 | amide biosynthetic process | 0.05 |
| GO:0033554 | DD_18 | cellular response to stress | 0.048 |
| GO:0006281 | EMT | DNA repair | 0.043 |
| GO:0016043 | EMT | cellular component organization | 0.032 |
| GO:0033554 | EMT | cellular response to stress | 0.048 |
| GO:0046907 | EMT | intracellular transport | 0.027 |
| GO:0051641 | EMT | cellular localization | 0.045 |
| GO:0051649 | EMT | establishment of localization in cell | 0.039 |
| GO:1902582 | EMT | single-organism intracellular transport | 0.011 |
| GO:1902589 | EMT | single-organism organelle organization | 0.032 |
| GO:0044238 | EXT | primary metabolic process | 0.01 |
| GO:0045185 | EXT | maintenance of protein location | 0.046 |
| GO:0051235 | EXT | maintenance of location | 0.047 |
| GO:0051651 | EXT | maintenance of location in cell | 0.027 |
| GO:0071704 | EXT | organic substance metabolic process | 0.027 |
| GO:1901566 | EXT | organonitrogen compound biosynthetic process | 0.049 |
| GO:0000723 | Elevation | telomere maintenance | 0.039 |
| GO:0006281 | Elevation | DNA repair | 0.043 |
| GO:0016226 | Elevation | iron-sulfur cluster assembly | 0.035 |
| GO:0033554 | Elevation | cellular response to stress | 0.048 |
| GO:0044260 | Elevation | cellular macromolecule metabolic process | 0.021 |
| GO:0006818 | Eref | hydrogen transport | 0.0422 |
| GO:0044238 | Eref | primary metabolic process | 0.0195 |
| GO:0045185 | Eref | maintenance of protein location | 0.0458 |
| GO:0051235 | Eref | maintenance of location | 0.0404 |
| GO:0051651 | Eref | maintenance of location in cell | 0.0199 |
| GO:0055085 | Eref | transmembrane transport | 0.0036 |
| GO:0071704 | Eref | organic substance metabolic process | 0.0453 |
| GO:1901566 | Eref | organonitrogen compound biosynthetic process | 0.0298 |
| GO:1902578 | Eref | single-organism localization | 0.0197 |
| GO:0006457 | FFP | protein folding | 0.048 |
| GO:0033554 | FFP | cellular response to stress | 0.048 |
| GO:0046907 | FFP | intracellular transport | 0.027 |
| GO:0051641 | FFP | cellular localization | 0.045 |
| GO:0051649 | FFP | establishment of localization in cell | 0.039 |
| GO:0065008 | FFP | regulation of biological quality | 0.032 |
| GO:1902582 | FFP | single-organism intracellular transport | 0.011 |
| GO:0005996 | Latitude | monosaccharide metabolic process | 0.0035 |
| GO:0006310 | Latitude | DNA recombination | 0.0337 |
| GO:0033554 | Latitude | cellular response to stress | 0.0478 |
| GO:0009451 | MAP | RNA modification | 0.023 |
| GO:0045185 | MAP | maintenance of protein location | 0.046 |
| GO:0051235 | MAP | maintenance of location | 0.027 |
| GO:0051651 | MAP | maintenance of location in cell | 0.02 |
| GO:0071840 | MAP | cellular component organization or biogenesis | 0.038 |
| GO:1902578 | MAP | single-organism localization | 0.048 |
| GO:0005975 | MAT | carbohydrate metabolic process | 0.02 |
| GO:0033554 | MAT | cellular response to stress | 0.048 |
| GO:0044260 | MAT | cellular macromolecule metabolic process | 0.037 |
| GO:1902589 | MAT | single-organism organelle organization | 0.046 |
| GO:0006412 | MCMT | translation | 0.044 |
| GO:0033554 | MCMT | cellular response to stress | 0.048 |
| GO:1902582 | MCMT | single-organism intracellular transport | 0.029 |
| GO:1902589 | MCMT | single-organism organelle organization | 0.046 |
| GO:0008283 | MSP | cell proliferation | 0.0024 |
| GO:0000723 | MWMT | telomere maintenance | 0.046 |
| GO:0005996 | MWMT | monosaccharide metabolic process | 0.035 |
| GO:0006412 | MWMT | translation | 0.05 |
| GO:0033554 | MWMT | cellular response to stress | 0.048 |
| GO:0043604 | MWMT | amide biosynthetic process | 0.05 |
| GO:0044260 | MWMT | cellular macromolecule metabolic process | 0.015 |
| GO:0055085 | MWMT | transmembrane transport | 0.031 |
| GO:0006412 | NFFD | translation | 0.044 |
| GO:0033554 | NFFD | cellular response to stress | 0.048 |
| GO:0000723 | PAS | telomere maintenance | 0.0388 |
| GO:0006139 | PAS | nucleobase-containing compound metabolic process | 0.0039 |
| GO:0006259 | PAS | DNA metabolic process | 0.0302 |
| GO:0006281 | PAS | DNA repair | 0.0342 |
| GO:0006725 | PAS | cellular aromatic compound metabolic process | 0.0114 |
| GO:0034641 | PAS | cellular nitrogen compound metabolic process | 0.0302 |
| GO:0046483 | PAS | heterocycle metabolic process | 0.0114 |
| GO:0051716 | PAS | cellular response to stimulus | 0.02 |
| GO:0090304 | PAS | nucleic acid metabolic process | 0.0051 |
| GO:1901360 | PAS | organic cyclic compound metabolic process | 0.0057 |
| GO:0008283 | SHM | cell proliferation | 0.0094 |
| GO:0044260 | TD | cellular macromolecule metabolic process | 0.028 |
| GO:1902582 | TD | single-organism intracellular transport | 0.029 |
| GO:0033554 | bFFP | cellular response to stress | 0.048 |
| GO:0065008 | bFFP | regulation of biological quality | 0.032 |
| GO:0033554 | eFFP | cellular response to stress | 0.048 |
| GO:0065008 | eFFP | regulation of biological quality | 0.032 |
| GO:1902582 | eFFP | single-organism intracellular transport | 0.029 |

Table S8. Significant GO terms in biological process enriched for climate-associated candidate genes along latitude transect.

| GO.ID | ClimateVar | Term | P-value |
| --- | --- | --- | --- |
| GO:0006081 | AHM | cellular aldehyde metabolic process | 0.016 |
| GO:0042180 | AHM | cellular ketone metabolic process | 0.015 |
| GO:0044712 | AHM | single-organism catabolic process | 0.011 |
| GO:1901575 | AHM | organic substance catabolic process | 0.034 |
| GO:1901615 | AHM | organic hydroxy compound metabolic process | 0.031 |
| GO:0006694 | bFFP | steroid biosynthetic process | 0.037 |
| GO:0008202 | bFFP | steroid metabolic process | 0.025 |
| GO:0009719 | CMD | response to endogenous stimulus | 0.033 |
| GO:0050794 | CMD | regulation of cellular process | 0.041 |
| GO:0050896 | CMD | response to stimulus | 0.016 |
| GO:0006694 | DD_0 | steroid biosynthetic process | 0.037 |
| GO:0008202 | DD_0 | steroid metabolic process | 0.05 |
| GO:0030163 | DD_0 | protein catabolic process | 0.034 |
| GO:0044257 | DD_0 | cellular protein catabolic process | 0.037 |
| GO:0006694 | DD_18 | steroid biosynthetic process | 0.037 |
| GO:0008202 | DD_18 | steroid metabolic process | 0.05 |
| GO:0009409 | DD_18 | response to cold | 0.02 |
| GO:0030163 | DD_18 | protein catabolic process | 0.034 |
| GO:0044257 | DD_18 | cellular protein catabolic process | 0.037 |
| GO:0009409 | DD5 | response to cold | 0.02 |
| GO:0050896 | DD5 | response to stimulus | 0.028 |
| GO:0006508 | eFFP | proteolysis | 0.0367 |
| GO:0006694 | eFFP | steroid biosynthetic process | 0.0372 |
| GO:0009056 | eFFP | catabolic process | 0.03 |
| GO:0009057 | eFFP | macromolecule catabolic process | 0.003 |
| GO:0030163 | eFFP | protein catabolic process | 0.0011 |
| GO:0044248 | eFFP | cellular catabolic process | 0.0075 |
| GO:0044257 | eFFP | cellular protein catabolic process | 0.0014 |
| GO:0044265 | eFFP | cellular macromolecule catabolic process | 0.002 |
| GO:0051603 | eFFP | proteolysis involved in cellular protein | 0.0268 |
| GO:1901575 | eFFP | organic substance catabolic process | 0.017 |
| GO:0008213 | Elevation | protein alkylation | 0.016 |
| GO:0016570 | Elevation | histone modification | 0.014 |
| GO:0032259 | Elevation | methylation | 0.029 |
| GO:0043414 | Elevation | macromolecule methylation | 0.031 |
| GO:0006694 | EMT | steroid biosynthetic process | 0.0372 |
| GO:0009056 | EMT | catabolic process | 0.0168 |
| GO:0009057 | EMT | macromolecule catabolic process | 0.0058 |
| GO:0030163 | EMT | protein catabolic process | 0.0034 |
| GO:0044248 | EMT | cellular catabolic process | 0.0075 |
| GO:0044257 | EMT | cellular protein catabolic process | 0.004 |
| GO:0044265 | EMT | cellular macromolecule catabolic process | 0.004 |
| GO:0051603 | EMT | proteolysis involved in cellular protein catabolic process | 0.0268 |
| GO:1901575 | EMT | organic substance catabolic process | 0.017 |
| GO:0006694 | FFP | steroid biosynthetic process | 0.037 |
| GO:0008202 | FFP | steroid metabolic process | 0.025 |
| GO:0008213 | MAP | protein alkylation | 0.032 |
| GO:0016570 | MAP | histone modification | 0.028 |
| GO:0032259 | MAP | methylation | 0.046 |
| GO:0033036 | MAP | macromolecule localization | 0.034 |
| GO:0045184 | MAP | establishment of protein localization | 0.025 |
| GO:1902580 | MAP | single-organism cellular localization | 0.046 |
| GO:0006694 | MAT | steroid biosynthetic process | 0.037 |
| GO:0008202 | MAT | steroid metabolic process | 0.05 |
| GO:0009409 | MAT | response to cold | 0.02 |
| GO:0030163 | MAT | protein catabolic process | 0.034 |
| GO:0044257 | MAT | cellular protein catabolic process | 0.037 |
| GO:0006694 | MCMT | steroid biosynthetic process | 0.037 |
| GO:0008202 | MCMT | steroid metabolic process | 0.05 |
| GO:0030163 | MCMT | protein catabolic process | 0.034 |
| GO:0044257 | MCMT | cellular protein catabolic process | 0.037 |
| GO:0006869 | MSP | lipid transport | 0.019 |
| GO:0015748 | MSP | organophosphate ester transport | 0.012 |
| GO:0051179 | MWMT | localization | 0.019 |
| GO:1902578 | MWMT | single-organism localization | 0.05 |
| GO:0006694 | NFFD | steroid biosynthetic process | 0.037 |
| GO:0008202 | NFFD | steroid metabolic process | 0.05 |
| GO:0030163 | NFFD | protein catabolic process | 0.034 |
| GO:0044257 | NFFD | cellular protein catabolic process | 0.037 |
| GO:0009057 | PAS | macromolecule catabolic process | 0.032 |
| GO:0030163 | PAS | protein catabolic process | 0.034 |
| GO:0044248 | PAS | cellular catabolic process | 0.036 |
| GO:0044257 | PAS | cellular protein catabolic process | 0.037 |
| GO:0044265 | PAS | cellular macromolecule catabolic process | 0.027 |
| GO:1901575 | PAS | organic substance catabolic process | 0.043 |
| GO:0009719 | SHM | response to endogenous stimulus | 0.033 |
| GO:0006694 | TD | steroid biosynthetic process | 0.0372 |
| GO:0009056 | TD | catabolic process | 0.023 |
| GO:0009057 | TD | macromolecule catabolic process | 0.0058 |
| GO:0030163 | TD | protein catabolic process | 0.0034 |
| GO:0044248 | TD | cellular catabolic process | 0.0075 |
| GO:0044257 | TD | cellular protein catabolic process | 0.004 |
| GO:0044265 | TD | cellular macromolecule catabolic process | 0.004 |
| GO:0051603 | TD | proteolysis involved in cellular protein catabolic process | 0.0268 |
| GO:1901575 | TD | organic substance catabolic process | 0.017 |

Table S9. Climate associated candidate genes and regions in altitude transect displaying parallel association across latitude transect.

| Gene_ID | Climate  Variable | P-value | Adjustedp-value | Arabidopsis hit | Gene Description |
| --- | --- | --- | --- | --- | --- |
| Potri.011G136100 | AHM | 0.0006 | 0.049 | AT3G20550.1 | similar to DAWDLE |
| Potri.006G275700 | CMD | 0.0018 | 0.042 | AT2G24490.1 | replicon protein A2 |
| Potri.013G098700 | CMD | 0.0007 | 0.042 | AT1G22870.1 | Protein kinase family protein with ARM repeat domain |
| Potri.017G081600 | CMD | 0.0014 | 0.042 | AT5G39865.1 | Glutaredoxin family protein |
| Potri.011G136100 | MSP | 0.0008 | 0.022 | AT3G20550.1 | similar to DAWDLE |
| Potri.001G331200 | PAS | 0.0025 | 0.036 | AT3G27120.1 | P-loop containing nucleoside triphosphate hydrolases superfamily protein |
| Potri.004G019300 | PAS | 0.0038 | 0.036 |  | similar to beta-glucosidase |
| Potri.008G212200 | PAS | 0.0036 | 0.036 |  | similar to putative disease resistance gene analog NBS-LRR |
| Potri.010G133500 | PAS | 0.0025 | 0.036 |  | Unknown |
| Potri.011G136100 | PAS | 0.0004 | 0.024 | AT3G20550.1 | similar to DAWDLE |
| Potri.012G007000 | PAS | 0.0035 | 0.036 |  | Unknown |
| Potri.011G136100 | SHM | 0.0003 | 0.021 | AT3G20550.1 | similar to DAWDLE |

Table S10. Climate associated candidate genes and regions in latitude transect displaying parallel association across altitude transect.

| Gene_ID | Climate  Variable | P-value | Adjusted p-value | Arabidopsis hit | Gene Description |
| --- | --- | --- | --- | --- | --- |
| Potri.001G129600 | AHM | 0.0025 | 0.049 |  | Glycosyl hydrolase family 31 protein |
| Potri.003G205900 | AHM | 0.0021 | 0.049 |  | Unknown function |
| Potri.011G136100 | AHM | 0.0005 | 0.032 | AT3G20550.1 | similar to DAWDLE |
| Potri.006G246100 | DD18 | 0.0030 | 0.044 | AT4G32630.1 | ArfGap/RecO-like zinc finger domain-containing protein |
| Potri.007G002700 | DD_18 | 0.0058 | 0.048 | AT3G13062.2 | Polyketide cyclase/dehydrase and lipid transport superfamily protein |
| Potri.010G133500 | DD_18 | 0.0048 | 0.048 |  | Unknown function |
| Potri.004G100500 | FFP | 0.0259 | 0.050 | AT5G38460.1 | ALG6, ALG8 glycosyltransferase family |
| Potri.006G032900 | FFP | 0.0101 | 0.050 |  | SYNTAXIN-8 |
| Potri.010G154900 | FFP | 0.0256 | 0.050 |  | Unknown function |
| Chr9_624777:626335 | FFP | 0.0222 | 0.050 |  | Genomic Region |
| Potri.001G266400 | Latitude | 0.0048 | 0.013 | AT5G05170.1 | Cellulose synthase family protein |
| Potri.010G133500 | Latitude | 0.0049 | 0.013 |  | Unknown function |
| Potri.002G052500 | SHM | 0.0174 | 0.038 | AT5G26330.1 | Cupredoxin superfamily protein |
| Potri.006G246100 | SHM | 0.0450 | 0.038 | AT4G32630.1 | ArfGap/RecO-like zinc finger domain-containing protein |
| Potri.011G136100 | SHM | 0.0008 | 0.007 | AT3G20550 | similar to DAWDLE |
| Potri.012G127400 | SHM | 0.0237 | 0.038 | AT4G25250.1 | invertase/pectin methylesterase inhibitor family protein |
| Potri.012G127500 | SHM | 0.0384 | 0.038 |  | Unknown function |
| Potri.017G081600 | SHM | 0.0079 | 0.032 | AT5G39865.1 | Glutaredoxin family protein |
| Chr1_34471799:34473062 | SHM | 0.0499 | 0.038 |  | Genomic Region |
| Potri.001G282300 | TD | 0.0130 | 0.013 | AT2G30140.2 | UDP-Glycosyltransferase superfamily protein |
| Potri.003G186500 | TD | 0.0348 | 0.013 | AT1G16180.1 | Serinc-domain containing serine and sphingolipid biosynthesis protein |
| Potri.005G163700 | TD | 0.0257 | 0.013 |  | PHI-1 protein |
| Potri.006G082200 | TD | 0.0033 | 0.011 |  |  |
| Potri.010G133000 | TD | 0.0194 | 0.013 |  | HD domain protein |
| Potri.010G133200 | TD | 0.0211 | 0.013 |  | E3 ubiquitin-protein ligase RHA2 |
| Potri.010G133400 | TD | 0.0275 | 0.013 |  |  |
| Potri.010G133500 | TD | 0.0081 | 0.011 |  | Unknown function |
| Potri.010G154900 | TD | 0.0078 | 0.011 |  | Unknown function |
| Potri.011G129300 | TD | 0.0164 | 0.013 | AT4G27290.1 | S-locus lectin protein kinase family protein |
| Potri.015G019100 | TD | 0.0315 | 0.013 | AT5G53130.1 | cyclic nucleotide gated channel 1 |
| SCAFFOLD_29_29013:29240 | TD | 0.0387 | 0.013 |  | Genomic Region |
| Potri.005G251700 | eFFP | 0.0084 | 0.046 |  | similar to NIN-like protein 1 |
| Potri.010G133500 | eFFP | 0.0043 | 0.046 |  | Unknown function |
| Potri.010G154900 | eFFP | 0.0081 | 0.046 |  | Unknown function |

Table S11. SNP outliers identified with Bayenv2 and RDA analysis and the overlap between them across altitude and latitude transects.

| Transect | Climate predictor | Bayenv2 | RDA | overlap | P-value (Hypergeometric test) |
| --- | --- | --- | --- | --- | --- |
| Altitude | MAT | 18882 | 2367 | 303 | 7.06E-181 |
|  | MWMT | 19078 | 3118 | 178 | 1.16E-51 |
|  | TD | 21373 | 2412 | 246 | 7.44E-113 |
|  | MAP | 7947 | 3157 | 84 | 3.49E-28 |
|  | AHM | 10963 | 778 | 18 | 1.40E-04 |
|  | SHM | 9725 | 1261 | 8 | 7.17E-01 |
|  | FFP | 20308 | 2268 | 242 | 5.32E-120 |
|  | PAS | 10495 | 647 | 60 | 4.25E-43 |
|  | EMT | 18834 | 154 | 9 | 4.25E-04 |
|  | EXT | 28963 | 312 | 59 | 2.18E-36 |
|  | Eref | 25077 | 147 | 7 | 2.36E-02 |
| Latitude | MAT | 5948 | 1566 | 36 | 8.80E-15 |
|  | MWMT | 4120 | 300 | 2 | 2.43E-01 |
|  | TD | 7435 | 410 | 5 | 8.60E-02 |
|  | MAP | 13866 | 917 | 25 | 2.53E-05 |
|  | AHM | 13348 | 994 | 26 | 1.87E-05 |
|  | SHM | 8386 | 9567 | 67 | 2.43E-01 |
|  | FFP | 6304 | 2342 | 19 | 2.14E-02 |
|  | PAS | 3350 | 207 | 1 | 4.11E-01 |
|  | EMT | 7624 | 150 | 1 | 5.83E-01 |
|  | EXT | 4732 | 846 | 3 | 5.89E-01 |
|  | Eref | 4600 | 5394 | 38 | 6.86E-05 |

Table S12. The convergent adaptation outliers identified in Yeaman, *et al.* 2016 found in common with candidate genes in our gene-based GWAS, Bayenv, and RDA analysis.

| Gene_ID | Our analysis | Other species | Arabidopsis hit | Gene description |
| --- | --- | --- | --- | --- |
| Potri.002G027200 | GWAS_altitutde | interior spruce | AT1G19910.1 | ATPase, F0/V0 complex, subunit C protein |
| Potri.002G082700 | GWAS_altitutde | interior spruce | AT1G19910.1 | ATPase, F0/V0 complex, subunit C protein |
| Potri.007G014600 | GWAS_altitutde | interior spruce | AT1G19910.1 | ATPase, F0/V0 complex, subunit C protein |
| Potri.014G132900 | GWAS_altitutde | interior spruce | AT3G62900.1 | CW-type Zinc Finger |
| Potri.001G057200 | GWAS_altitutde | interior spruce | AT5G53150.1 | DNAJ heat shock N-terminal domain-containing protein |
| Potri.003G171200 | GWAS_altitutde | interior spruce | AT5G53150.1 | DNAJ heat shock N-terminal domain-containing protein |
| Potri.015G020200 | GWAS_altitutde | interior spruce | AT5G53150.1 | DNAJ heat shock N-terminal domain-containing protein |
| Potri.015G097500 | GWAS_altitutde | interior spruce | AT5G53150.1 | DNAJ heat shock N-terminal domain-containing protein |
| Potri.003G044200 | GWAS_altitutde | interior spruce | AT1G72770.1 | homology to ABI1 |
| Potri.006G224600 | GWAS_altitutde | interior spruce | AT1G72770.1 | homology to ABI1 |
| Potri.007G024300 | GWAS_altitutde | interior spruce | AT1G75220.1 | Major facilitator superfamily protein |
| Potri.001G366800 | GWAS_altitutde | lodgepole pine | AT5G55630.1 | Outward rectifying potassium channel protein |
| Potri.008G004700 | GWAS_altitutde | lodgepole pine | AT5G55630.1 | Outward rectifying potassium channel protein |
| Potri.008G128100 | GWAS_altitutde | lodgepole pine | AT1G71060.1 | Tetratricopeptide repeat (TPR)-like superfamily protein |
| Potri.007G102500 | GWAS_altitutde | interior spruce | AT1G55860.1 | ubiquitin-protein ligase 1 |
| Potri.011G094100 | GWAS_altitutde | interior spruce | AT1G55860.1 | ubiquitin-protein ligase 1 |
| Potri.015G009500 | GWAS_latitude | lodgepole pine | AT5G24340.1 | 3\-5\ exonuclease domain-containing protein |
| Potri.014G055900 | GWAS_latitude | interior spruce | AT2G44970.1 | alpha/beta-Hydrolases superfamily protein |
| Potri.010G178800 | GWAS_latitude | interior spruce | AT5G36890.1 | beta glucosidase 42 |
| Potri.006G253100 | GWAS_latitude | lodgepole pine | AT5G11170.1 | DEAD/DEAH box RNA helicase family protein |
| Potri.015G053300 | GWAS_latitude | interior spruce | AT1G49050.1 | Eukaryotic aspartyl protease family protein |
| Potri.007G003500 | GWAS_latitude | lodgepole pine | AT1G16720.1 | high chlorophyll fluorescence phenotype 173 |
| Potri.014G000400 | GWAS_latitude | lodgepole pine | AT1G16720.1 | high chlorophyll fluorescence phenotype 173 |
| Potri.010G236300 | GWAS_latitude | interior spruce | AT3G21510.1 | histidine-containing phosphotransmitter 1 |
| Potri.008G161400 | GWAS_latitude | interior spruce | AT5G64560.1 | magnesium transporter 9 |
| Potri.010G207300 | GWAS_latitude | lodgepole pine | AT3G55400.1 | methionyl-tRNA synthetase / methionine--tRNA ligase / MetRS (cpMetRS) |
| Potri.008G144500 | GWAS_latitude | lodgepole pine | AT1G14520.1 | myo-inositol oxygenase 1 |
| Potri.003G087400 | GWAS_latitude | interior spruce | AT4G21700.1 | Protein of unknown function (DUF2921) |
| Potri.011G052500 | GWAS_latitude | interior spruce | AT4G21700.1 | Protein of unknown function (DUF2921) |
| Potri.001G068700 | GWAS_latitude | interior spruce | AT5G15790.1 | RING/U-box superfamily protein |
| Potri.003G161600 | GWAS_latitude | interior spruce | AT5G15790.1 | RING/U-box superfamily protein |
| Potri.004G114800 | GWAS_latitude | interior spruce | AT5G15790.1 | RING/U-box superfamily protein |
| Potri.017G100100 | GWAS_latitude | interior spruce | AT5G15790.1 | RING/U-box superfamily protein |
| Potri.013G099700 | GWAS_latitude | interior spruce | AT1G31480.1 | shoot gravitropism 2 (SGR2) |
| Potri.001G317600 | GWAS_latitude | interior spruce | AT1G04920.1 | sucrose phosphate synthase 3F |
| Potri.017G057800 | GWAS_latitude | interior spruce | AT1G04920.1 | sucrose phosphate synthase 3F |
| Potri.001G203000 | GWAS_latitude | interior spruce | AT5G62650.1 | Tic22-like family protein |
| Potri.007G102500 | GWAS_latitude | interior spruce | AT1G55860.1 | ubiquitin-protein ligase 1 |
| Potri.011G094100 | GWAS_latitude | interior spruce | AT1G55860.1 | ubiquitin-protein ligase 1 |
| Potri.001G346000 | Bayenv_altitutde | interior spruce | AT3G27670.1 | ARM repeat superfamily protein |
| Potri.001G264400 | Bayenv_altitutde | interior spruce | AT3G21640.1 | FKBP-type peptidyl-prolyl cis-trans isomerase family protein |
| Potri.003G077600 | Bayenv_altitutde | lodgepole pine | AT4G17330.1 | G2484-1 protein |
| Potri.004G116400 | Bayenv_altitutde | interior spruce | AT4G26190.1 | Haloacid dehalogenase-like hydrolase (HAD) superfamily protein |
| Potri.010G244200 | Bayenv_altitutde | interior spruce | AT4G26190.1 | Haloacid dehalogenase-like hydrolase (HAD) superfamily protein |
| Potri.015G059000 | Bayenv_altitutde | interior spruce | AT4G26190.1 | Haloacid dehalogenase-like hydrolase (HAD) superfamily protein |
| Potri.007G024300 | Bayenv_altitutde | interior spruce | AT1G75220.1 | Major facilitator superfamily protein |
| Potri.001G007900 | Bayenv_altitutde | interior spruce | AT2G42520.1 | P-loop containing nucleoside triphosphate hydrolases superfamily protein |
| Potri.004G129500 | Bayenv_altitutde | lodgepole pine | AT3G01410.1 | Polynucleotidyl transferase, ribonuclease H-like superfamily protein |
| Potri.008G178200 | Bayenv_altitutde | lodgepole pine | AT1G67580.1 | Protein kinase superfamily protein |
| Potri.015G092100 | Bayenv_altitutde | lodgepole pine | AT1G67580.1 | Protein kinase superfamily protein |
| Potri.007G034900 | Bayenv_altitutde | interior spruce | AT3G50380.1 | Protein of unknown function (DUF1162) |
| Potri.007G035000 | Bayenv_altitutde | interior spruce | AT3G50380.1 | Protein of unknown function (DUF1162) |
| Potri.004G168700 | Bayenv_altitutde | interior spruce | AT4G38760.1 | Protein of unknown function (DUF3414) |
| Potri.011G119500 | Bayenv_altitutde | interior spruce | AT3G15354.1 | SPA1-related 3 |
| Potri.008G147100 | Bayenv_altitutde | lodgepole pine | AT1G60200.1 | splicing factor PWI domain-containing protein |
| Potri.001G203000 | Bayenv_altitutde | interior spruce | AT5G62650.1 | Tic22-like family protein |
| Potri.006G269800 | Bayenv_latitude | lodgepole pine | AT5G51600.1 | Microtubule associated protein (MAP65/ASE1) family protein |
| Potri.014G070100 | Bayenv_latitude | lodgepole pine | AT5G51600.1 | Microtubule associated protein (MAP65/ASE1) family protein |
| Potri.015G131400 | Bayenv_latitude | lodgepole pine | AT5G51600.1 | Microtubule associated protein (MAP65/ASE1) family protein |
| Potri.001G044900 | Bayenv_latitude | interior spruce | AT1G15960.1 | NRAMP metal ion transporter 6 |
| Potri.002G080400 | Bayenv_latitude | interior spruce | AT1G15960.1 | NRAMP metal ion transporter 6 |
| Potri.004G168700 | Bayenv_latitude | interior spruce | AT4G38760.1 | Protein of unknown function (DUF3414) |
| Potri.015G030300 | Bayenv_latitude | lodgepole pine | AT1G53345.1 |  |
| Potri.004G102100 | RDA_altitutde | lodgepole pine | AT5G38660.1 | acclimation of photosynthesis to environment |
| Potri.017G112700 | RDA_altitutde | lodgepole pine | AT5G38660.1 | acclimation of photosynthesis to environment |
| Potri.002G129300 | RDA_altitutde | lodgepole pine | AT4G33300.1 | ADR1-like 1 |
| Potri.015G070800 | RDA_altitutde | lodgepole pine | AT5G13690.1 | alpha-N-acetylglucosaminidase family / NAGLU family |
| Potri.007G023500 | RDA_altitutde | lodgepole pine | AT2G18290.1 | anaphase promoting complex 10 |
| Potri.007G072100 | RDA_altitutde | lodgepole pine | AT5G23190.1 | cytochrome P450, family 86, subfamily B, polypeptide 1 |
| Potri.006G229300 | RDA_altitutde | lodgepole pine | AT4G33010.1 | glycine decarboxylase P-protein 1 |
| Potri.011G126200 | RDA_altitutde | lodgepole pine | AT5G54110.1 | membrane-associated mannitol-induced |
| Potri.014G193400 | RDA_altitutde | lodgepole pine | AT1G04120.1 | multidrug resistance-associated protein 5 |
| Potri.004G161700 | RDA_altitutde | lodgepole pine | AT2G16485.1 | nucleic acid binding;zinc ion binding;DNA binding |
| Potri.007G085500 | RDA_altitutde | lodgepole pine | AT2G22070.1 | pentatricopeptide (PPR) repeat-containing protein |
| Potri.007G057900 | RDA_altitutde | lodgepole pine | AT2G17930.1 | Phosphatidylinositol 3- and 4-kinase family protein with FAT domain |
| Potri.007G058000 | RDA_altitutde | lodgepole pine | AT2G17930.1 | Phosphatidylinositol 3- and 4-kinase family protein with FAT domain |
| Potri.007G057900 | RDA_altitutde | lodgepole pine | AT2G17930.1 | Phosphatidylinositol 3- and 4-kinase family protein with FAT domain |
| Potri.007G058000 | RDA_altitutde | lodgepole pine | AT2G17930.1 | Phosphatidylinositol 3- and 4-kinase family protein with FAT domain |
| Potri.001G046700 | RDA_altitutde | lodgepole pine | AT2G32070.1 | Polynucleotidyl transferase, ribonuclease H-like superfamily protein |
| Potri.006G262500 | RDA_altitutde | lodgepole pine | AT2G32070.1 | Polynucleotidyl transferase, ribonuclease H-like superfamily protein |
| Potri.001G046700 | RDA_altitutde | lodgepole pine | AT2G32070.1 | Polynucleotidyl transferase, ribonuclease H-like superfamily protein |
| Potri.006G262500 | RDA_altitutde | lodgepole pine | AT2G32070.1 | Polynucleotidyl transferase, ribonuclease H-like superfamily protein |
| Potri.011G128000 | RDA_altitutde | lodgepole pine | AT5G54380.1 | protein kinase family protein |
| Potri.014G195100 | RDA_altitutde | lodgepole pine | AT1G35710.1 | Protein kinase family protein with leucine-rich repeat domain |
| Potri.T119600 | RDA_altitutde | lodgepole pine | AT1G35710.1 | Protein kinase family protein with leucine-rich repeat domain |
| Potri.014G195100 | RDA_altitutde | lodgepole pine | AT1G35710.1 | Protein kinase family protein with leucine-rich repeat domain |
| Potri.T119600 | RDA_altitutde | lodgepole pine | AT1G35710.1 | Protein kinase family protein with leucine-rich repeat domain |
| Potri.007G084300 | RDA_altitutde | lodgepole pine | AT4G39620.1 | Tetratricopeptide repeat (TPR)-like superfamily protein |
| Potri.007G084300 | RDA_altitutde | lodgepole pine | AT4G39620.1 | Tetratricopeptide repeat (TPR)-like superfamily protein |
| Potri.002G094100 | RDA_altitutde | lodgepole pine | AT1G78070.1 | Transducin/WD40 repeat-like superfamily protein |
| Potri.006G254000 | RDA_altitutde | lodgepole pine | AT5G25360.1 |  |
| Potri.007G011800 | RDA_altitutde | lodgepole pine | AT5G65540.1 |  |
| Potri.006G254000 | RDA_altitutde | lodgepole pine | AT5G25360.1 |  |
| Potri.007G011800 | RDA_altitutde | lodgepole pine | AT5G65540.1 |  |
| Potri.014G104300 | RDA_latitude | lodgepole pine | AT1G01120.1 | 3-ketoacyl-CoA synthase 1 |
| Potri.004G155600 | RDA_latitude | lodgepole pine | AT1G19440.1 | 3-ketoacyl-CoA synthase 4 |
| Potri.014G196200 | RDA_latitude | lodgepole pine | AT1G19440.1 | 3-ketoacyl-CoA synthase 4 |
| Potri.014G105400 | RDA_latitude | lodgepole pine | AT5G24510.1 | 60S acidic ribosomal protein family |
| Potri.015G004700 | RDA_latitude | lodgepole pine | AT5G24510.1 | 60S acidic ribosomal protein family |
| Potri.015G070800 | RDA_latitude | lodgepole pine | AT5G13690.1 | alpha-N-acetylglucosaminidase family / NAGLU family |
| Potri.010G246100 | RDA_latitude | interior spruce | AT5G04930.1 | aminophospholipid ATPase 1 |
| Potri.010G246200 | RDA_latitude | interior spruce | AT5G04930.1 | aminophospholipid ATPase 1 |
| Potri.015G010400 | RDA_latitude | lodgepole pine | AT5G53480.1 | ARM repeat superfamily protein |
| Potri.002G005000 | RDA_latitude | interior spruce | AT2G33620.1 | AT hook motif DNA-binding family protein |
| Potri.017G039100 | RDA_latitude | interior spruce | AT1G67120.1 | ATPases;nucleotide binding;ATP binding |
| Potri.006G236400 | RDA_latitude | interior spruce | AT2G25740.1 | ATP-dependent protease La (LON) domain protein |
| Potri.001G468700 | RDA_latitude | interior spruce | AT1G55760.1 | BTB/POZ domain-containing protein |
| Potri.010G167000 | RDA_latitude | lodgepole pine | AT1G69510.1 | cAMP-regulated phosphoprotein 19-related protein |
| Potri.010G166900 | RDA_latitude | interior spruce | AT3G53720.1 | cation/H+ exchanger 20 |
| Potri.008G109100 | RDA_latitude | interior spruce | AT1G69060.1 | Chaperone DnaJ-domain superfamily protein |
| Potri.T170800 | RDA_latitude | interior spruce | AT1G69060.1 | Chaperone DnaJ-domain superfamily protein |
| Potri.001G463200 | RDA_latitude | interior spruce | AT3G26710.1 | cofactor assembly of complex C |
| Potri.002G080000 | RDA_latitude | lodgepole pine | AT1G44110.1 | Cyclin A1;1 |
| Potri.006G253100 | RDA_latitude | lodgepole pine | AT5G11170.1 | DEAD/DEAH box RNA helicase family protein |
| Potri.001G061000 | RDA_latitude | lodgepole pine | AT5G43280.1 | delta(3,5),delta(2,4)-dienoyl-CoA isomerase 1 |
| Potri.015G105000 | RDA_latitude | interior spruce | AT5G23720.1 | dual specificity protein phosphatase family protein |
| Potri.008G079300 | RDA_latitude | interior spruce | AT1G65580.1 | Endonuclease/exonuclease/phosphatase family protein |
| Potri.010G177400 | RDA_latitude | interior spruce | AT1G65580.1 | Endonuclease/exonuclease/phosphatase family protein |
| Potri.002G166500 | RDA_latitude | interior spruce | AT3G61590.1 | Galactose oxidase/kelch repeat superfamily protein |
| Potri.014G093200 | RDA_latitude | interior spruce | AT3G61590.1 | Galactose oxidase/kelch repeat superfamily protein |
| Potri.014G128400 | RDA_latitude | interior spruce | AT3G53760.1 | GAMMA-TUBULIN COMPLEX PROTEIN 4 |
| Potri.006G229300 | RDA_latitude | lodgepole pine | AT4G33010.1 | glycine decarboxylase P-protein 1 |
| Potri.006G261800 | RDA_latitude | lodgepole pine | AT4G19810.1 | Glycosyl hydrolase family protein with chitinase insertion domain |
| Potri.007G056400 | RDA_latitude | lodgepole pine | AT2G17820.1 | histidine kinase 1 |
| Potri.002G079100 | RDA_latitude | interior spruce | AT1G77300.1 | histone methyltransferases(H3-K4 specific) |
| Potri.003G044200 | RDA_latitude | interior spruce | AT1G72770.1 | homology to ABI1 |
| Potri.006G224600 | RDA_latitude | interior spruce | AT1G72770.1 | homology to ABI1 |
| Potri.001G317800 | RDA_latitude | interior spruce | AT1G15690.1 | Inorganic H pyrophosphatase family protein |
| Potri.004G205700 | RDA_latitude | interior spruce | AT1G15690.1 | Inorganic H pyrophosphatase family protein |
| Potri.007G073300 | RDA_latitude | interior spruce | AT1G15690.1 | Inorganic H pyrophosphatase family protein |
| Potri.011G079800 | RDA_latitude | interior spruce | AT3G07660.1 | Kinase-related protein of unknown function (DUF1296) |
| Potri.014G169900 | RDA_latitude | interior spruce | AT3G07660.1 | Kinase-related protein of unknown function (DUF1296) |
| Potri.007G082800 | RDA_latitude | interior spruce | AT5G10020.1 | Leucine-rich receptor-like protein kinase family protein |
| Potri.T142300 | RDA_latitude | interior spruce | AT5G10020.1 | Leucine-rich receptor-like protein kinase family protein |
| Potri.007G094500 | RDA_latitude | interior spruce | AT5G10290.1 | leucine-rich repeat transmembrane protein kinase family protein |
| Potri.011G126200 | RDA_latitude | lodgepole pine | AT5G54110.1 | membrane-associated mannitol-induced |
| Potri.004G116500 | RDA_latitude | interior spruce | AT3G29770.1 | methyl esterase 11 |
| Potri.017G095100 | RDA_latitude | interior spruce | AT3G29770.1 | methyl esterase 11 |
| Potri.017G096900 | RDA_latitude | interior spruce | AT3G29770.1 | methyl esterase 11 |
| Potri.014G193400 | RDA_latitude | lodgepole pine | AT1G04120.1 | multidrug resistance-associated protein 5 |
| Potri.001G258700 | RDA_latitude | lodgepole pine | AT5G12870.1 | myb domain protein 46 |
| Potri.015G036300 | RDA_latitude | interior spruce | AT3G17920.1 | Outer arm dynein light chain 1 protein |
| Potri.011G057900 | RDA_latitude | lodgepole pine | AT5G46100.1 | Pentatricopeptide repeat (PPR) superfamily protein |
| Potri.006G223300 | RDA_latitude | lodgepole pine | AT1G05670.1 | Pentatricopeptide repeat (PPR-like) superfamily protein |
| Potri.008G142900 | RDA_latitude | interior spruce | AT5G13770.1 | Pentatricopeptide repeat (PPR-like) superfamily protein |
| Potri.015G076100 | RDA_latitude | lodgepole pine | AT5G62810.1 | peroxin 14 |
| Potri.007G057900 | RDA_latitude | lodgepole pine | AT2G17930.1 | Phosphatidylinositol 3- and 4-kinase family protein with FAT domain |
| Potri.007G058000 | RDA_latitude | lodgepole pine | AT2G17930.1 | Phosphatidylinositol 3- and 4-kinase family protein with FAT domain |
| Potri.002G089000 | RDA_latitude | lodgepole pine | AT1G09570.1 | phytochrome A |
| Potri.001G007900 | RDA_latitude | interior spruce | AT2G42520.1 | P-loop containing nucleoside triphosphate hydrolases superfamily protein |
| Potri.011G128000 | RDA_latitude | lodgepole pine | AT5G54380.1 | protein kinase family protein |
| Potri.008G164700 | RDA_latitude | interior spruce | AT3G23160.1 | Protein of unknown function (DUF668) |
| Potri.001G297200 | RDA_latitude | interior spruce | AT4G33920.1 | Protein phosphatase 2C family protein |
| Potri.007G051900 | RDA_latitude | interior spruce | AT4G33920.1 | Protein phosphatase 2C family protein |
| Potri.001G213900 | RDA_latitude | interior spruce | AT5G22010.1 | replication factor C1 |
| Potri.008G152500 | RDA_latitude | interior spruce | AT5G28060.1 | Ribosomal protein S24e family protein |
| Potri.001G251900 | RDA_latitude | interior spruce | AT5G01450.1 | RING/U-box superfamily protein |
| Potri.014G020900 | RDA_latitude | interior spruce | AT5G01450.1 | RING/U-box superfamily protein |
| Potri.001G319000 | RDA_latitude | interior spruce | AT3G23900.1 | RNA recognition motif (RRM)-containing protein |
| Potri.017G058300 | RDA_latitude | interior spruce | AT3G23900.1 | RNA recognition motif (RRM)-containing protein |
| Potri.017G078800 | RDA_latitude | interior spruce | AT1G13980.1 | sec7 domain-containing protein |
| Potri.011G119500 | RDA_latitude | interior spruce | AT3G15354.1 | SPA1-related 3 |
| Potri.001G317600 | RDA_latitude | interior spruce | AT1G04920.1 | sucrose phosphate synthase 3F |
| Potri.017G057800 | RDA_latitude | interior spruce | AT1G04920.1 | sucrose phosphate synthase 3F |
| Potri.015G140600 | RDA_latitude | interior spruce | AT5G52190.1 | Sugar isomerase (SIS) family protein |
| Potri.011G030900 | RDA_latitude | interior spruce | AT1G61740.1 | Sulfite exporter TauE/SafE family protein |
| Potri.014G131900 | RDA_latitude | interior spruce | AT1G02910.1 | tetratricopeptide repeat (TPR)-containing protein |
| Potri.001G326800 | RDA_latitude | interior spruce | AT5G14080.1 | Tetratricopeptide repeat (TPR)-like superfamily protein |
| Potri.002G139400 | RDA_latitude | interior spruce | AT1G02150.1 | Tetratricopeptide repeat (TPR)-like superfamily protein |
| Potri.014G050300 | RDA_latitude | interior spruce | AT1G02150.1 | Tetratricopeptide repeat (TPR)-like superfamily protein |
| Potri.006G256400 | RDA_latitude | interior spruce | AT2G25320.1 | TRAF-like family protein |
| Potri.003G096100 | RDA_latitude | interior spruce | AT5G46910.1 | Transcription factor jumonji (jmj) family protein |
| Potri.014G172300 | RDA_latitude | interior spruce | AT4G11120.1 | translation elongation factor Ts (EF-Ts), putative |
| Potri.003G084200 | RDA_latitude | interior spruce | AT1G27170.1 | transmembrane receptors;ATP binding |
| Potri.006G269900 | RDA_latitude | interior spruce | AT1G27170.1 | transmembrane receptors;ATP binding |
| Potri.011G008600 | RDA_latitude | interior spruce | AT1G27170.1 | transmembrane receptors;ATP binding |
| Potri.011G008700 | RDA_latitude | interior spruce | AT1G27170.1 | transmembrane receptors;ATP binding |
| Potri.011G008800 | RDA_latitude | interior spruce | AT1G27170.1 | transmembrane receptors;ATP binding |
| Potri.011G012500 | RDA_latitude | interior spruce | AT1G27170.1 | transmembrane receptors;ATP binding |
| Potri.011G012900 | RDA_latitude | interior spruce | AT1G27170.1 | transmembrane receptors;ATP binding |
| Potri.011G013400 | RDA_latitude | interior spruce | AT1G27170.1 | transmembrane receptors;ATP binding |
| Potri.011G013500 | RDA_latitude | interior spruce | AT1G27170.1 | transmembrane receptors;ATP binding |
| Potri.011G013900 | RDA_latitude | interior spruce | AT1G27170.1 | transmembrane receptors;ATP binding |
| Potri.011G014500 | RDA_latitude | interior spruce | AT1G27170.1 | transmembrane receptors;ATP binding |
| Potri.011G015400 | RDA_latitude | interior spruce | AT1G27170.1 | transmembrane receptors;ATP binding |
| Potri.011G060400 | RDA_latitude | interior spruce | AT1G27170.1 | transmembrane receptors;ATP binding |
| Potri.011G060600 | RDA_latitude | interior spruce | AT1G27170.1 | transmembrane receptors;ATP binding |
| Potri.015G043500 | RDA_latitude | interior spruce | AT1G27170.1 | transmembrane receptors;ATP binding |
| Potri.T112200 | RDA_latitude | interior spruce | AT1G27170.1 | transmembrane receptors;ATP binding |
| Potri.004G168000 | RDA_latitude | lodgepole pine | AT2G21170.1 | triosephosphate isomerase |
| Potri.001G471200 | RDA_latitude | interior spruce | AT5G53300.1 | ubiquitin-conjugating enzyme 10 |
| Potri.004G175000 | RDA_latitude | interior spruce | AT5G53300.1 | ubiquitin-conjugating enzyme 10 |
| Potri.007G102500 | RDA_latitude | interior spruce | AT1G55860.1 | ubiquitin-protein ligase 1 |
| Potri.011G094100 | RDA_latitude | interior spruce | AT1G55860.1 | ubiquitin-protein ligase 1 |
| Potri.008G037800 | RDA_latitude | interior spruce | AT5G04240.1 | Zinc finger (C2H2 type) family protein |
| Potri.010G224700 | RDA_latitude | interior spruce | AT5G04240.1 | Zinc finger (C2H2 type) family protein |
| Potri.003G148400 | RDA_latitude | interior spruce | AT2G38970.1 | Zinc finger (C3HC4-type RING finger) family protein |
| Potri.006G254000 | RDA_latitude | lodgepole pine | AT5G25360.1 |  |
| Potri.007G011800 | RDA_latitude | lodgepole pine | AT5G65540.1 |  |
| Potri.002G089100 | RDA_latitude | interior spruce | AT1G36990.1 |  |
| Potri.015G078200 | RDA_latitude | interior spruce | AT5G62960.1 |  |
