# Supplementary material for: Phenotypic and Genomic Local Adaptation across Latitude and Altitude in Populus trichocarpa
Source: Genome Biol Evol. 2019 Jul 22;11(8):2256–72. doi: 10.1093/gbe/evz151 (PMC6735766; doi:10.1093/gbe/evz151)
Supplement: evz151_Supplementary_Data [file evz151_supplementary_data.zip › Zhang_et_al_supplemental_figures_R1.pdf]

Figure S1. Biplot of principal component analysis for latitudinal samples (A) and altitudinal samples (B).

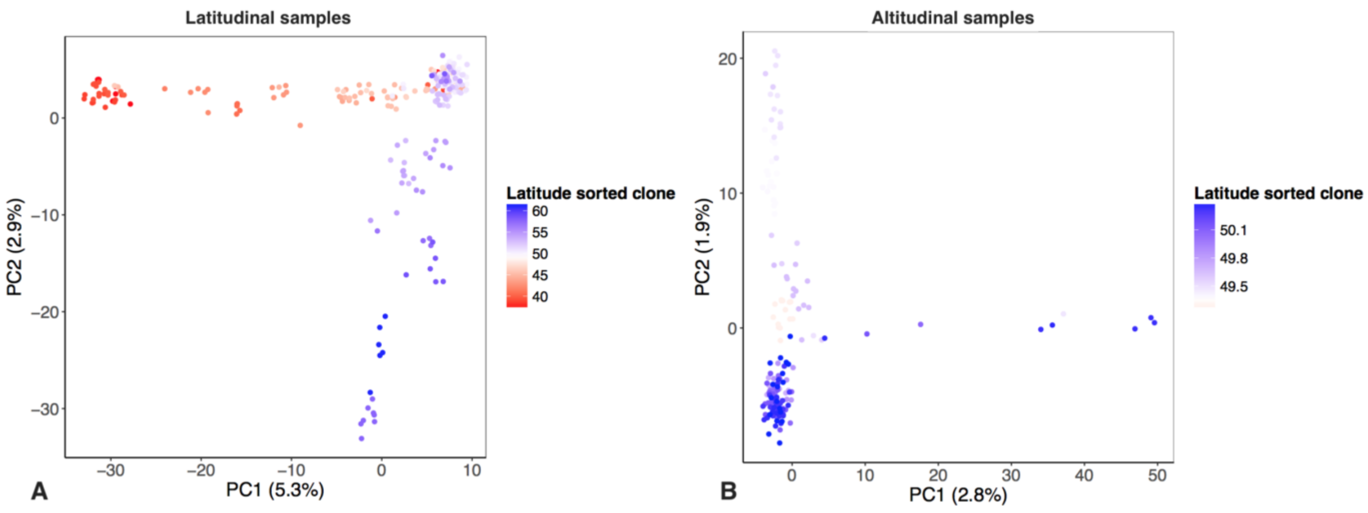

Figure S2-1. Quantile-quantile plot of phenotypic associations from the best structured model among altitudinal samples. Phenotypic traits include: height in VA garden, height in BC garden, timing of bud set in VA garden, timing of bud set in BC garden, timing of bud flush in VA garden, timing of bud flush in BC garden, diameter in VA garden, regeneration height in VA garden, regenerated branch number in VA garden, cold injury in VA garden.

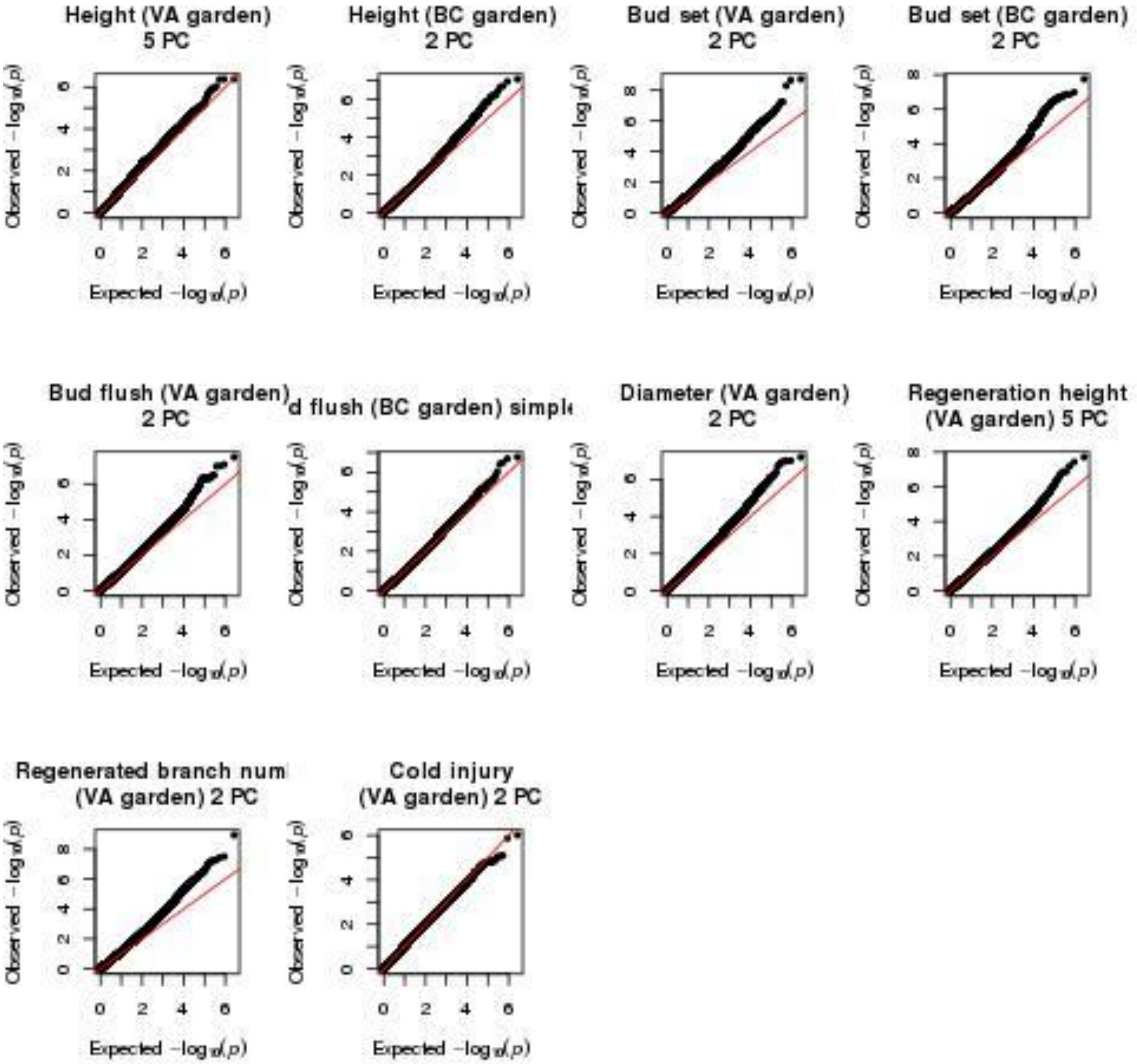

Figure S2-2. Quantile-quantile plot of phenotypic associations from the best structured model among latitudinal samples.

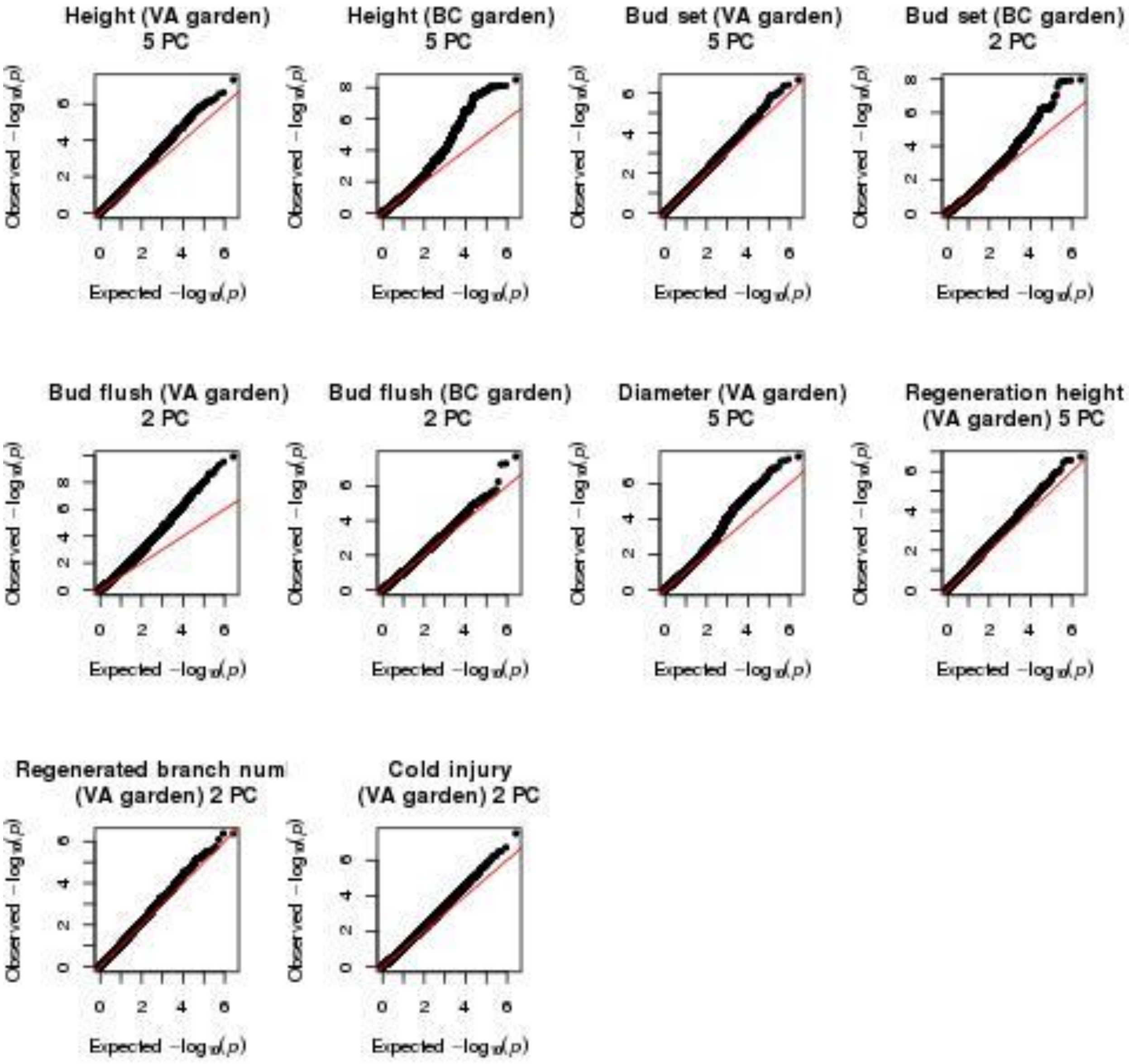

Figure S3. Phenotype BLUPs in relation to latitude across latitudinal samples. Traits include timing of bud flush (A), height (C), diameter (E), cold injury (F), regeneration height (G), regenerated branch number (H) in VA garden, and timing of bud flush (B), height (D) in BC garden.

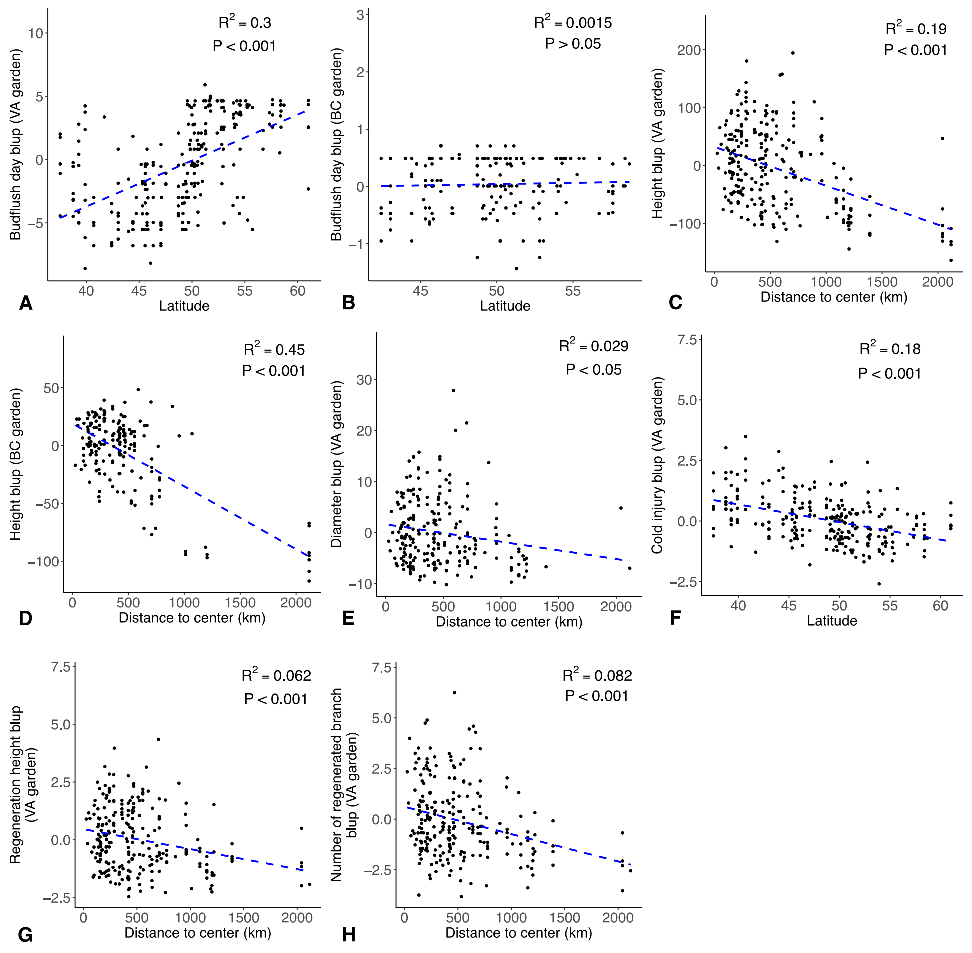

Figure S4. Phenotype BLUPs in relation to elevation across altitudinal samples. Traits include timing of bud flush (A), height (C), diameter (E), cold injury (F), regeneration height (G), regenerated branch number (H) in VA garden, and timing of bud flush (B), height (D) in BC garden.

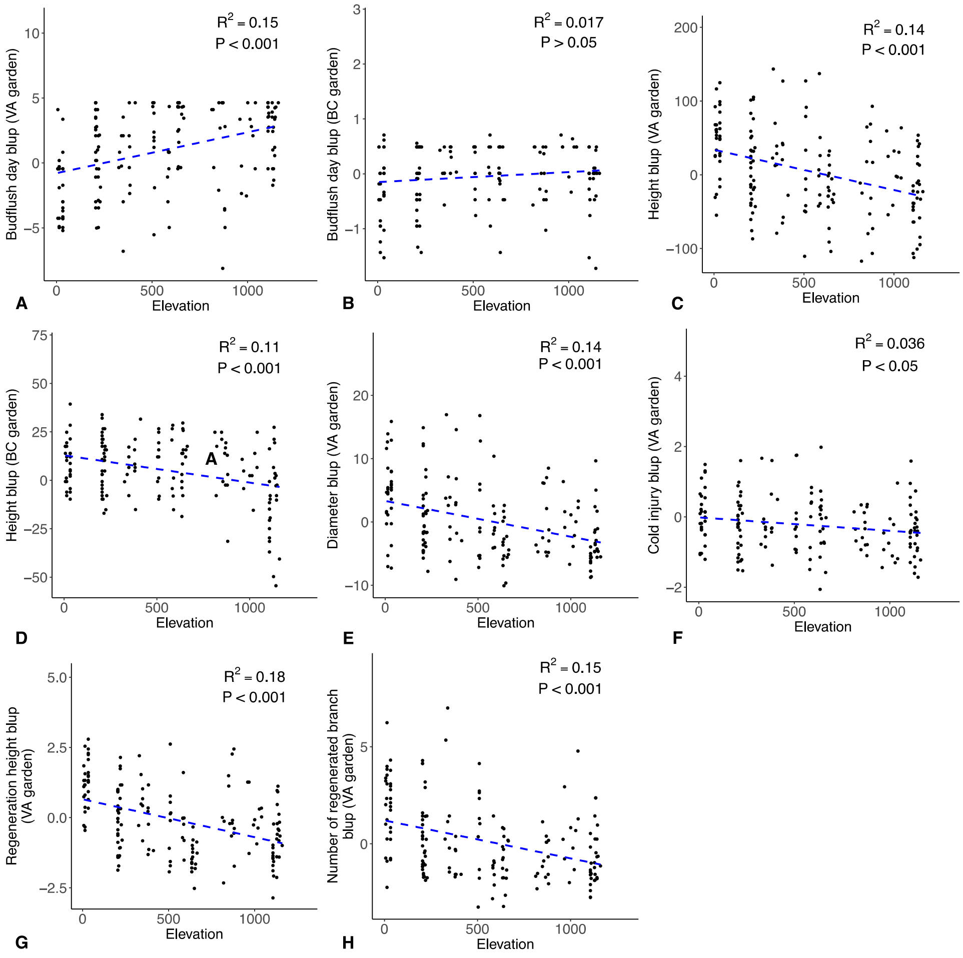

Figure S5. Heatmap of correlations between phenotypic traits, geographical and climatic variables across altitude transect (A) and latitude transect (B). Pearson's correlation coefficients of 1 are indicated by color red and -1 indicated by blue.

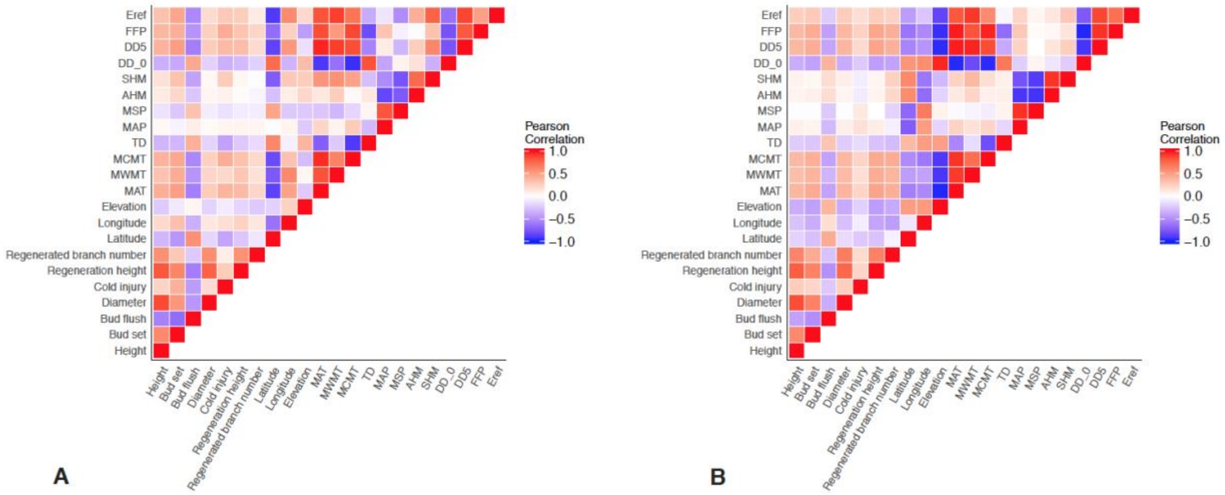

Figure S6. Gene-based analysis identifies top candidate genes associated with phenotypic traits among altitudinal samples. Traits include: height (A), diameter (C), timing of bud flush (D), cold injury (G), regeneration height (H), regenerated branch number (I) in VA garden, and height (B), timing of bud flush (E), timing of bud set (F) in BC garden (B). The red line is the expected number of SNP outliers based on 99.9% binomial quantile given total number of SNPs within each gene or genomic region. The red dots are candidate genes or intergenic regions with enriched number of SNP outliers.

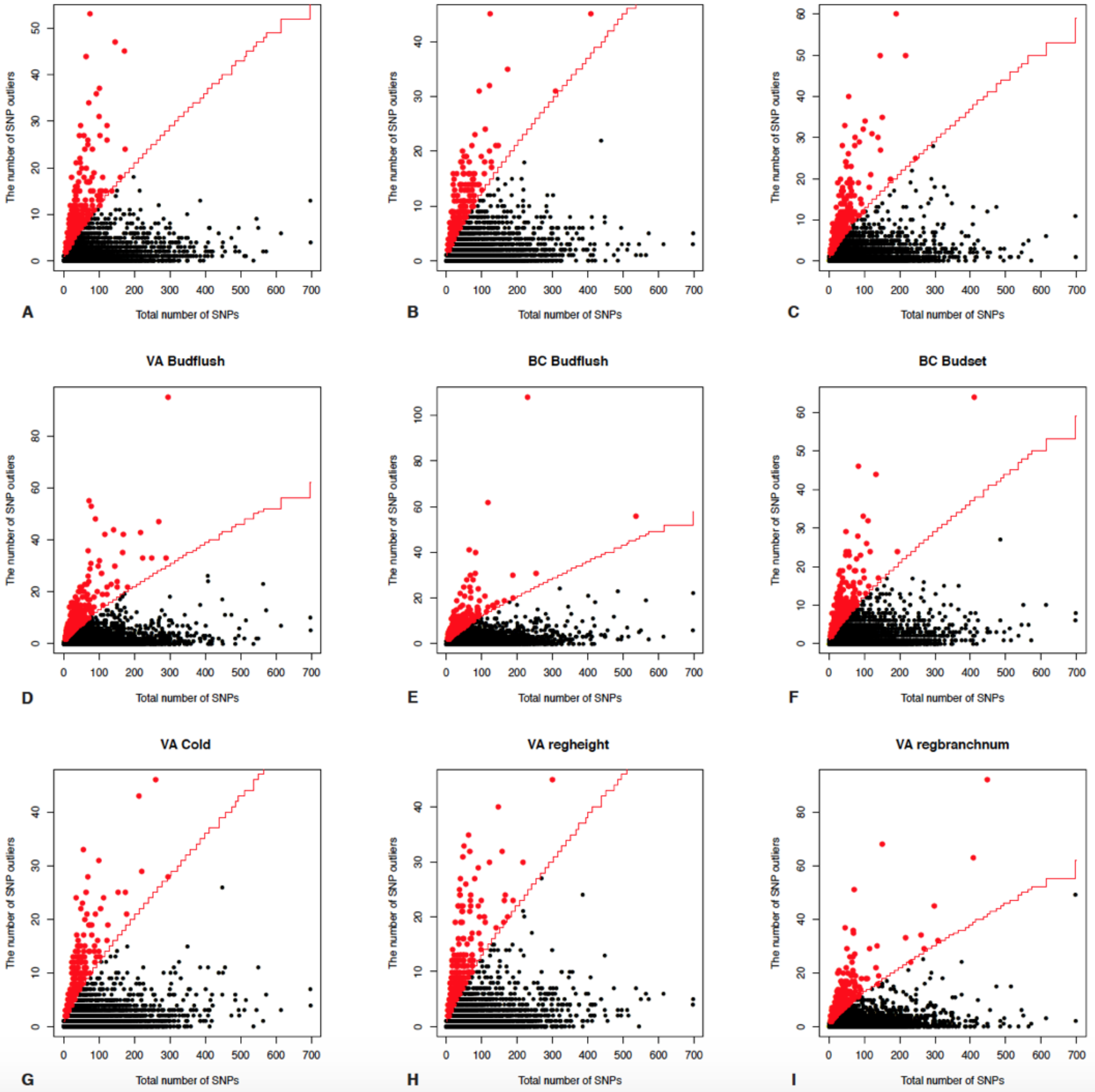

Figure S7. Gene-based analysis identifies top candidate genes associated with phenotypic traits among latitudinal samples. Traits include: height (A), diameter (C), timing of bud flush (D), cold injury (G), regeneration height (H), regenerated branch number (I) in VA garden, and height (B), timing of bud flush (E), timing of bud set (F) in BC garden (B). The red line is the expected number of SNP outliers based on 99.9% binomial quantile given total number of SNPs within each gene or genomic region. The red dots are candidate genes or intergenic regions with enriched number of SNP outliers.

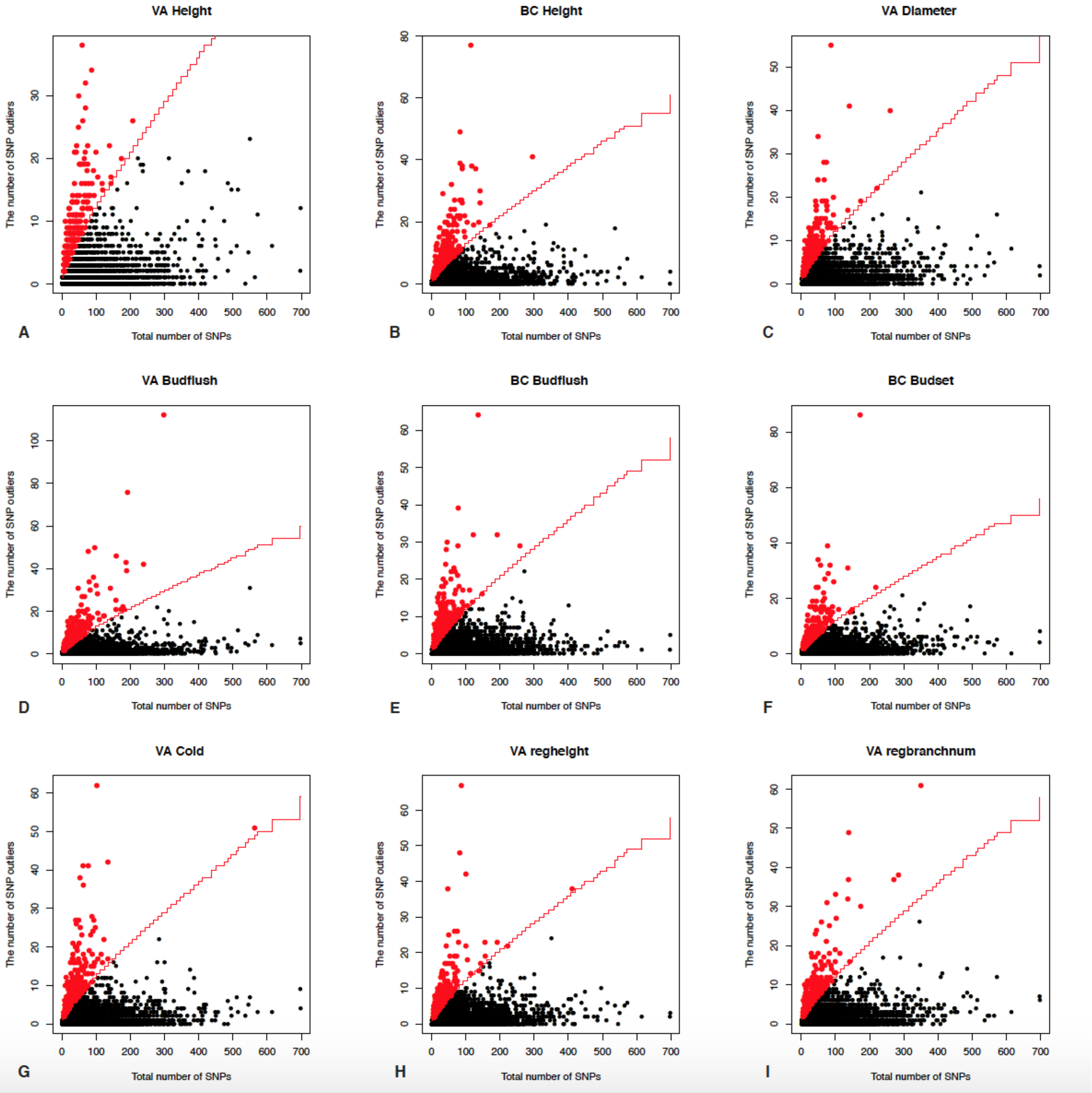

Figure S8. Manhattan plot showing gene-wise p-values obtained in the association analysis in altitudinal samples.

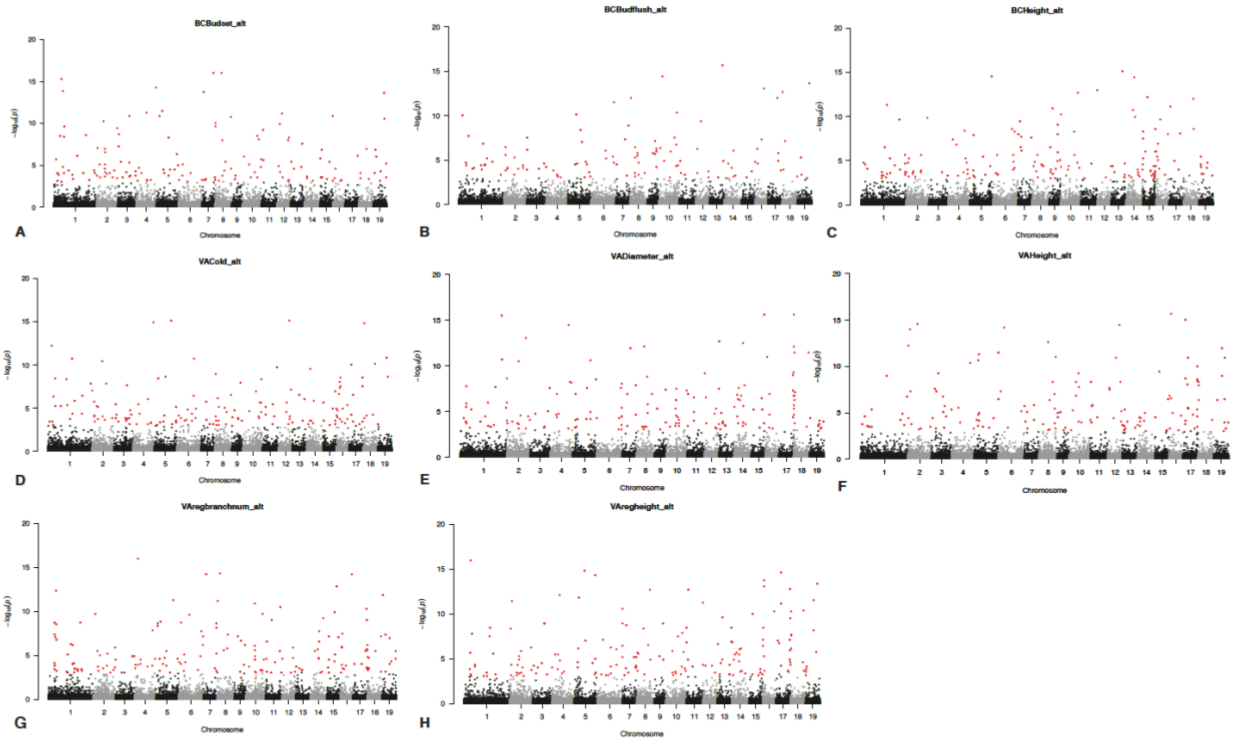

Figure S9. Manhattan plot showing gene-wise p-values obtained in the association analysis in latitudinal samples.

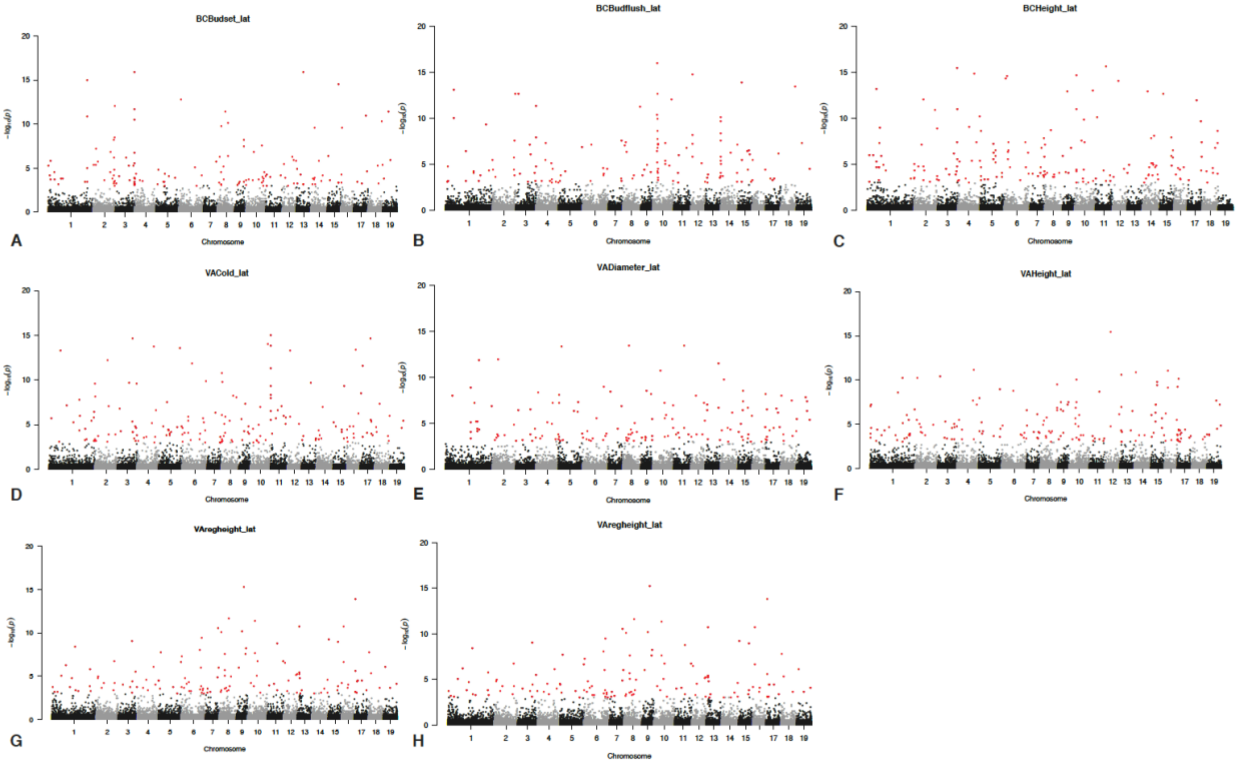

Figure S10. Gene-based identification of top candidate genes associated with climate variables along altitude transect. Climate variables include Latitude (A), Longitude (B), Elevation (C), MWMT (D), MCMT (E), TD (F), MAP (G), MSP (H), AHM (I), SHM (J), DD_0 (K), DD5 (L), DD_18 (M), DD18 (N), NFFD (O), bFFP (P), eFFP (Q), FFP (R), PAS (S), EMT (T), EXT (U), Eref (V), CMD (W).

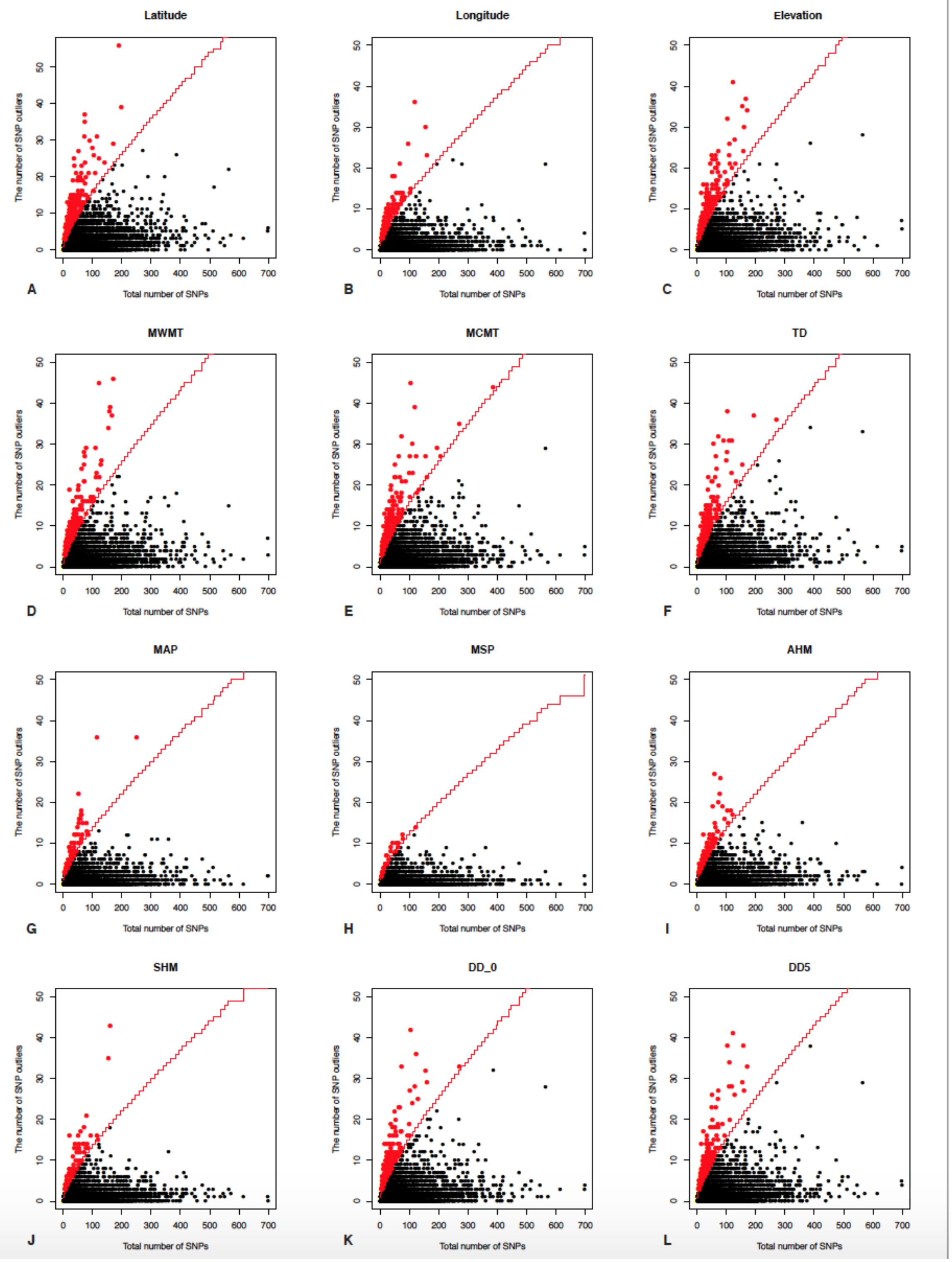

Figure S10. (Continued)

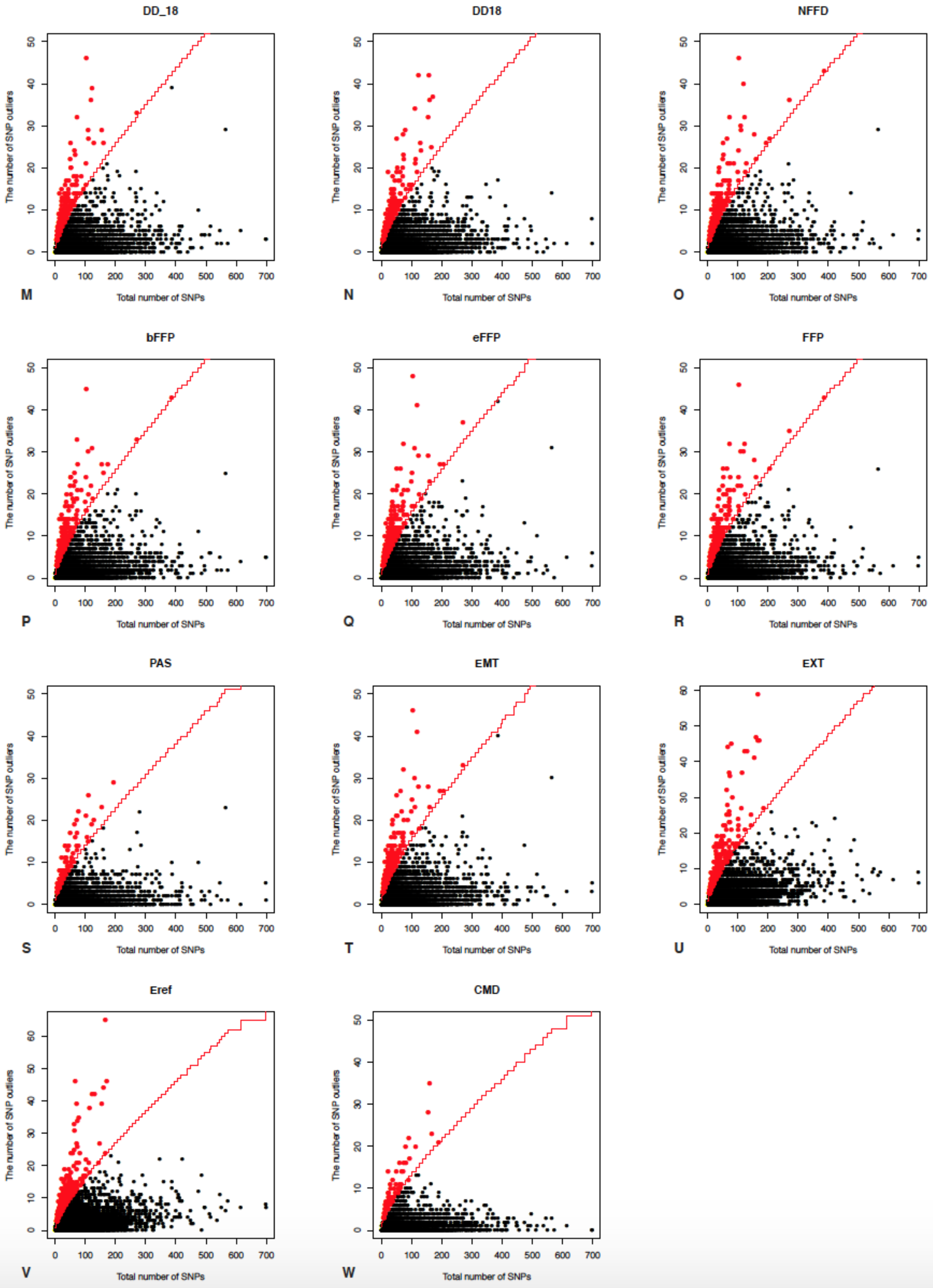

Figure S11. Gene-based identification of top candidate genes associated with climate variables along latitude transect. Climate variables include Latitude (A), Longitude (B), Elevation (C), MWMT (D), MCMT (E), TD (F), MAP (G), MSP (H), AHM (I), SHM (J), DD_0 (K), DD5 (L), DD_18 (M), DD18 (N), NFFD (O), bFFP (P), eFFP (Q), FFP (R), PAS (S), EMT (T), EXT (U), Eref (V), CMD (W).

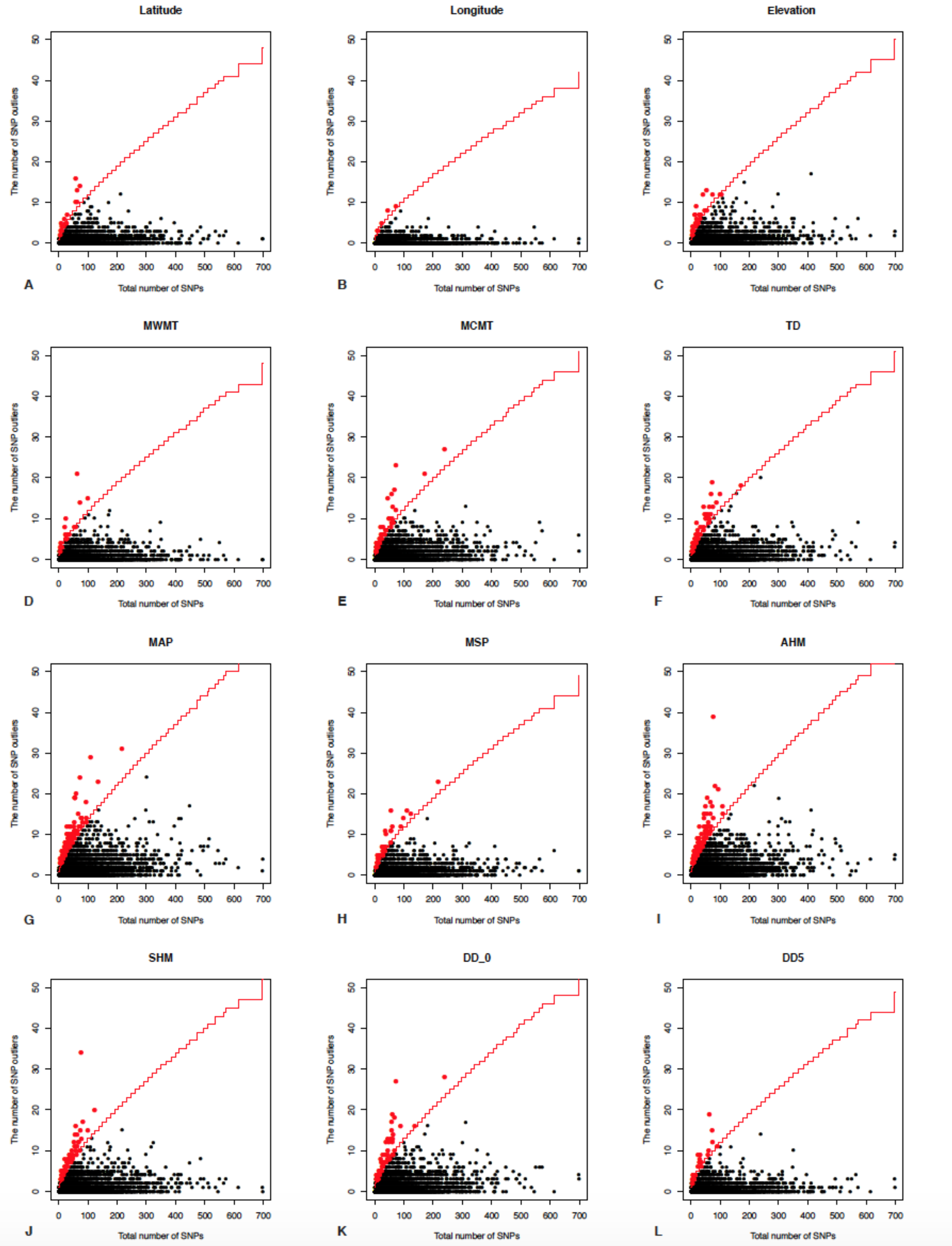

Figure S11. (Continued)

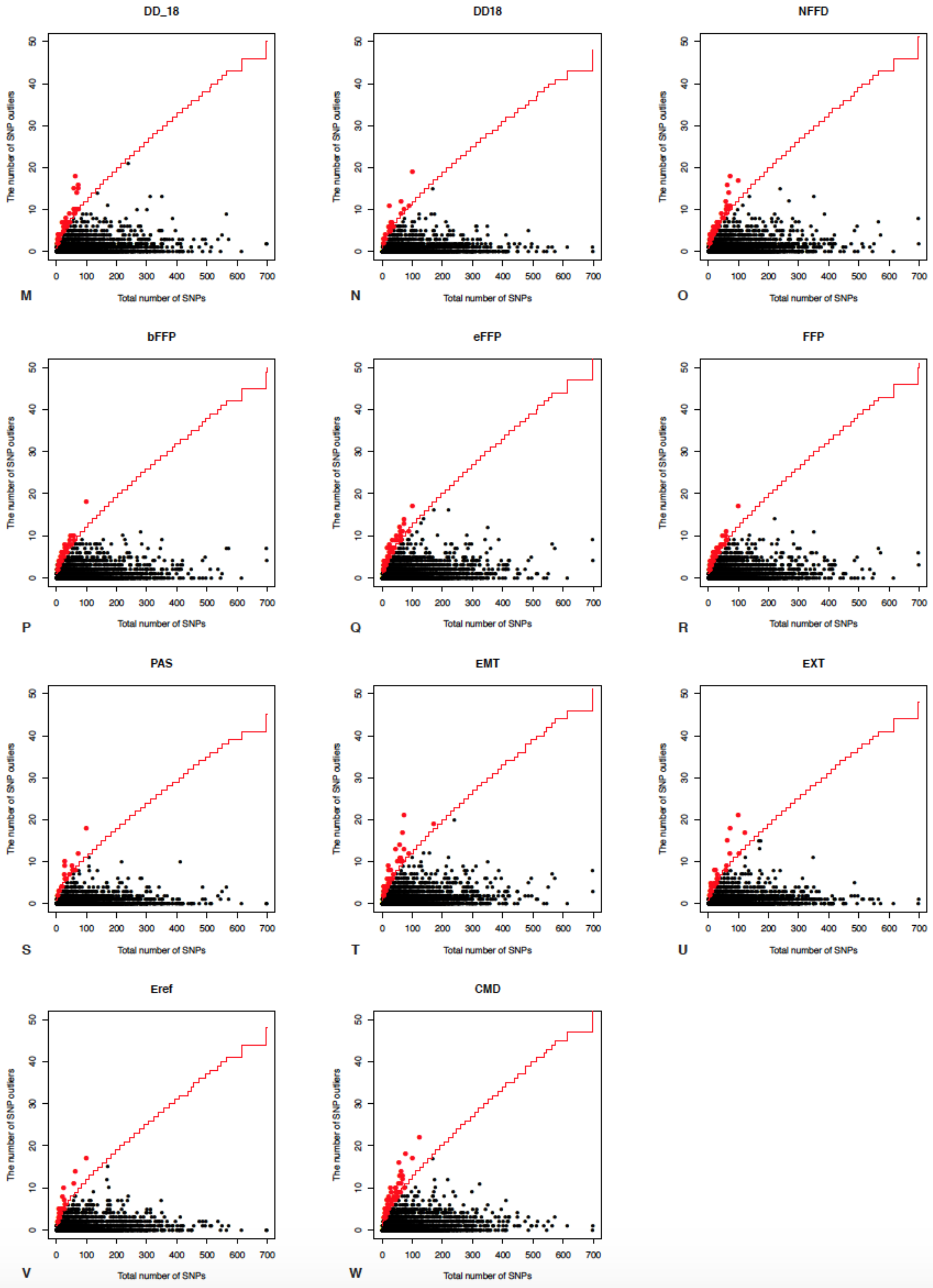

Figure S12. Triplots of SNP loadings on RDA axis 1 and 3 along altitude transect (A) and latitude transect (B). The points represent candidate SNPs colored by most highly correlated environmental predictor. Blue vectors represent environmental predictors.

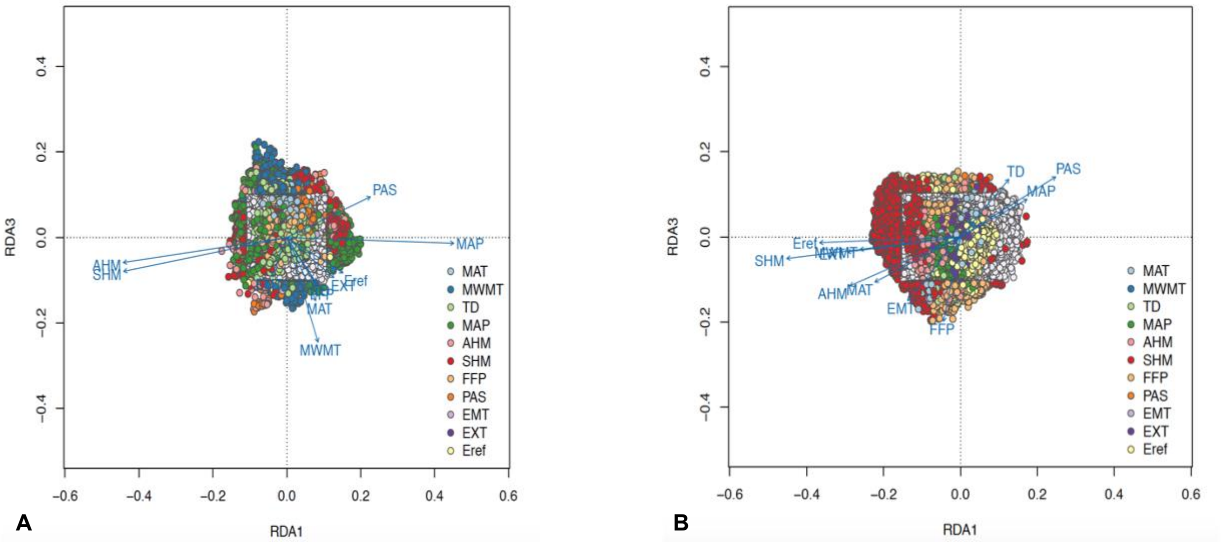

Figure S13. Venn diagram of candidate genes identified by gene-based anlaysis of structured GWAS and Bayenv scans across altitude and latitude transect.

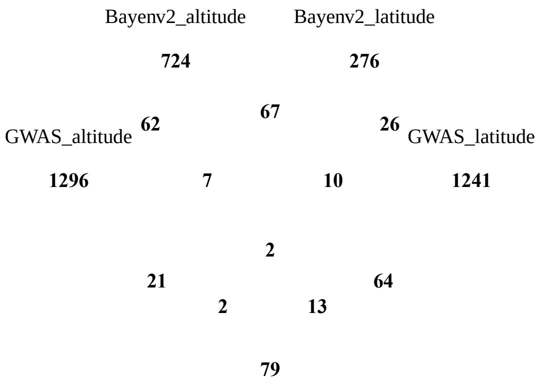

Figure S14. Venn diagram showing overlap between our phenotype-associated candidate genes and two other studies. Our candidate genes refer to timing of bud flush (A) and height (B) associated genes identified from the gene-based analysis across both transects. Genes from other studies refer to genes associated with timing of bud flush (A) and height (B) identified from two other GWAS studies (Evans *et al.*, 2014; McKown *et al.*, 2014).

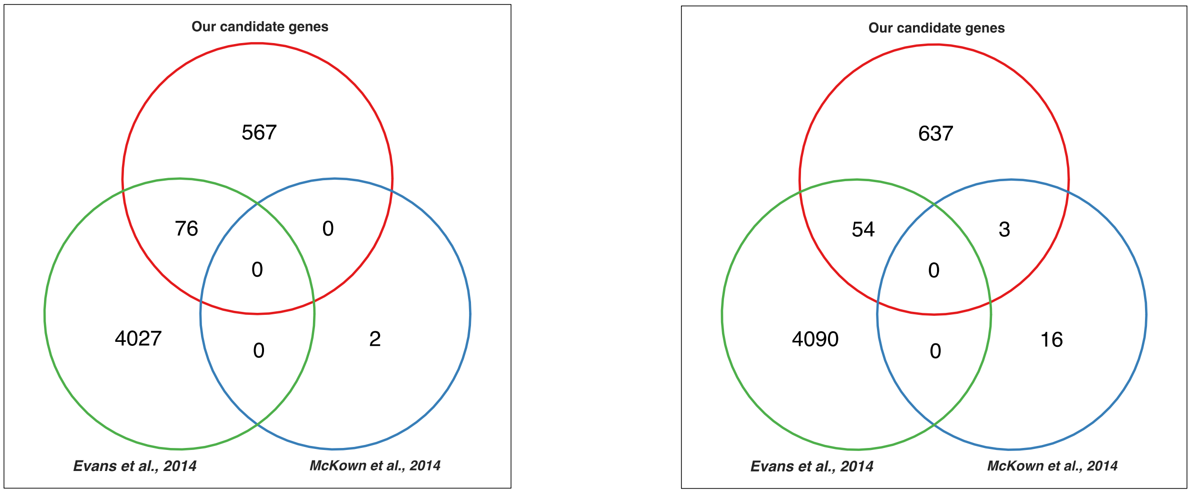

A

B
